# Comparative effects of different whole grains and brans on blood lipid: a network meta-analysis

**DOI:** 10.1007/s00394-018-1827-6

**Published:** 2018-09-22

**Authors:** Suocheng Hui, Kai Liu, Hedong Lang, Yang Liu, Xiaolan Wang, Xiaohui Zhu, Steve Doucette, Long Yi, Mantian Mi

**Affiliations:** 1 Research Center for Nutrition and Food Safety, Institute of Military Preventive Medicine, Third Military Medical University, Chongqing Key Laboratory of Nutrition and Food Safety, Chongqing Medical Nutrition Research Center, Chongqing, 400038 People’s Republic of China; 2 Department of Health Supervision, Center for Disease Control and Prevention of Shenyang Joint Logistic Support Center, Shenyang, 110034 People’s Republic of China; 3 Department of Community Health and Epidemiology, Dalhousie University, Halifax, Nova Scotia Canada

**Keywords:** Whole grain, Bran, Blood lipid, Network meta-analysis

## Abstract

**Purpose:**

The comparative effects of different whole grains and brans on blood lipid are still not totally elucidated. We aimed to estimate and rank the effects of different whole grains and brans on the control of blood lipid.

**Methods:**

We performed a strategic literature search of PubMed, EMBASE and the Cochrane Library for relevant trials. Both pairwise meta-analyses and network meta-analyses were conducted to compare and rank the intervention strategies of whole grains and brans for the control of total cholesterol (TC), LDL cholesterol (LDL-C), HDL cholesterol (HDL-C), and triglycerides (TG).

**Results:**

Fifty-five eligible trials with a total of 3900 participants were included. Cumulative ranking analyses showed that oat bran was the most effective intervention strategy for TC and LDL-C improvements, with significant decreases of − 0.35 mmol/L (95% CI − 0.47, − 0.23 mmol/L) and − 0.32 mmol/L (95% CI − 0.44, − 0.19 mmol/L) in TC and LDL-C compared with control, respectively. In comparison with control, oat was associated with significant reductions in TC by − 0.26 mmol/L (95% CI − 0.36, − 0.15 mmol/L) and LDL-C by − 0.17 mmol/L (95% CI − 0.28, − 0.07 mmol/L), which was ranked as the second best treatment. Barley, brown rice, wheat and wheat bran were shown to be ineffective in improving blood lipid compared with control.

**Conclusions:**

This network meta-analysis suggests that oat bran and oat are ranked higher than any other treatments for the regulations of TC and LDL-C, indicating that increasing oat sources of whole grain may be recommended for lipid control.

**Electronic supplementary material:**

The online version of this article (10.1007/s00394-018-1827-6) contains supplementary material, which is available to authorized users.

## Introduction

According to the latest estimates, 31% of all global deaths (17.7 million) are due to cardiovascular diseases (CVDs) [1]. The management of CVDs is costly and prolonged, which has brought heavy burden to the public health [2, 3]. Several previous studies demonstrated that effectively control of lipid metabolism inhibited the development and progression of CVDs [4, 5]. In addition, it has been suggested that 1% decreases in total cholesterol (TC) and LDL cholesterol (LDL-C) can reduce the risk of CVDs by 3% and 1%, respectively [6]. Recently, dietary intervention strategies have received increasing attention in the prevention and treatment of CVDs because they may have less adverse effects and are more cost-effective compared with the lipid lowering drugs [7, 8].

Among various dietary adjustment strategies, increasing whole grains and brans intake has been widely investigated in the area of blood lipid control [9, 10]. Common whole grains and brans used in the previous clinical trials included oat, wheat, brown rice, barley, oat bran and wheat bran [11, 12]. However, these different intervention strategies have yielded variable results and which type is superior to others in lipid control are still inconclusive.

There is no individual RCT investigating the comparative effects of different whole grains and brans on blood lipid, which might because it is rather costly to conduct an individual trial with more than three arms [13]. Additionally, the traditional meta-analysis methods do not allow different comparators to be compared simultaneously and thus they have limited ability to rank the relative effectiveness of different interventions [13, 14]. However, network meta-analysis incorporates both direct and indirect comparisons of different intervention strategies, including those that have never been compared directly in head-to-head trials, which can answer questions more broadly than pairwise meta-analysis [15]. Therefore, we conducted a network meta-analysis to adequately assess and rank the comparative effects of different whole grains and brans on the control of blood lipid.

## Materials and methods

### Literature search

The study was conducted according to the prespecified study protocol and preferred reporting items for systematic reviews and meta-analyses (PRISMA) extension statement for reporting of network meta-analyses of health care interventions [16]. PubMed (updated to July 2018; http://www.ncbi.nlm.nih.gov/pubmed/), Embase (updated to July 2018; http://www.embase.com/search/advanced/) and the Cochrane Library (updated to July 2018; http://www.cochrane.org/) were searched for human studies. Following terms were searched in all fields: whole grain, wholegrain, whole meal, whole wheat, wheat, rice, brown rice, wild rice, maize, oat, barley, corn, rye, millet, sorghum, triticale, canary seed, amaranth, buckwheat or quinoa which were paired with the following words: lipid profile, lipid distribution, blood lipid, cholesterol, total cholesterol, TC, low density lipoprotein, LDL, LDL-C, high density lipoprotein, HDL, HDL-C, triglyceride, triacylglycerol, triglyceride, TG, TAG or lipoprotein. In addition, to further identify eligible trials, we hand-searched reference lists in the reviews and included studies. The full search strategy was described in Supplemental materials and methods. The literature search and study selection were conducted by two independent reviewers in parallel (SC-H and K-L), and any discrepancies were resolved by a third investigator (MT-M).

### Study selection

We selected studies that met the following criteria: (1) treatment duration lasted more than 2 weeks; (2) the studies enrolled apparently healthy or high-risk CVDs population (subjects with known dyslipidemia, hyperglycemia, hypertension, overweight or obesity, or a combination of these factors) and not diagnosed with CVDs [17, 18]; (3) the baseline and post-intervention values or change scores for TC, LDL-C, HDL-C, or TG with their standard deviations (SDs), standard errors (SEs) or 95% CIs were available for each group in the study; (4) participants received the intervention of barley, brown rice, oat, oat bran, rye, rye bran, wheat, wheat bran alone or control (refined-grain diets or products); (5) the trial compared 2 or more different intervention strategies; (6) the treatment products were not given as multi-components and the effects of whole grains and brans could be distinguished.

### Risk of bias and quality of evidence

The risk of bias was assessed using the Cochrane Risk of Bias assessment tool. The assessment items included adequacy of sequence generation, allocation concealment, blinding, blinding of outcome assessments, incomplete outcome data, selective reporting, and other biases [19]. Additionally, the quality of evidence was assessed using the Grading of Recommendations Assessment, Development and Evaluation (GRADE) framework, which characterizes the evidence on the basis of the study limitations, imprecision, inconsistency, indirectness, and publication bias [20]. Two researchers (SC-H and K-L) independently reviewed the studies and judged the risk of bias and quality of evidence. Any discrepancies were resolved by consensus and arbitration by a third investigator (MT-M).

### Data extraction

We reviewed the included articles and the following items were extracted: (1) study characteristics including information of authors, publication year, region, sample size, study design, treatment duration, treatment products and dietary fiber content of intervention products; (2) population information on age and body mass index (BMI); (3) change scores (primary values) or baseline and post-intervention values in TC, LDL-C, HDL-C and TG; (4) all values were converted to mmol/L using the conversion factors 1 mg/dL = 0.02586 mmol/L for TC, LDL-C, HDL-C, and 1 mg/dL = 0.01129 mmol/L for TG [21]. For crossover design trials, we extracted the data of two phases [19]. In parallel design studies, all treatment outcomes at different visits were extracted and used to estimate the intervention effects.

### Statistical analysis

We performed the pairwise meta-analysis using the random-effects model. The outcomes of treatments were estimated using mean differences (MDs). The *I*^2^ statistic and *P* value were calculated to identify the heterogeneity among the included studies [22]. In addition, the Egger’s test was used to detect the small-study effects.

We excluded percentage changes in mean and SD values when we extracted data for the meta-analysis. If SD values were not reported in the studies, we calculated them from SEs, 95% CIs, *P* values, or *t* statistics. Besides, change-from-baseline SD values were calculated by assuming a correlation coefficient of 0.5 [23].

Frequentist model network meta-analyses were conducted to estimate the comparative effects of different whole grains and brans on the control of blood lipid if at least five treatments arms were available across the studies [24]. Ranking probabilities of treatments were evaluated using surface under the cumulative ranking curve (SUCRA) and mean ranks. The SUCRA accounts both for the variance and the location of all relative treatment effects. The larger the SUCRA value, the better the rank of the treatment (0% = worst; 100% = best) [25, 26].

Transitivity is the fundamental premise underlying network meta-analysis [13, 14, 27]. We evaluated whether the transitivity assumption is valid by assessing the inconsistency between direct and indirect evidence. We assessed the local inconsistency using the loop-specific approach and node-splitting method [28, 29]. To assess the evidence of inconsistency in the entire network, we used the design-by-treatment model [30]. We used the comparison-adjusted funnel plot to visually assess the evidence for publication bias in the network [25], and the comparisons were presented as treatment alphabetically earlier versus later treatment.

To assess the robustness of the findings, we performed sensitivity analyses by fitting the inconsistency model as described by White [31, 32]. To explore the influence of study design on the results of network meta-analysis, we performed sensitivity analyses by only including the studies with a parallel design or removing the cluster crossover design trials. To explore the effect of treatment duration on the overall results of network meta-analysis, we conducted sensitivity analyses by excluding studies with the duration less than 3, 4 weeks or more than 12 weeks. In addition, we also conducted sensitivity analyses based on the studies that only included dyslipidemic participants, or low risk of bias.

We did the pairwise meta-analysis using meta package for R software and network meta-analysis with the method of multivariate meta-analysis in Stata version 14.0 using the mvmeta command and Stata routines described elsewhere [25, 31–34].

## Results

### Literature search

**Fig. 1:**
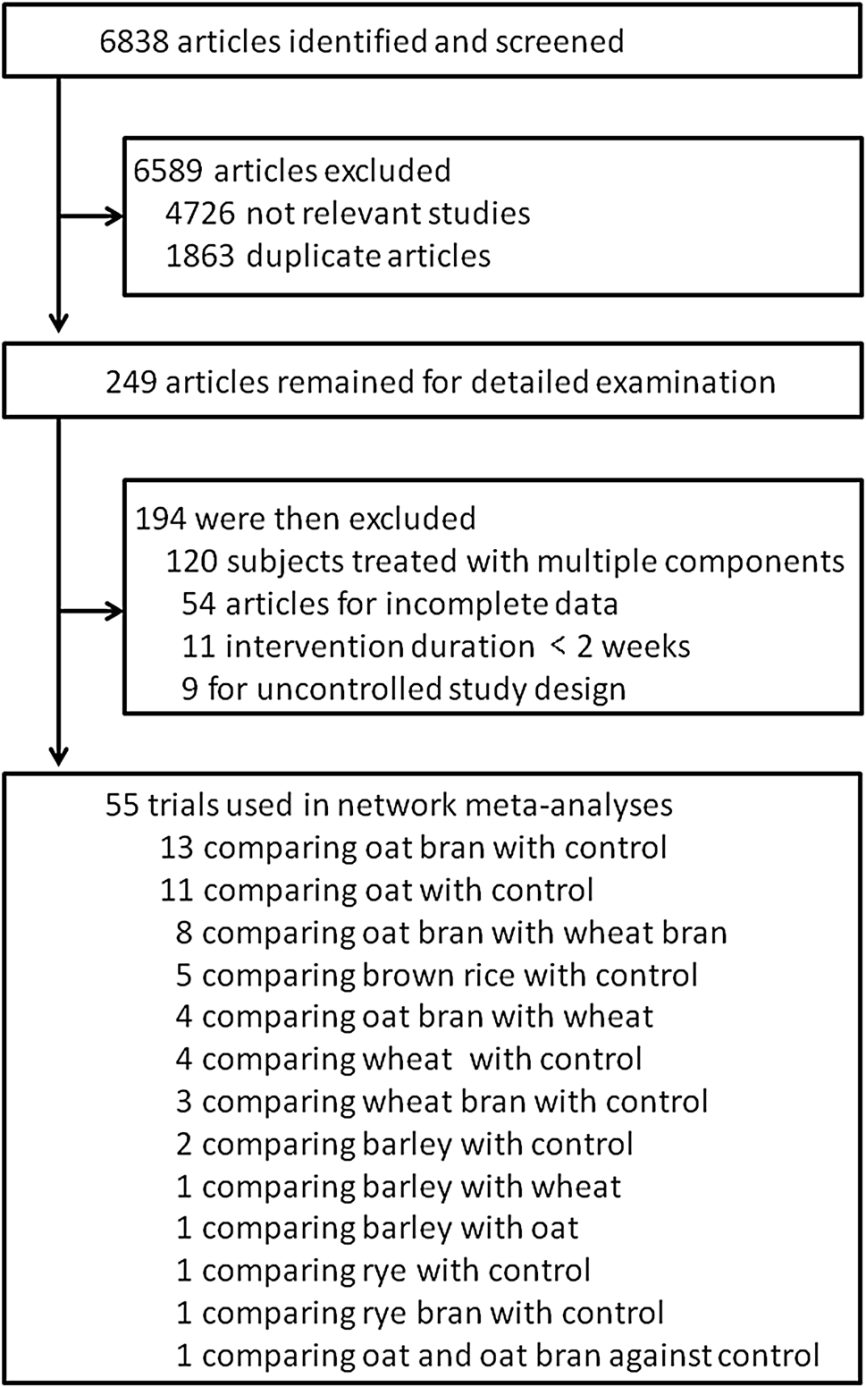
Flow diagram showing the number of citations retrieved in individual searches of articles included in the review

The detailed process of search strategy for this meta-analysis is shown in Fig. 1. A total of 6838 articles were identified in the initial search, and 6589 were excluded after reviewing the titles and abstracts. Among the excluded reports, 4726 were excluded because they were not relevant to the network meta-analysis and 1863 were excluded because they were duplicates. Therefore, 249 articles were remained for further detailed examinations. Among these 249 articles, an additional 194 were then excluded for the following reasons: 120 were excluded because they used multiple components, 54 were discarded because they had incomplete data, 11 were excluded because the treatment duration lasted less than 2 weeks, and 9 were ruled out because they used an uncontrolled study design. Thus, 55 studies were ultimately selected in this network meta-analysis.

### Study characteristics

A summary of study characteristics was presented in Supplemental Table 1. Fifty-five studies comprising a total of 3900 participants were included in the network meta-analysis. The total number of subjects included in each study ranged from 8 to 367. The BMI of the participants ranged from 19.2 to 30.9 kg/m^2^ (median 26.8 kg/m^2^). The age of the participants ranged from 20.4 to 63.0 years (median 51.0 years). Most of the studies (35 of 55) used the parallel design and the remained 20 studies used the crossover design. The intervention duration varied from 2 weeks to 12 months (median 6 weeks). Funding sources for all included studies were presented in Supplemental table 2.

### Risk of bias and quality of evidence

The risk of bias of the studies included in this network meta-analysis was generally low or unclear. Overall, 2 studies had a high-risk of bias for random sequence generation, 8 had unclear risk of bias on allocation concealment. 23 studies had a low risk of bias on blinding and 20 were in low risk of bias on blinding of outcome assessment. Most articles were in low risk of incomplete outcome data, selective reporting and other biases (Supplemental Figs. 1 and 2). The quality of evidence for all outcomes was rated as moderate or low for most comparisons. More details of the quality of evidence were presented in Supplemental tables 16, 30, 44 and 58.

### Pairwise meta-analysis

The pairwise meta-analysis showed that oat significantly reduced TC (− 0.30 mmol/L; 95% CI − 0.42, − 0.19 mmol/L) and LDL-C (− 0.16 mmol/L; 95% CI − 0.27, − 0.05 mmol/L) compared with control. Compared with control, significant decreases in TC (− 0.27 mmol/L; 95% CI − 0.43, − 0.12 mmol/L) and LDL-C (− 0.34 mmol/L; 95% CI − 0.63, − 0.05 mmol/L) were observed in the oat bran group (Supplemental Figs. 3 and 5). Compared with control, no remarkable improvements of HDL-C and TG were shown after the treatment of whole grains and brans (Supplemental Figs. 7 and 9). Additionally, most comparisons in all outcomes did not showed significant heterogeneity. Detailed results were summarized in Supplemental Tables 3, 17, 31 and 45.

### Network meta-analysis

**Fig. 2:**
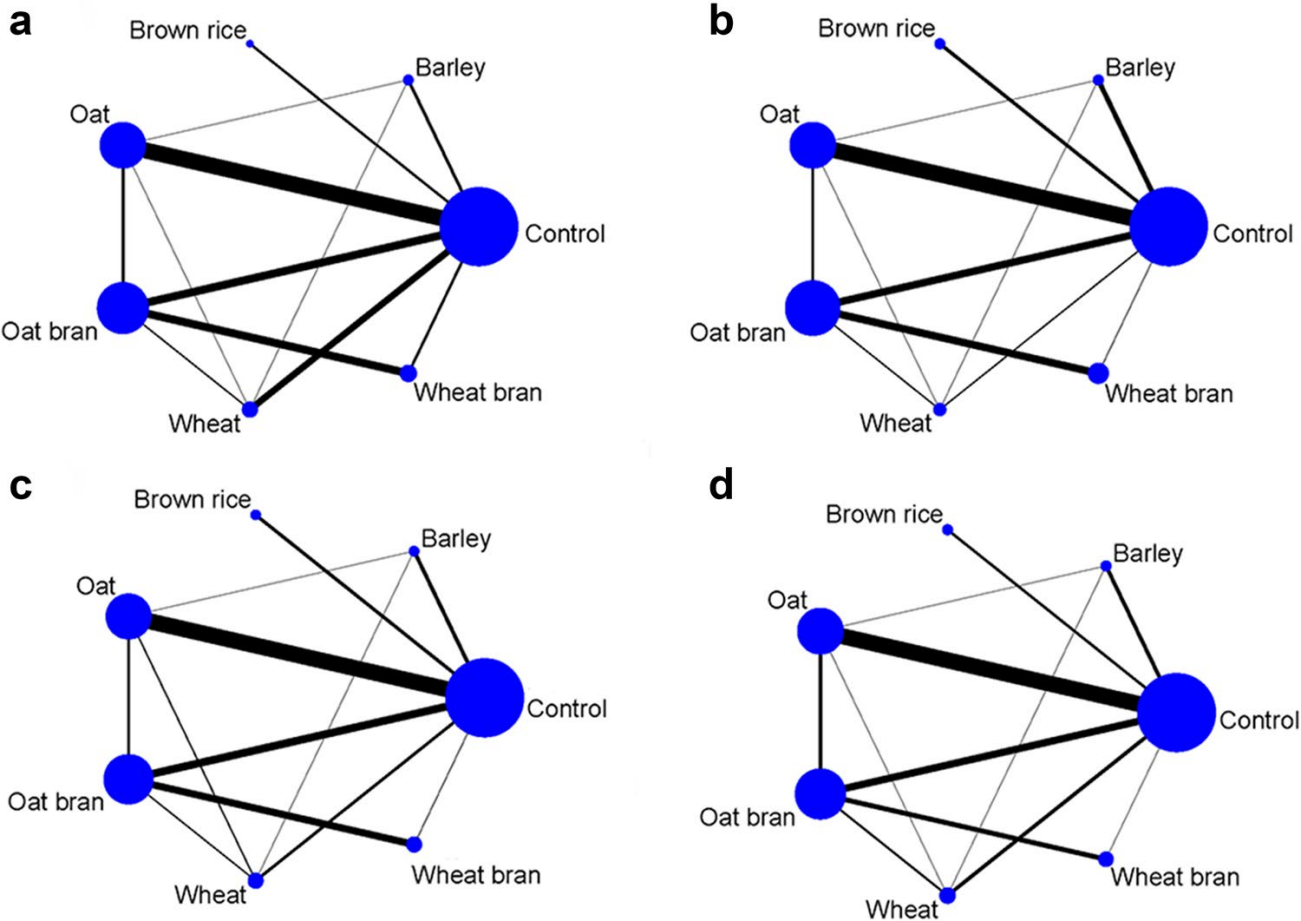
Network plots of eligible comparisons for different whole grains and brans for TC (**a**), LDL-C (**b**), HDL-C (**c**) and TG (**d**). Lines connect the interventions that have been studied in head-to-head (direct) comparisons in the eligible studies. The sizes of the nodes are weighted according to the number of trials that study the intervention, and the thickness line corresponds to the number of trials that assess direct comparisons between different interventions

**Fig. 3:**
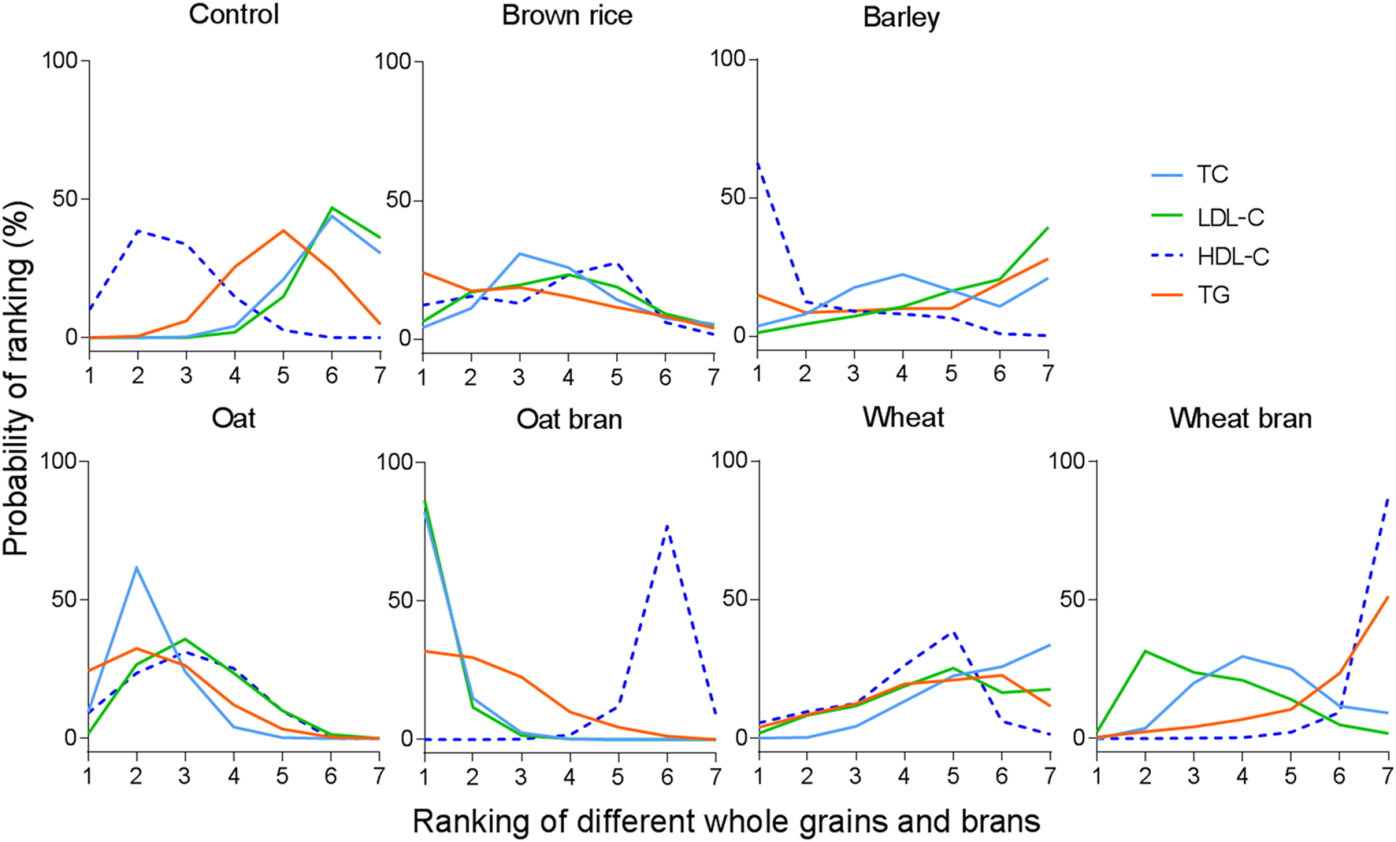
Ranking results of different whole grains and brans on the control of blood lipid. The *x*-axis represents the ranking of interventions which are ranked in numerical order, with the first representing the best. The *y*-axis represents the probability of each ranking. Each line indicates a type of intervention strategy

We did not conduct network meta-analysis for rye and rye bran because there was only one study available for analysis, respectively. The networks of eligible comparisons for TC, LDL-C, HDL-C and TG are shown in Fig. 2a–d, respectively. The network meta-analysis suggested that oat bran had the greatest likelihood of being the most effective treatment for TC, followed by oat (Fig. 3 and Supplemental Table 7). Consistent with the results of pairwise meta-analysis, compared with control, oat and oat bran significantly decreased TC by − 0.26 mmol/L (95% CI − 0.36, − 0.15 mmol/L) and − 0.35 mmol/L (95% CI − 0.47, − 0.23 mmol/L), respectively. In addition, oat bran and oat were superior to wheat in reducing TC. Barley, brown rice, wheat and wheat bran showed insignificant effects on TC. Detailed results of network meta-analysis on TC are shown in Supplemental Table 6.

As presented in Fig. 3 and Supplemental Table 21, results of the network meta-analysis on LDL-C demonstrated that oat bran was the highest ranked treatment strategy for the reduction of LDL-C, followed by oat. Detailed results were available in Supplemental Table 20. Compared with control, oat and oat bran significant reduced LDL-C by − 0.17 mmol/L (95% CI − 0.28, − 0.07 mmol/L) and − 0.32 mmol/L (95% CI − 0.44, − 0.19 mmol/L), respectively. Besides, oat bran was more effective than brown rice and wheat for LDL-C reductions. The same to the results of TC, increasing the consumption of barley, brown rice, wheat or wheat bran was not associated with reduction of LDL-C.

Estimated effects of different whole grains and brans on HDL-C were presented in Supplemental Table 34. Based on the ranking results of HDL-C, brown rice appeared to be the most effective intervention, and oat was ranked after brown rice (Fig. 3 and Supplemental Table 35). However, compared with control, consumption of different whole grains and brans showed no pronounced beneficial effects on HDL-C.

Consistent with the results of TC and LDL-C, the ranking analysis on TG showed that oat bran had the highest possibility to be the best intervention strategy, followed by oat (Fig. 3 and Supplemental Table 49). However, the results of network meta-analysis suggested that of oat bran and oat did not significantly affect the concentration of TG compared with control. In addition, oat bran exerted greater decreased effects on TG when compared with wheat bran (−0.11 mmol/L, 95% CI: −0.21, −0.01 mmol/L; Supplemental Table 48).

**Fig. 4:**
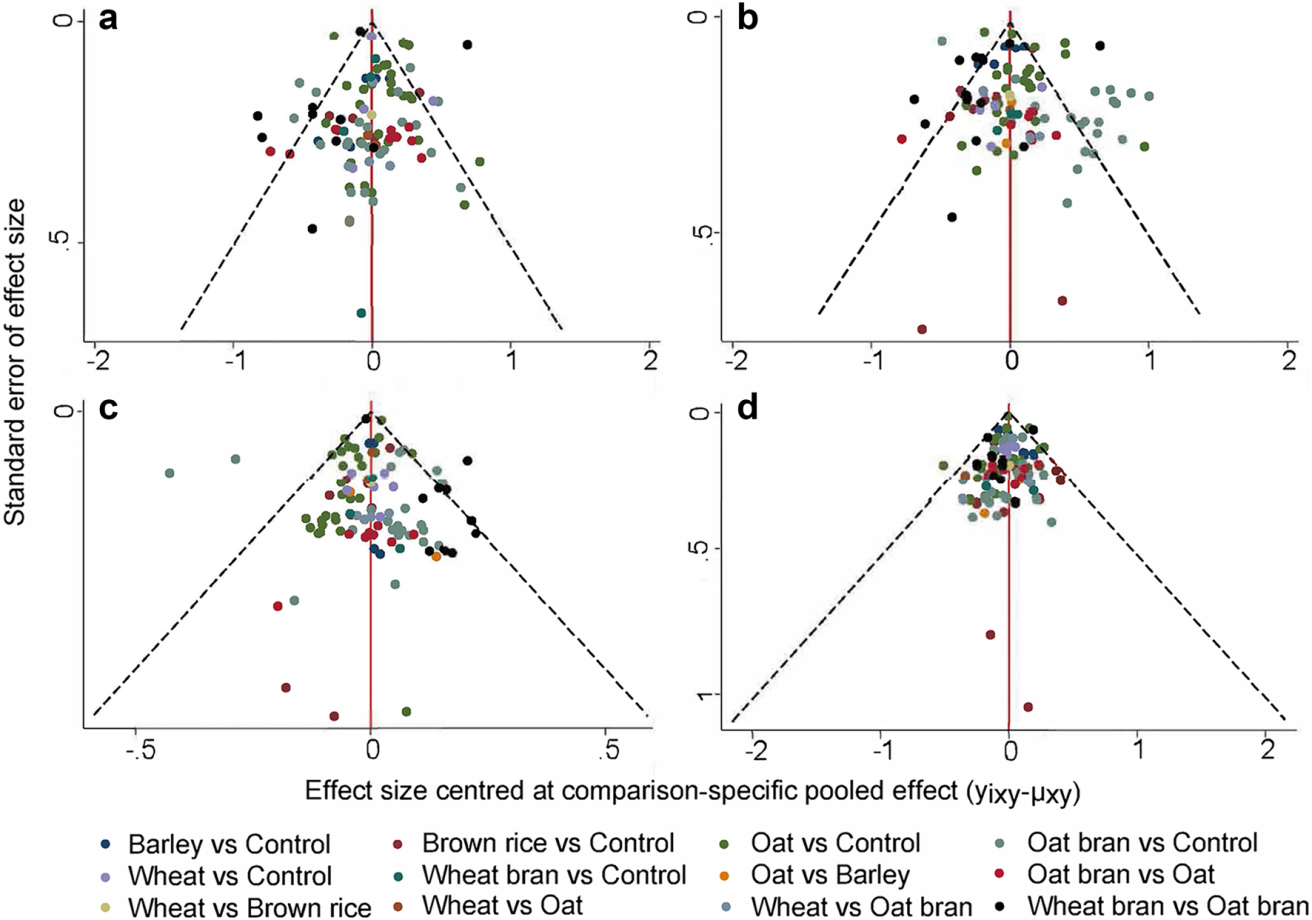
Comparison-adjusted funnel plots for TC (**a**), LDL-C (**b**), HDL-C (**c**) and TG (**d**). The red line represents the null hypothesis that the study-specific effect sizes do not differ from the respective comparison-specific pooled effect estimates. The two black dashed lines represent a 95% CI for the difference between study-specific effect sizes and comparison-specific summary estimates. Different colors correspond to different comparisons

Contributions of direct evidence to the network analysis are reported in Supplemental Tables 4, 18, 32 and 46. The analysis of design-by-treatment model did not identify any significant global inconsistency for TC (*τ*^2^ = 0.017, *P* = 0.81), LDL-C (*τ*^2^ = 0.018, *P* = 0.85), HDL-C (*τ*^2^ = 0.005, *P* = 0.17) and TG (*τ*^2^ = 0.005, *P* = 0.99). Overall, we did not observed any of inconsistencies between evidence derived from direct and indirect comparisons using the node-splitting method. For details of the assessments of inconsistency see Supplemental Tables 5, 19, 33 and 47. Loop-specific analyses indicated that treatment effects estimated from direct and indirect evidence in general did not show significant statistical inconsistencies. Details of the assessments of inconsistency for TC, LDL-C, HDL-C and TG are shown in Supplemental Figs. 4, 6, 8 and 10. The above results of test for inconsistency supported the assumption of transitivity in this network meta-analysis. Finally, the comparison-adjusted funnel plots of the network meta-analysis for TC, LDL-C, HDL-C and TG did not show any significant publication bias (Fig. 4a–d).

The sensitivity analyses using inconsistency model showed similar results with main analyses. In addition, the sensitivity analyses only including the participants with dyslipidemia showed that oat bran was more effective than oat on the reduction of LDL-C. Moreover, the overall outcomes for all variables were not significantly changed when we only including the studies with a parallel design or removing the cluster crossover design trials. Finally, sensitivity analyses based on treatment duration and risk of bias of trials were similar with the main results. Detailed results of the sensitivity analyses are reported in the Supplemental Tables 8–15, 22–29, 36–43 and 50–57.

## Discussion

To our knowledge, this is the first network meta-analysis investigating the comparative effects of different whole grains and brans on blood lipid. A particular strength of network meta-analysis is that one can obtain the relative effect estimations among different interventions by incorporating direct and indirect evidence [14, 15]. In this network meta-analysis, we found that oat bran might be the most effective intervention strategy for TC and LDL-C reductions, while oat ranked the second based on cumulative ranking analysis. Although estimates of effects size varied among different interventions, oat bran and oat were more or no less effective compared with any other types whole grains and brans. Conversely, barley, brown rice, wheat, and wheat bran showed insignificant effects on blood lipid. As suggested by these findings, increasing consumption of oat sources of whole grain might be necessary to dietary adjustment for lipid control.

Evidence about comparative effects of different whole grains and brans on the control of blood lipid is still limited. In this study, we found that oat bran was ranked as the best intervention strategy for TC and LDL-C regulations, and oat was ranked as the second position. Additionally, we demonstrated that oat bran and oat had more or potential favorable effects on lipid control compared with other interventions. The advantage of oat bran and oat in lipid control might be attributed to their high proportion of β-glucan and the mechanism underlying may involve the following reasons: first, β-glucan can act as a physical barrier by inhibiting the absorption of bile acids which is associated with the synthesis and dissociation of cholesterol [35]; second, β-glucan can decrease the enterohepatic circulation of bile acids and increase the excretion of fecal bile acids by binding with bile acids in small intestine [36, 37]; third, β-glucan can lower insulin concentrations through suppressing the absorption of carbohydrate and in turn decrease the synthesis of cholesterol [38]. In addition, β-glucan can improve the blood lipid by modifying the composition of gut microbiota such as increasing the microbial diversity, abundance of the genus Bacteroidaceae, and the ratio of Bacteroidetes/Firmicutes [39–41].

Although we did not find significant differences between oat bran and oat on the control of blood lipid, the sensitivity analysis based on the studies that only included dyslipidemic participants showed that oat bran was more effective than oat on the reduction of LDL-C. The possibility might be that bran is the major and direct source of fiber, minerals, magnesium and phytonutrients [12, 42], which are the primary nutrients responsible for health benefits in whole grains, but with low calorie. Thus, increasing the quantity of whole grain brans alone might provide enough nutrients for health benefits, but without excessive calories intake [43].

This network meta-analysis also has some limitations. First, to address the independent effect of the different whole grains and brans on blood lipid, the studies used mixed whole grains and brans were excluded. The network meta-analysis found that intervention with barley, brown rice, wheat, and wheat bran alone could not markedly improve the blood lipid which might be due to the limited number of head-to-head trials. Although these results are consistent with previous traditional meta-analysis [11], more related high-quality trials with large-scale and well-controlled design are needed to provide clearer answers and more evidence. Second, our network meta-analysis suggested TC and LDL-C were significantly decreased after oat bran and oat interventions. However, it is difficult to evaluate the association of the reductions of TC and LDL-C found in this study in terms of CVDs risk reduction because most of the study durations were less than 1 year and data for relative risk evaluation were not available in the included studies. Third, evidence in this network meta-analysis largely originated from East Asia, North America, and Europe, with fewer from regions such as Southeast Asia, South America, and Africa. Thus, to better understand the effects of whole grains and brans on lipids control, further studies from these regions are required. Fourth, most studies included in our network meta-analysis were placebo controlled trials, the number of head-to-head trials which comparing different active treatments directly is still limited. Future direct comparison trials are needed to further evaluate and confirm our findings. Furthermore, publication bias and heterogeneity are the inevitable problems in the meta-analysis. However, the Egger’s tests and comparison-adjusted funnel plots suggested that no significant publication bias exists in this study. In addition, the results of sensitivity analyses based on study design, intervention duration, baseline lipid level and risk of bias were consistent to the main results. Moreover, the control interventions, as the common comparator, across the included studies were similar and the results of test for inconsistency suggested the assumption of transitivity was valid.

Overall, the present network meta-analysis provided comprehensive evidence about the comparative effects of different whole grains and brans on the control of blood lipid. Both oat bran and oat showed significant lowering effects on TC and LDL-C. In addition, oat bran might be the most optimal strategy for the control of TC and LDL-C, while oat was ranked as the second. The findings suggest that increasing oat sources of whole grain may be beneficial for lipid management.

## Electronic supplementary material

Below is the link to the electronic supplementary material.

Supplementary material 1 (PDF 801 KB)
