# Supplementary material for: Comparative effects of different whole grains and brans on blood lipid: a network meta-analysis
Source: Eur J Nutr. 2018 Sep 22;58(7):2779–87. doi: 10.1007/s00394-018-1827-6 (PMC6769090; doi:10.1007/s00394-018-1827-6)
Supplement: Supplementary file 1 — Supplementary material 1 (PDF 801 KB) [file 394_2018_1827_MOESM1_ESM.pdf]

SUPPLEMENTARY MATERIALS

Journal name: European Journal of Nutrition

Title: Comparative effects of different whole grains and brans on blood lipid: a network meta-analysis

Author Names: Suocheng Hui[1], Kai Liu[1,2], Hedong Lang[1], Yang Liu[1], Xiaolan Wang[1], Xiaohui Zhu[1],Steve Doucette[3], Long Yi[1], Mantian Mi[1]

Suocheng Hui and Kai Liu contributed equally to this work.

Address correspondence to M-TM, Research Center for Nutrition and Food Safety, Institute of Military Preventive Medicine, the Third Military Medical University, Chongqing Key Laboratory of Nutrition and Food Safety, Chongqing Medical Nutrition Research Center, Chongqing 400038, P. R. China. Tel: +86 2368752305, Fax: +86 2368752305, E-mail address: mi_mt2009@hotmail.com.

Comparative effects of different whole grains and brans on blood lipid: a study protocol for network meta-analysis

BACKGROUND

Cardiovascular diseases (CVDs) were estimated to the cause of 17.3 million deaths every year, accounting for nearly 50% of global deaths, which have brought heavy burden to the public health globally [1-3]. Abnormalities of lipid metabolism are important risk factors of CVDs, recent studies have indicated that 1% decrease in total cholesterol (TC) and LDL cholesterol (LDL-C) has been shown to reduce risk of CVD by 3% and 1% respectively [4].

Dietary therapy is showing great bright future in improving lipid status [5]. Whole grain diet is frequently encouraged for the prevention and treatment of CVDs [6]. Accumulating evidence has suggested that whole grains and bran consumption are inversely associated with lipid profile [7]. However, the results of clinical trials investigating the effects of whole grains on the blood lipids in humans are still inconsistent [8].

Whole grain is defined to contain bran, germ, and endosperm, and these three parts are still present in their natural proportions [9]. During the grain-refining process, the bran and germ of whole grain is removed from the starchy endosperm and the latter is ground into flour. At the same time, fiber, minerals, vitamins and other phytochemicals of mainly contained in whole grain bran are removed during milling processes [9]. In addition, results of large prospective cohort studies have indicated the significant association between whole grains especially their subcomponent brans and risk of cardiovascular diseases [7, 10]. However, there is no individual RCT investigating the comparative effects of different whole grains and brans on blood lipid.

To adequately assess the comparative effectiveness of different whole grains and brans on the control

of blood lipid, we will conduct a frequentist model network meta-analysis, which will treat every kind of whole grains and bran as separate treatment and incorporates both direct and indirect comparisons of treatment strategies while synthesizing all available data [11, 12]. Besides, our network meta-analysis will rank the relative effects of different whole grains and bran on the control of blood lipid.

OBJECTIVES

To estimate and rank the comparative effects of different whole grains and brans on the control of blood lipid.

METHODS

Criteria for considering studies for this review

Types of studies

Any clinical trials evaluating the comparative effects of different whole grains and brans on lipids in healthy or high-risk of CVDs population will be included.

Types of participants

Apparently healthy or high-risk CVDs population (subjects with known dyslipidemia, hyperglycemia, hypertension, overweight or obesity, or a combination of these factors) and not diagnosed with CVDs will be considered eligible for inclusion.

Types of interventions

Participants receive the treatment products of barley, brown rice, oat, oat bran, rye, rye bran, wheat, wheat bran alone or refined-grain diets or products and the effects of whole grains and brans could be distinguished. Intervention duration lasted more than 2 weeks.

Search methods for identification of studies

Electronic searches

The structured search strategies will use the text key words *whole grain, grain, wholegrain, whole meal, whole wheat, wheat, rice, maize, oat, barley, corn, rye, millet, sorghum, triticale, canary seed, amaranth, buckwheat, quinoa,* which will be paired with the following words: *lipid profile, lipid distribution, blood lipid, cholesterol, total cholesterol, TC, low density lipoprotein, LDL, LDL-C, high density lipoprotein, HDL, HDL-C, triglyceride, triacylglycerol, triglyceride, TG, TAG, lipoprotein.* The search will be restricted to the reports of clinical trials conducted in human subjects. Literature search will be executed in three databases:

1. PubMed (http://www.ncbi.nlm.nih.gov/pubmed/);
2. Embase (http://www.embase.com/search/advanced/);
3. The Cochrane Library (http://www.cochrane.org/).

Selection of studies

The search strategy described will be used to obtain titles and abstracts of studies that may be relevant to the review. Two authors will independently complete the screening of the titles and abstracts of articles retrieved. After primary screening, the full text of articles retained from screening will then be obtained and those that meet the eligible criteria will be included.

Data extraction and management

Outcome data

Two independent reviewers will extract from each included study for change scores or baseline and post-intervention values of TC, LDL-C, HDL cholesterol (HDL-C), and triglycerides (TG). For crossover design trials, we will extract the data of two phases. In parallel design studies, all treatment outcomes at different visits will be extracted and used to estimate the intervention effects. Data will be cross checked between authors and any discrepancies will be resolved by discussion with a third author.

Data on study characteristics

The following characteristics will be extracted from each study:

1) Information of authors, publication year, region, sample size, study design and funding source;

2) Treatment duration, treatment products, and dietary fiber content of intervention products;

3) Population information on age and body mass index (BMI).

Assessment of risk of bias in included studies

The risk of bias will be assessed using the Cochrane Risk of Bias assessment tool. The assessment items included adequacy of sequence generation, allocation concealment, blinding, blinding of outcome assessments, incomplete outcome data, selective reporting, and other biases [13]. Two researchers will review the studies and judge the risk of bias independently. And any discrepancies will be resolved by consensus and arbitration by a third investigator.

Assessment of quality of evidence

The quality of evidence will be assessed using the Grading of Recommendations Assessment, Development and Evaluation (GRADE) framework, which characterizes the evidence on the basis of

the study limitations, imprecision, inconsistency, indirectness, and publication bias [14].

Data synthesis

Methods for direct treatment comparisons

First, pairwise meta-analyses will be conducted by synthesizing studies that compared interventions head-to-head using a random-effects model to estimate the treatment effects [15]. We will calculate I^2 statistic and P value to identify the heterogeneity among the included studies [16]. In addition, we will use the Egger's test to detect the small-study effects. The pairwise meta-analysis will be conducted using meta package for R software.

Methods for indirect and mixed comparisons

Frequentist model network meta-analyses will be conducted to estimate the comparative effects of different whole grains and brans on the control of blood lipid if at least five treatments arms are available across the studies [17].

We will perform meta-analysis in STATA (StataCorp. 2011. Stata Statistical Software: Release 14. College Station, TX) using the mvmeta command [16] and Stata routines described elsewhere [11], which are available at http://www.mtm.uoi.gr/index.php/stata-routines-for-network-meta-analysis.

Measures of treatment effect

Relative treatment effects

The outcomes of treatments for pairwise and network meta-analysis will be estimated using mean differences (MDs).

Relative treatment ranking

We will estimate the ranking probabilities for all treatments of being at each possible rank for each intervention and each outcome. A treatment hierarchy will be obtained by the surface under the cumulative ranking curve (SUCRA) and mean ranks. SUCRA can also be expressed as a percentage interpreted as the percentage of efficacy of a treatment that would be ranked first without uncertainty [11].

Assessment of transitivity across treatment comparisons

The fundamental premise underlying network meta-analysis is transitivity [12, 18, 19]. In this study, we will evaluate whether the transitivity assumption is valid by assessing the inconsistency between direct and indirect evidence.

Assessment of statistical heterogeneity

Local approaches for evaluating inconsistency

The presence of local inconsistency will be evaluated using the loop-specific approach [20, 21], and the results of this approach will be presented graphically in a forest plot using the ifplot command in STATA.14.0 [11]. To infer whether the inconsistency factor is incompatible with zero, we will look at the 95% confidence interval and a loop-specific z-test.

Global approaches for evaluating inconsistency

We will use the design-by-treatment interaction model to check the assumption of consistency in the entire network. Using this approach we will infer about the presence of inconsistency from any source in the entire network based on a chi-square test. The design-by-treatment interaction model will be performed in STATA using the mvmeta command [22].

Sensitivity analysis

To assess the robustness of our findings, we will perform sensitivity analysis by fitting the inconsistency model. In addition, other sensitivity analysis will be conducted according to baseline lipid level, study design, duration of follow-up and risk of bias.

Other analysis

We will use the comparison-adjusted funnel plot to visually assess the evidence for publication bias in the network [11].

REFERENCE

1. New initiative launched to tackle cardiovascular disease, the world's number one killer. Version current 22 September, 2016. Internet: http://www.who.int/cardiovascular_diseases/en/. (accessed 2016 June 17th).
2. (2015) Global, regional, and national age-sex specific all-cause and cause-specific mortality for 240 causes of death, 1990-2013: a systematic analysis for the Global Burden of Disease Study 2013. Lancet (London, England) 385:117-171. doi 10.1016/s0140-6736(14)61682-2.
3. Smith SC, Jr., Collins A, Ferrari R, Holmes DR, Jr., Logstrup S, McGhie DV, Ralston J, Sacco RL, Stam H, Taubert K, Wood DA, Zoghbi WA (2012) Our time: a call to save preventable death from cardiovascular disease (heart disease and stroke). J Am Coll Cardiol 60:2343-2348. doi 10.1016/j.jacc.2012.08.962.
4. (2002) Third Report of the National Cholesterol Education Program (NCEP) Expert Panel on Detection, Evaluation, and Treatment of High Blood Cholesterol in Adults (Adult Treatment Panel III) final report. Circulation 106:3143-3421.
5. Stroes ES, Thompson PD, Corsini A, Vladutiu GD, Raal FJ, Ray KK, Roden M, Stein E, Tokgozoglu L, Nordestgaard BG, Bruckert E, De Backer G, Krauss RM, Laufs U, Santos RD, Hegele RA, Hovingh GK, Leiter LA, Mach F, Marz W, Newman CB, Wiklund O, Jacobson TA, Catapano AL, Chapman MJ, Ginsberg HN (2015) Statin-associated muscle symptoms: impact on statin therapy-European Atherosclerosis Society Consensus Panel Statement on Assessment, Aetiology and Management. Eur Heart J 36:1012-1022. doi 10.1093/eurheartj/ehv043.
6. Chen GC, Tong X, Xu JY, Han SF, Wan ZX, Qin JB, Qin LQ (2016) Whole-grain intake and total, cardiovascular, and cancer mortality: a systematic review and meta-analysis of prospective studies. The American journal of clinical nutrition 104:164-172. doi 10.3945/ajcn.115.122432.
7. Zong G, Gao A, Hu FB, Sun Q (2016) Whole Grain Intake and Mortality From All Causes, Cardiovascular Disease, and Cancer: A Meta-Analysis of Prospective Cohort Studies. Circulation 133:2370-2380. doi 10.1161/circulationaha.115.021101.
8. Hollaender PL, Ross AB (2015) Whole-grain and blood lipid changes in apparently healthy adults: a systematic review and meta-analysis of randomized controlled studies. 102:556-572. doi 10.3945/ajcn.115.109165.
9. Slavin JL, Martini MC, Jacobs DR, Jr., Marquart L (1999) Plausible mechanisms for the protectiveness of whole grains. The American journal of clinical nutrition 70:459s-463s.
10. Aune D, Keum N, Giovannucci E, Fadnes LT, Boffetta P, Greenwood DC, Tonstad S, Vatten LJ, Riboli E, Norat T (2016) Whole grain consumption and risk of cardiovascular disease, cancer, and all cause and cause specific mortality: systematic review and dose-response meta-analysis of prospective studies. BMJ (Clinical research ed.) 353:i2716. doi 10.1136/bmj.i2716.
11. Chaimani A, Higgins JP, Mavridis D, Spyridonos P, Salanti G (2013) Graphical tools for network meta-analysis in STATA. PloS one 8:e76654. doi 10.1371/journal.pone.0076654.
12. Salanti G (2012) Indirect and mixed-treatment comparison, network, or multiple-treatments meta-analysis: many names, many benefits, many concerns for the next generation evidence synthesis tool. Research synthesis methods 3:80-97. doi 10.1002/jrsm.1037.
13. Higgins J, Green S, editors. Chapter 8: Assessing risk of bias in included studies. Cochrane Handbook for Systematic Reviews of Interventions. The Cochrane Collaboration. Version 5.1.0 (updated March 2011); 2011
14. Salanti G, Del Giovane C, Chaimani A, Caldwell DM, Higgins JP (2014) Evaluating the quality of

evidence from a network meta-analysis. PloS one 9:e99682. doi 10.1371/journal.pone.0099682.
15. DerSimonian R, Laird N (1986) Meta-analysis in clinical trials. Control Clin Trials 7:177-188.
16. Higgins JP, Thompson SG, Deeks JJ, Altman DG (2003) Measuring inconsistency in meta-analyses. BMJ (Clinical research ed.) 327:557-560. doi 10.1136/bmj.327.7414.557.
17. Tricco AC, Ashoor HM, Antony J, Beyene J, Veroniki AA, Isaranuwatchai W, Harrington A, Wilson C, Tsouros S, Soobiah C, Yu CH, Hutton B, Hoch JS, Hemmelgarn BR, Moher D, Majumdar SR, Straus SE (2014) Safety, effectiveness, and cost effectiveness of long acting versus intermediate acting insulin for patients with type 1 diabetes: systematic review and network meta-analysis. BMJ (Clinical research ed.) 349:g5459. doi 10.1136/bmj.g5459.
18. Cipriani A, Higgins JP, Geddes JR, Salanti G (2013) Conceptual and technical challenges in network meta-analysis. Ann Intern Med 159:130-137. doi 10.7326/0003-4819-159-2-201307160-00008.
19. Jansen JP, Naci H (2013) Is network meta-analysis as valid as standard pairwise meta-analysis? It all depends on the distribution of effect modifiers. BMC medicine 11:159. doi 10.1186/1741-7015-11-159.
20. Bucher HC, Guyatt GH, Griffith LE, Walter SD (1997) The results of direct and indirect treatment comparisons in meta-analysis of randomized controlled trials. Journal of clinical epidemiology 50:683-691.
21. Salanti G, Marinho V, Higgins JP (2009) A case study of multiple-treatments meta-analysis demonstrates that covariates should be considered. Journal of clinical epidemiology 62:857-864. doi 10.1016/j.jclinepi.2008.10.001.
22. White IR, Barrett JK, Jackson D, Higgins JP (2012) Consistency and inconsistency in network meta-analysis: model estimation using multivariate meta-regression. Research synthesis methods 3:111-125. doi 10.1002/jrsm.1045.

Supplemental materials and methods

Literature search

Full search strategy for Pubmed: (((((((((((((((((((lipid profile) OR lipid distribution) OR blood lipid) OR cholesterol) OR total cholesterol) OR TC) OR low density lipoprotein) OR LDL) OR LDL-C) OR high density lipoprotein) OR HDL) OR HDL-C) OR triglyceride) OR triacylglycerol) OR triglyceride) OR TG) OR TAG) OR lipoprotein)) AND (((((((((((((((((((((whole grain) OR grain) OR wholegrain) OR whole meal) OR whole wheat) OR wheat) OR rice) OR maize) OR oat) OR barley) OR corn) OR rye) OR millet) OR sorghum) OR triticale) OR canary seed) OR amaranth) OR buckwheat) OR quinoa)) AND "humans"[MeSH Terms].

Supplemental Table 1 Characteristics of eligible studies[a]

Author, publication year, country (reference)	No. of subjects	Age[b] (years)	BMI[b] (kg/m²)	Study design	Duration (weeks)	Intervention products	Dietary fiber content of intervention products (g/day)
Beck, 2010, Australia [1]	56	37.4 (5.8)	29.3 (2.2)	Parallel	12	Oat bran ready-to-eat cereal, muesli bars and snack packs	Oat bran: 7.0, 18.9
Berg, 2003, Germany [2]	152	53.2 (6.5)	30.3 (1.9)	Parallel	4	Oat bran bread, rolls, sauces and desserts	Oat bran: NR
Bremer, 1991, New Zealand [3]	12	53.0 (10.0)	NR	Crossover	4	Oat bran bread; Wheat bran bread	Oat bran: 12.9; Wheat bran: 15.3
Chang, 2013, Taiwan [4]	34	38.5 (11.2)	29.4 (2.4)	Parallel	12	Oat cereal supplements	Oat: NR
Charlton, 2012, Australia [5]	90	51.3 (10.3)	27.3 (4.3)	Parallel	6	Oat porridge and oat-based cereal bars	Oat: 8.6, 4.3
Chen, 2006, United States [6]	102	47.9 (8.4)	28.9 (4.6)	Parallel	12	Oat bran concentrated muffin	Oat bran: 15.9
Davidson, 1991, United States [7]	140	52.7 (NR)	25.4 (NR)	Parallel	12	Oatmeal and oat bran supplements	Oat: 2.3, 4.5, 8.2; Oat bran: 4.1, 6.8, 12.3
Davy, 2002, United States [8]	36	59.0 (8.4)	29.4 (3.4)	Parallel	12	Oat bran cold cereal; Whole wheat hot cereal	Oat: 14.0; Wheat: 14.0
Gerhardt, 1998, United States [9]	30	51.7 (1.5)	24.4 (NR)	Parallel	6	Oat bran products	Oat bran: 8.3
Giacco, 2009, Italy [10]	15	54.5 (7.6)	27.4 (3.0)	Crossover	3	Whole wheat bread, pasta, rusks, and crackers	Wheat: 22.0
Gold, 1988, United States [11]	72	25.7 (3.2)	NR	Parallel	4	Oat bran muffins; Whole wheat muffins	Oat bran: 10.0; Wheat: 11.0
Hajifaraji, 2012, Iran [12]	39	52.6 (6.6)	26.7 (4.6)	Parallel	3	Barley bread; Oat bread	Barley: 9.0; Oat: 10.0

Supplemental Table 1 Continued

Author, publication year, country (reference)	No. of subjects	Age[b] (years)	BMI[b] (kg/m²)	Study design	Duration (weeks)	Intervention products	Dietary fiber content of intervention products (g/day)
Jenkins, 1993, Canada [13]	67	56.6 (12.0)	25.6 (2.9)	Parallel	2	Wheat bran supplements; Oat bran supplements	Oat bran: 25.0; Wheat bran: 25.0
Jenkins, 2002, Canada [14]	23	63.0 (4.8)	26.7 (5.3)	Crossover	12	Wheat bran cereal and bread	Wheat bran: 19.0
Johnston, 1998, United States [15]	124	57.0 (NR)	NR	Parallel	6	Ready-to-eat oat cereal	Oat: 9.0
Kabir, 2002, Switzerland [16]	13	59.0 (7.2)	28.0 (3.6)	Crossover	4	Oat bran concentrated bread; Whole wheat bread	Oat: 8.7; Wheat: 10.0
Karmally, 2005, United States [17]	152	49.0 (10.7)	29.2 (3.9)	Parallel	6	Oat bran cereal	Oat bran: NR
Kashian, 1991, Canada [18]	84	55.8 (13.0)	25.7 (2.9)	Parallel	2	Oat-bran cereals; Wheat-bran cereals	Oat bran: 25.0; Wheat bran: 24.0
Kazemzadeh, 2014, Iran [19]	35	32.6 (6.0)	29.8 (4.0)	Crossover	6	Brown rice supplements	Brown rice: 5.3
Kestin, 1990, Australia [20]	24	46.0 (10.0)	25.4 (2.0)	Crossover	4	Bread and muffin of oat bran or wheat bran	Oat bran: 11.8; Wheat bran: 12.5
Kristensen, 2011, Denmark [21]	12	NR	24.9 (2.9)	Crossover	2	Oat bran breads	Oat bran: 10.4
Kristensen, 2012, Italy [22]	72	59.7 (5.5)	30.2 (3.1)	Parallel	12	Whole wheat bread, pasta, and biscuits	Wheat: 11.0
Lee, 2016, Korea [23]	93	57.9 (7.4)	23.5 (3.0)	Parallel	4	Brown rice supplements	Brown rice: NR
Leinonen, 2000, Finland [24]	39	43.0 (8.0)	24.5 (3.0)	Crossover	4	Rye bread	Rye: 19.2
Li, 2003, Japan [25]	10	20.4 (1.3)	19.2 (2.0)	Crossover	4	Barley food supplements	Barley: 13.9
Li, 2015, China [26]	238	59.5 (6.1)	26.8 (2.5)	Parallel	4	Whole grain oats cereals	Oat: 4.4, 8.7
Liatis, 2009, Greece [27]	41	63.0 (9.0)	28.5 (4.3)	Parallel	3	Oat bread	Oat: >21.1
Liebman,1983, United States [28]	20	NR	NR	Crossover	6	Wheat bran bread	Wheat bran: 31.0

Supplemental Table 1 Continued

Author, publication year, country (reference)	No. of subjects	Age^b (years)	BMI^b (kg/m²)	Study design	Duration (weeks)	Intervention products	Dietary fiber content of intervention products (g/day)
Lovegrove, 1999, United States [29]	62	56.6 (9.3)	25.9 (3.5)	Parallel	12	Oat bran cereal; Wheat bran cereal	Oat bran: 5.9; Wheat bran: 0.5
McIntosh, 1991, Australia [30]	21	44.2 (7.6)	25.6 (2.7)	Crossover	4	Barley cereal; Whole wheat cereal	Barley: 25.3; Wheat: 26.0
Moazzami, 2012, Sweden [31]	33	58.8 (5.8)	27.2 (3.2)	Crossover	8	Rye bran bread	Rye bran: 36.9
Pick, 1996, Canada [32]	8	46.0 (2.8)	27.6 (0.6)	Crossover	12	Oat bran concentrated bread	Oat bran: 18.0
Pins, 2002, United States [33]	88	47.6 (16.2)	30.9 (4.9)	Parallel	12	Oatmeal and oat squares	Oat :11.7
Poulter, 1993, United Kingdom [34]	58	56.4 (2.5)	NR	Crossover	4	Oat bran crispies	Oat bran: >4.5
Robitaille, 2004, Canada [35]	34	38.3 (7.8)	29.1 (4.6)	Parallel	4	Oat bran-enriched muffins	Oat bran: 6.2
Romero, 1998, Mexico [36]	46	34.1 (9.0)	26.9 (3.0)	Parallel	8	Oat bran cookies; Wheat bran cookies	Oat bran: 9.6; Wheat bran: 1.9
Saltzman, 2001, United States [37]	43	44.6 (22.0)	26.4 (3.3)	Parallel	6	Quick oats as hot cereal, bread and casseroles	Oat: 8.7
Shimabukuro, 2013, Japan [38]	27	NR	26.7 (3.7)	Crossover	8	Brown rice supplements	Brown rice: NR
Stevens,1985, United States [39]	25	54.4 (3.0)	NR	Parallel	6	Oat bran muffins	Oat bran: 13.2
Swain, 1990, United States [40]	40	30.0 (NR)	NR	Crossover	6	Oat bran supplements	Oat bran: 28.8
Tarpila, 1978, Finland [41]	22	51.4 (9.3)	NR	Parallel	52	Wheat bran cereal	Wheat bran: NR
Thongoun, 2013, Thailand [42]	24	51.0 (6.9)	26.8 (5.8)	Crossover	8	Oatmeal for breakfast	Oat: 7.4
Tighe, 2010, United Kingdom [43]	206	51.7 (6.6)	28.0 (4.2)	Parallel	12	Whole wheat bread and cereals	Wheat: 18.5
Torronen, 1992, Finland [44]	28	41.0 (NR)	NR	Parallel	8	Oat bran concentrate bread; Whole wheat bread	Oat bran: NR; Wheat: NR

Supplemental Table 1 Continued

Author, publication year, country (reference)	No. of subjects	Age[b] (years)	BMI[b] (kg/m²)	Study design	Duration (weeks)	Intervention products	Dietary fiber content of intervention products (g/day)
Uusitupa, 1992, Finland [45]	36	47.8 (7.9)	26.5 (3.0)	Parallel	8	Oat bran supplements; Wheat bran supplements	Oat bran: 29.8; Wheat bran: 20.5
Van Horn, 1988, United States [46]	236	42.4 (NR)	NR	Parallel	8	Oat meal supplements	Oat: 5.9
Van Horn, 1991, United States [47]	80	42.5 (12.8)	26.2 (3.6)	Parallel	8	Oat meal supplements	Oat: 5.6
Vitaglione, 2015, Italy [48]	68	38.6 (11.7)	29.8 (2.7)	Parallel	8	Whole wheat products	Wheat: 8.0
Wang, 2013, United States [49]	57	52.5 (9.0)	25.7 (2.7)	Parallel	12	Brown rice	Brown rice: NR
Wang, 2016, Canada [50]	30	59.0 (2.0)	28.5 (1.2)	Crossover	5	Barley cereal	Barley: NR
Winblad, 1995, Finland [51]	59	44.0 (6.2)	26.7 (3.0)	Crossover	6	Oat bran as rolls, flakes, small cakes	Oat bran: 15.1
Wolever, 2010, Canada [52]	367	52.2 (9.1)	27.5 (4.2)	Parallel	4	Oat bran cereals; Wheat bran cereals	Oat bran: 5.6, 6.0, 7.9, 8.1; Wheat bran 5.9
Zhang, 1992, Sweden [53]	9	49.0 (NR)	NR	Crossover	3	Oat bran bread	Oat bran: 29.0
Zhang, 2011, China [54]	202	49.7 (6.9)	25.7 (3.1)	Parallel	16	Brown rice supplements	Brown rice: 5.6
Zhang, 2012, China [55]	166	53.2 (6.5)	25.5 (3.0)	Parallel	6	Oat meal supplements	Oat: 10.6

[a]NR, Not report

[b]Age and BMI are provided as mean (SD)

Supplemental Table 2 Funding sources of the included studies

Author, publication year, country (reference)	Funding source
Beck, 2010, Australia [1]	Australian Research Council Linkage between the University of Wollongong and Cereal Partners Worldwide (Grant ID LP0561586)
Berg, 2003, Germany [2]	Not report
Bremer, 1991, New Zealand [3]	Not report
Chang, 2013, Taiwan [4]	STANDARD Foods Co.
Charlton, 2012, Australia [5]	Cereal Partners Worldwide Ltd.
Chen, 2006, United States [6]	The Quaker Oats Co.
Davidson, 1991, United States [7]	Quaker Oats Co.
Davy, 2002, United States [8]	Quaker Oats Co.
Gerhardt, 1998, United States [9]	Pacific Rice Products and Sutter Heart Institute, CA
Giacco, 2009, Italy [10]	R&D Barilla G&R, Italy
Gold, 1988, United States [11]	National Heart, Lung, and Blood Institute Preventive Cardiology Academic Award to Dr Davidson (HL-01243)
Hajifaraji, 2012, Iran [12]	National Nutrition and Food Technology Research Institute
Jenkins, 1993, Canada [13]	The grand from National Institutes of Health the Ontario Ministry of Health, and the Natural Sciences and Engineering Research Council of Canada (ROI HL 39689)
Jenkins, 2002, Canada [14]	University-Industry Research Partnership Program of the Natural Sciences and Engineering Research Council of Canada; Loblaw Brands Limited and Kraft Canada, Don Mills, Ontario, Canada
Johnston, 1998, United States [15]	General Mills. Inc
Kabir, 2002, Switzerland [16]	The grand from National Institute of Health and Medical Research, Pierre and Marie Curie University, and Nestle Center, Orbe, Switzerland
Karmally, 2005, United States [17]	General Mills. Inc, Minneapolis
Kashian, 1991, Canada [18]	The grant Ministry of Health of Ontario
Kazemzadeh, 2014, Iran [19]	The grand from Isfanhan University of Medical Sciences and Food Security Research Center (391213)
Kristensen, 2011, Denmark [21]	The grant of Faculty of Life Sciences, University of Copenhagen

Supplemental Table 2 Continued

Author, publication year, country (reference)	Funding source
Kristensen, 2012, Italy [22]	European Commission in the Communities 6th Framework Programme, Project HEALTHGRAIN (FOOD-CT-2005-514008), and the University of Copenhagen, Faculty of Life Sciences and LMC FOOD research school
Lee, 2016, Korea [23]	Ministry of Health & Welfare (A111716-1202-0000100), the Korean Health Technology R&D Project, and the Ministry of Health and Welfare, Republic of Korea (HI13C0715 and HI11C1300)
Leinonen, 2000, Finland [24]	Fazer Bakeries Ltd., Vaasan & Vaasan Ltd. and the Technology Development Centre of Finland
Li, 2003, Japan [25]	Haku-Baku Company Ltd. (Yamanashi,Japan)
Li, 2015, China [26]	Inner Mongolia Sanzhuliang Natural Oats Industry Corporation (IMSNOIC)
Liatis, 2009, Greece [27]	Not report
Liebman,1983, United States [28]	Not report
Lovegrove, 1999, United States [29]	Nestle.
McIntosh, 1991, Australia [30]	Barley Research Council of Australia
Moazzami, 2012, Sweden [31]	Fazer Bakeries Ltd., Vaasan & Vaasan Oy, the Technology Development Center of Finland
Pick, 1996, Canada [32]	Quaker Oats Company of Canada
Pins, 2002, United States [33]	Quaker Oats Company, Barrington, Illinois
Poulter, 1993, United Kingdom [34]	Quaker Oats, Southall, UK
Robitaille, 2004, Canada [35]	The grant from Réseau de santé cardiovasculaire du Fonds de la recherche en santé du Québec' (RSCVFRSQ)
Romero, 1998, Mexico [36]	Not report
Saltzman, 2001, United States [37]	Quaker Oats Company, National Institutes of Health Grant (AG12829) and U.S. Department of agriculture contract (53-3K06-5-10)
Shimabukuro, 2013, Japan [38]	The grants of Ministry of Education, Culture, Sports, Science and Technology and the Ministry of Health, Labour and Welfare, Japan
Stevens,1985, United States [39]	The grand from University of Virginia Diabetes Research and Training Center, the University of Virginia School of Medicine Research and Development Committee

Supplemental Table 2 Continued

Author, publication year, country (reference)	Funding source
Swain, 1990, United States [40]	Grand (from the National Heart,Lung and Blood Institute HL34593) and a Clinical Research Center grant (M01-RR02635)
Tarpila, 1978, Finland [41]	Grants from the Finnish State Council for Medical Research
Thongoun, 2013, Thailand [42]	Not report
Tighe, 2010, United Kingdom [43]	Grant from the Food Standard Agency (NO2035) and funding from the Scottish Government. Paterson Arran Ltd., Livingston, United Kingdom
Torronen, 1992, Finland [44]	Grant from the Olvi Foundation, Finland
Uusitupa, 1992, Finland [45]	Alko Ltd., Finland, the Medical Council of the Academy of Finland, Sigfrid Juselius Foundation, and by a personal grant to Dr. Uusitupa from the Medical Council of Academy of Finland
Van Horn, 1988, United States [46]	Not report
Van Horn, 1991, United States [47]	Quaker Oats Company
Vitaglione, 2015, Italy [48]	General Mills Bell Institute of Health and Nutrition with an unconditional grant
Wang, 2013, United States [49]	Not report
Wang, 2016, Canada [50]	Agriculture and Agri-Food Canada (AAFC)s Growing Forward program
Winblad, 1995, Finland [51]	Melia Ltd.
Wolever, 2010, Canada [52]	CreaNutrition, The Swedish Governmental Agency for Innovations Systems, and Agriculture and Agri-Food Canada
Zhang, 1992, Sweden [53]	Swedish Board of Technical Development (STU), the Swedish Council of Forestry and Agricultural Research (SJFR), and Wasabrod AB
Zhang, 2011, China [54]	Chief Scientist Program of Shanghai Institutes for Biological Sciences, Chinese Academy of Sciences (SIBS2008006), Chinese Academy of Sciences (KSCX1-YW-02 and KSCX2-EW-R-10), National Basic Research Program of China (2011CB504002), and National Natural Science Foundation of China (30930081 and 81021002)
Zhang, 2012, China [55]	Xin Tan Health Research Development center (20080101) and Pepsi Co china Foods

REFERENCES:

1. Beck EJ, Tapsell LC, Batterham MJ, Tosh SM, Huang XF (2010) Oat beta-glucan supplementation does not enhance the effectiveness of an energy-restricted diet in overweight women. The British journal of nutrition 103:1212-1222. doi 10.1017/s0007114509992856.
2. Berg A, Konig D, Deibert P, Grathwohl D, Berg A, Baumstark MW, Franz IW (2003) Effect of an oat bran enriched diet on the atherogenic lipid profile in patients with an increased coronary heart disease risk. A controlled randomized lifestyle intervention study. Ann Nutr Metab 47:306-311. doi 72404.
3. Bremer JM, Scott RS, Lintott CJ (1991) Oat bran and cholesterol reduction: evidence against specific effect. Aust N Z J Med 21:422-426.
4. Chang HC, Huang CN, Yeh DM, Wang SJ, Peng CH, Wang CJ (2013) Oat prevents obesity and abdominal fat distribution, and improves liver function in humans. Plant Foods Hum Nutr 68:18-23. doi 10.1007/s11130-013-0336-2.
5. Charlton KE, Tapsell LC, Batterham MJ, O'Shea J, Thorne R, Beck E, Tosh SM (2012) Effect of 6 weeks' consumption of beta-glucan-rich oat products on cholesterol levels in mildly hypercholesterolaemic overweight adults. The British journal of nutrition 107:1037-1047. doi 10.1017/s0007114511003850.
6. Chen J, He J, Wildman RP, Reynolds K, Streiffer RH, Whelton PK (2006) A randomized controlled trial of dietary fiber intake on serum lipids. European journal of clinical nutrition 60:62-68. doi 10.1038/sj.ejcn.1602268.
7. Davidson MH, Dugan LD, Burns JH, Bova J, Story K, Drennan KB (1991) The hypocholesterolemic effects of beta-glucan in oatmeal and oat bran. A dose-controlled study. Jama 265:1833-1839.
8. Davy BM, Davy KP, Ho RC, Beske SD, Davrath LR, Melby CL (2002) High-fiber oat cereal compared with wheat cereal consumption favorably alters LDL-cholesterol subclass and particle numbers in middle-aged and older men. The American journal of clinical nutrition 76:351-358.
9. Gerhardt AL, Gallo NB (1998) Full-fat rice bran and oat bran similarly reduce hypercholesterolemia in humans. The Journal of nutrition 128:865-869.
10. Giacco R, Clemente G, Cipriano D, Luongo D, Viscovo D, Patti L, Di Marino L, Giacco A, Naviglio D, Bianchi MA, Ciati R, Brighenti F, Rivellese AA, Riccardi G (2010) Effects of the regular consumption of wholemeal wheat foods on cardiovascular risk factors in healthy people. Nutr Metab Cardiovasc Dis 20:186-194. doi 10.1016/j.numecd.2009.03.025.
11. Gold KV, Davidson DM (1988) Oat bran as a cholesterol-reducing dietary adjunct in a young, healthy population. The Western journal of medicine 148:299-302.
12. Hajifaraji M, Najjar Safari S, Rezvani V, Rashidkhani B, Maddah M (2012) Comparison study between the effect of oat and barley breads on serum glucose and lipid profiles in dyslipidemic and type 2 diabetic subjects: A short-term trial. Mediterranean Journal of Nutrition and Metabolism 5:247-252. doi 10.1007/s12349-012-0092-0.
13. Jenkins DJ, Hegele RA, Jenkins AL, Connelly PW, Hallak K, Bracci P, Kashtan H, Corey P, Pintilia M, Stern H, et al. (1993) The apolipoprotein E gene and the serum low-density lipoprotein cholesterol response to dietary fiber. Metabolism 42:585-593.
14. Jenkins DJ, Kendall CW, Augustin LS, Martini MC, Axelsen M, Faulkner D, Vidgen E, Parker T, Lau H, Connelly PW, Teitel J, Singer W, Vandenbroucke AC, Leiter LA, Josse RG (2002) Effect of wheat bran on glycemic control and risk factors for cardiovascular disease in type 2 diabetes.

Diabetes care 25:1522-1528.
15. Johnston L, Reynolds HB, Hunninghake DB, Schultz K, Westereng B (1998) Cholesterol-lowering benefits of a whole grain oat ready to eat cereal. In: Nutrition Clinical Care. p 6-12
16. Kabir M, Oppert JM, Vidal H, Bruzzo F, Fiquet C, Wursch P, Slama G, Rizkalla SW (2002) Four-week low-glycemic index breakfast with a modest amount of soluble fibers in type 2 diabetic men. Metabolism 51:819-826.
17. Karmally W, Montez MG, Palmas W, Martinez W, Branstetter A, Ramakrishnan R, Holleran SF, Haffner SM, Ginsberg HN (2005) Cholesterol-lowering benefits of oat-containing cereal in Hispanic americans. J Am Diet Assoc 105:967-970. doi 10.1016/j.jada.2005.03.006.
18. Kashtan H, Stern HS, Jenkins DJ, Jenkins AL, Hay K, Marcon N, Minkin S, Bruce WR (1992) Wheat-bran and oat-bran supplements' effects on blood lipids and lipoproteins. The American journal of clinical nutrition 55:976-980.
19. Kazemzadeh M, Safavi SM, Nematollahi S, Nourieh Z (2014) Effect of Brown Rice Consumption on Inflammatory Marker and Cardiovascular Risk Factors among Overweight and Obese Non-menopausal Female Adults. Int J Prev Med 5:478-488.
20. Kestin M, Moss R, Clifton PM, Nestel PJ (1990) Comparative effects of three cereal brans on plasma lipids, blood pressure, and glucose metabolism in mildly hypercholesterolemic men. The American journal of clinical nutrition 52:661-666.
21. Kristensen M, Bugel S (2011) A diet rich in oat bran improves blood lipids and hemostatic factors, and reduces apparent energy digestibility in young healthy volunteers. European journal of clinical nutrition 65:1053-1058. doi 10.1038/ejcn.2011.102.
22. Kristensen M, Toubro S, Jensen MG, Ross AB, Riboldi G, Petronio M, Bugel S, Tetens I, Astrup A (2012) Whole grain compared with refined wheat decreases the percentage of body fat following a 12-week, energy-restricted dietary intervention in postmenopausal women. The Journal of nutrition 142:710-716. doi 10.3945/jn.111.142315.
23. Lee YM, Kim SA, Lee IK, Kim JG, Park KG, Jeong JY, Jeon JH, Shin JY, Lee DH (2016) Effect of a Brown Rice Based Vegan Diet and Conventional Diabetic Diet on Glycemic Control of Patients with Type 2 Diabetes: A 12-Week Randomized Clinical Trial. PloS one 11:e0155918. doi 10.1371/journal.pone.0155918.
24. Leinonen KS, Poutanen KS, Mykkanen HM (2000) Rye bread decreases serum total and LDL cholesterol in men with moderately elevated serum cholesterol. The Journal of nutrition 130:164-170.
25. Li J, Kaneko T, Qin LQ, Wang J, Wang Y (2003) Effects of barley intake on glucose tolerance, lipid metabolism, and bowel function in women. Nutrition (Burbank, Los Angeles County, Calif.) 19:926-929.
26. Li X, Cai X, Ma X, Jing L, Gu J, Bao L, Li J, Xu M, Zhang Z, Li Y (2016) Short- and Long-Term Effects of Wholegrain Oat Intake on Weight Management and Glucolipid Metabolism in Overweight Type-2 Diabetics: A Randomized Control Trial. Nutrients 8. doi 10.3390/nu8090549.
27. Liatis S, Tsapogas P, Chala E, Dimosthenopoulos C, Kyriakopoulos K, Kapantais E, Katsilambros N (2009) The consumption of bread enriched with betaglucan reduces LDL-cholesterol and improves insulin resistance in patients with type 2 diabetes. Diabetes Metab 35:115-120. doi 10.1016/j.diabet.2008.09.004.
28. Liebman M, Smith MC, Iverson J, Thye FW, Hinkle DE, Herbert WG, Ritchey SJ, Driskell JA

(1983) Effect of coarse wheat bran fiber and exercise on plasma lipids and lipoproteins in moderately overweight men. The American journal of clinical nutrition 37:71-81.

29. Lovegrove JA, Clohessy A, Milon H, Williams CM (2000) Modest doses of beta-glucan do not reduce concentrations of potentially atherogenic lipoproteins. The American journal of clinical nutrition 72:49-55.
30. McIntosh GH, Whyte J, McArthur R, Nestel PJ (1991) Barley and wheat foods: influence on plasma cholesterol concentrations in hypercholesterolemic men. The American journal of clinical nutrition 53:1205-1209.
31. Moazzami AA, Bondia-Pons I, Hanhineva K, Juntunen K, Antl N, Poutanen K, Mykkanen H (2012) Metabolomics reveals the metabolic shifts following an intervention with rye bread in postmenopausal women--a randomized control trial. Nutrition journal 11:88. doi 10.1186/1475-2891-11-88.
32. Pick ME, Hawrysh ZJ, Gee MI, Toth E, Garg ML, Hardin RT (1996) Oat bran concentrate bread products improve long-term control of diabetes: a pilot study. J Am Diet Assoc 96:1254-1261. doi 10.1016/s0002-8223(96)00329-x.
33. Pins JJ, Geleva D, Keenan JM, Frazel C, O'Connor PJ, Cherney LM (2002) Do whole-grain oat cereals reduce the need for antihypertensive medications and improve blood pressure control? The Journal of family practice 51:353-359.
34. Poulter N, Chang CL, Cuff A, Poulter C, Sever P, Thom S (1994) Lipid profiles after the daily consumption of an oat-based cereal: a controlled crossover trial. The American journal of clinical nutrition 59:66-69.
35. Robitaille J, Fontaine-Bisson B, Couture P, Tchernof A, Vohl MC (2005) Effect of an oat bran-rich supplement on the metabolic profile of overweight premenopausal women. Ann Nutr Metab 49:141-148. doi 10.1159/000086149.
36. Romero AL, Romero JE, Galaviz S, Fernandez ML (1998) Cookies enriched with psyllium or oat bran lower plasma LDL cholesterol in normal and hypercholesterolemic men from Northern Mexico. Journal of the American College of Nutrition 17:601-608.
37. Saltzman E, Das SK, Lichtenstein AH, Dallal GE, Corrales A, Schaefer EJ, Greenberg AS, Roberts SB (2001) An oat-containing hypocaloric diet reduces systolic blood pressure and improves lipid profile beyond effects of weight loss in men and women. The Journal of nutrition 131:1465-1470.
38. Shimabukuro M, Higa M, Kinjo R, Yamakawa K, Tanaka H, Kozuka C, Yabiku K, Taira S, Sata M, Masuzaki H (2014) Effects of the brown rice diet on visceral obesity and endothelial function: the BRAVO study. The British journal of nutrition 111:310-320. doi 10.1017/s0007114513002432.
39. Stevens J, Burgess MB, Kaiser DL, Sheppa CM (1985) Outpatient management of diabetes mellitus with patient education to increase dietary carbohydrate and fiber. Diabetes care 8:359-366.
40. Swain JF, Rouse IL, Curley CB, Sacks FM (1990) Comparison of the effects of oat bran and low-fiber wheat on serum lipoprotein levels and blood pressure. The New England journal of medicine 322:147-152. doi 10.1056/nejm199001183220302.
41. Tarpila S, Miettinen TA, Metsaranta L (1978) Effects of bran on serum cholesterol, faecal mass, fat, bile acids and neutral sterols, and biliary lipids in patients with diverticular disease of the colon. Gut 19:137-145.

42. Thongoun P, Pavadhgul P, Bumrungpert A, Satitvipawee P, Harjani Y, Kurilich A (2013) Effect of oat consumption on lipid profiles in hypercholesterolemic adults. Journal of the Medical Association of Thailand = Chotmaihet thangphaet 96 Suppl 5:S25-32.
43. Tighe P, Duthie G, Vaughan N, Brittenden J, Simpson WG, Duthie S, Mutch W, Wahle K, Horgan G, Thies F (2010) Effect of increased consumption of whole-grain foods on blood pressure and other cardiovascular risk markers in healthy middle-aged persons: a randomized controlled trial. The American journal of clinical nutrition 92:733-740. doi 10.3945/ajcn.2010.29417.
44. Torronen R, Kansanen L, Uusitupa M, Hanninen O, Myllymaki O, Harkonen H, Malkki Y (1992) Effects of an oat bran concentrate on serum lipids in free-living men with mild to moderate hypercholesterolaemia. European journal of clinical nutrition 46:621-627.
45. Uusitupa MI, Ruuskanen E, Makinen E, Laitinen J, Toskala E, Kervinen K, Kesaniemi YA (1992) A controlled study on the effect of beta-glucan-rich oat bran on serum lipids in hypercholesterolemic subjects: relation to apolipoprotein E phenotype. Journal of the American College of Nutrition 11:651-659.
46. Van Horn L, Emidy LA, Liu KA, Liao YL, Ballew C, King J, Stamler J (1988) Serum lipid response to a fat-modified, oatmeal-enhanced diet. Prev Med 17:377-386.
47. Van Horn L, Moag-Stahlberg A, Liu KA, Ballew C, Ruth K, Hughes R, Stamler J (1991) Effects on serum lipids of adding instant oats to usual American diets. Am J Public Health 81:183-188.
48. Vitaglione P, Mennella I, Ferracane R, Rivellese AA, Giacco R, Ercolini D, Gibbons SM, La Storia A, Gilbert JA, Jonnalagadda S, Thielecke F, Gallo MA, Scalfi L, Fogliano V (2015) Whole-grain wheat consumption reduces inflammation in a randomized controlled trial on overweight and obese subjects with unhealthy dietary and lifestyle behaviors: role of polyphenols bound to cereal dietary fiber. The American journal of clinical nutrition 101:251-261. doi 10.3945/ajcn.114.088120.
49. Wang B, Medapalli R, Xu J, Cai W, Chen X, He JC, Uribarri J (2013) Effects of a whole rice diet on metabolic parameters and inflammatory markers in prediabetes. e-SPEN Journal 8:e15-e20.
50. Wang Y, Harding SV, Eck P, Thandapilly SJ, Gamel TH, Abdel-Aal el SM, Crow GH, Tosh SM, Jones PJ, Ames NP (2016) High-Molecular-Weight beta-Glucan Decreases Serum Cholesterol Differentially Based on the CYP7A1 rs3808607 Polymorphism in Mildly Hypercholesterolemic Adults. The Journal of nutrition 146:720-727. doi 10.3945/jn.115.223206.
51. Winblad I, Joensuu T, Korpela H (1995) Effect of oat bran supplemented diet on hypercholesterolaemia. Scand J Prim Health Care 13:118-121.
52. Wolever TM, Tosh SM, Gibbs AL, Brand-Miller J, Duncan AM, Hart V, Lamarche B, Thomson BA, Duss R, Wood PJ (2010) Physicochemical properties of oat beta-glucan influence its ability to reduce serum LDL cholesterol in humans: a randomized clinical trial. The American journal of clinical nutrition 92:723-732. doi 10.3945/ajcn.2010.29174.
53. Zhang JX, Hallmans G, Andersson H, Bosaeus I, Aman P, Tidehag P, Stenling R, Lundin E, Dahlgren S (1992) Effect of oat bran on plasma cholesterol and bile acid excretion in nine subjects with ileostomies. The American journal of clinical nutrition 56:99-105.
54. Zhang G, Pan A, Zong G, Yu Z, Wu H, Chen X, Tang L, Feng Y, Zhou H, Chen X, Li H, Hong B, Malik VS, Willett WC, Spiegelman D, Hu FB, Lin X (2011) Substituting white rice with brown rice for 16 weeks does not substantially affect metabolic risk factors in middle-aged Chinese men and women with diabetes or a high risk for diabetes. The Journal of nutrition 141:1685-1690. doi

10.3945/jn.111.142224.
55. Zhang J, Li L, Song P, Wang C, Man Q, Meng L, Cai J, Kurilich A (2012) Randomized controlled trial of oatmeal consumption versus noodle consumption on blood lipids of urban Chinese adults with hypercholesterolemia. Nutrition journal 11:54. doi 10.1186/1475-2891-11-54.

Supplemental Fig. 1 Risk of bias graph. Judgements of review authors (Low, Unclear and High) for each risk of bias item according to the Cochrane Risk of Bias assessment tool. In this tool, studies were deemed to be at high, low or unclear risk of bias based on adequacy of random sequence generation, allocation concealment, blinding of personnel, blinding, incomplete outcome data, selective reporting, and other bias

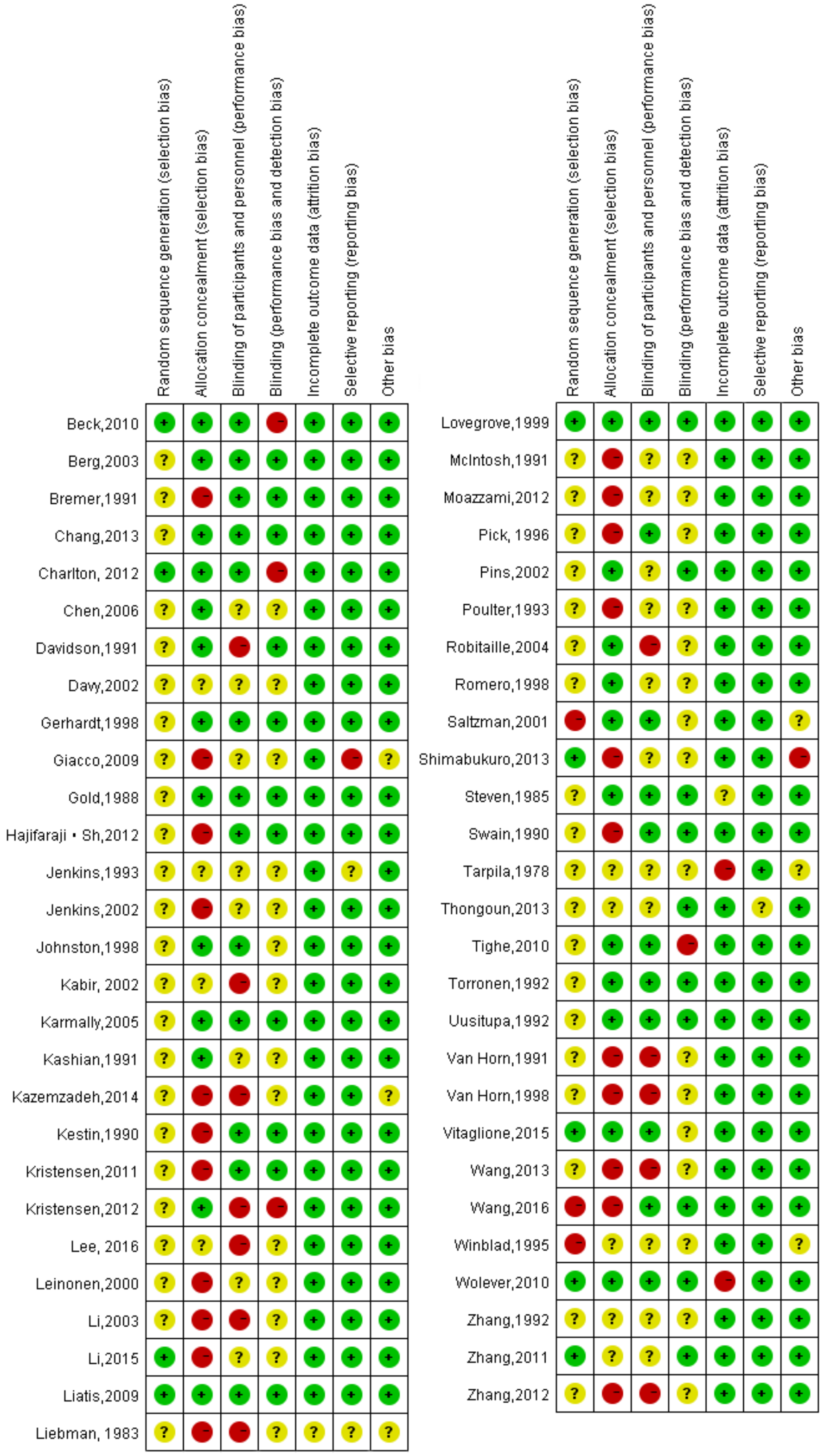

Supplemental Fig. 2 Summary of Cochrane risk of bias results. Judgements of review authors (Low, Unclear and High) for each risk of bias item shown as percentages across all included studies

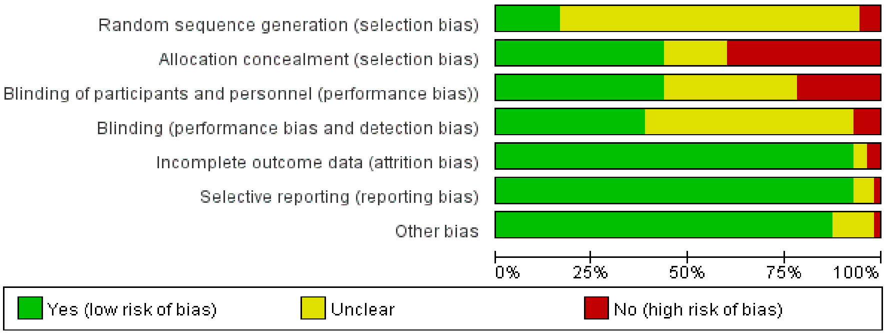

Supplemental Fig. 3 Pairwise meta-analysis of total cholesterol. The square represents the overall estimated effects, and the results were obtained from a random-effects model. MD lower than 0 indicate that the former treatment is more efficacious than latter. MD, mean differences

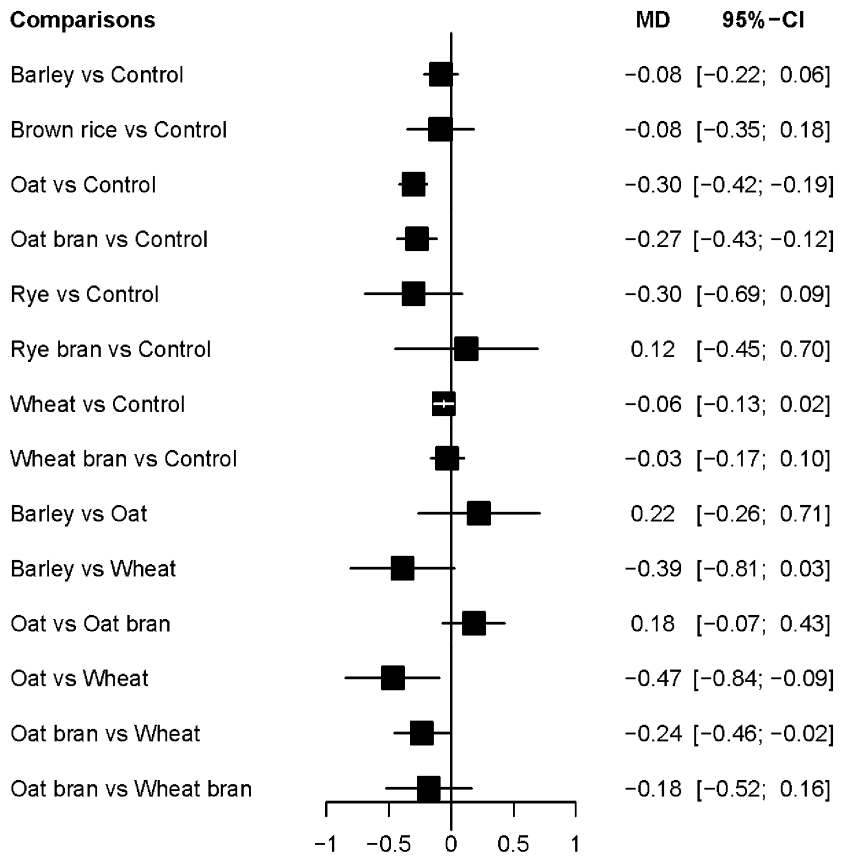

Supplemental Table 3 Pairwise meta-analysis results for total cholesterol

Comparisons	No. of comparisons	Mean differences (95% CI)[a]	I^2	τ^2	P^b	P^c (Egger's test)
Barley vs Control	5	-0.08 (-0.22, 0.06)	0%	0.00	0.59	0.11
Brown rice vs Control	5	-0.08 (-0.35, 0.18)	45.7%	0.04	0.12	0.02
Oat vs Control	27	-0.30 (-0.42, -0.19)	77.6%	0.05	<0.01	0.05
Oat bran vs Control	22	-0.29 (-0.43, -0.12)	55.8%	0.08	0.01	0.38
Rye vs Control	2	-0.30 (-0.69, 0.09)	0%	0.00	0.59	-
Rye bran vs Control	1	0.12 (-0.45, 0.70)	-	-	-	-
Wheat vs Control	6	-0.06 (-0.13, 0.02)	58.9%	0.00	0.03	0.14
Wheat bran vs Control	5	-0.03 (-0.17, 0.10)	0%	0.00	0.96	0.21
Barley vs Oat	2	0.22 (-0.26, 0.71)	0%	0.00	0.73	-
Barley vs Wheat	1	-0.39 (-0.81, 0.03)	-	-	-	-
Oat vs Oat bran	9	0.18 (-0.07, 0.43)	48.4%	0.08	0.06	0.42
Oat vs Wheat	2	-0.47 (-0.84, -0.09)	0%	0.00	0.82	-
Oat bran vs Wheat	4	-0.24 (-0.46, -0.02)	0%	0.00	0.81	-
Oat bran vs Wheat bran	10	-0.18 (-0.52, 0.16)	91.4%	0.25	<0.01	<0.01

[a]Mean differences lower than 0 indicate that the former treatment is more efficacious than latter

[b]*P* for the heterogeneity of pairwise meta-analysis. Heterogeneity was assessed by using Cochran's test, and $P < 0.05$ was considered to indicate significant heterogeneity across studies

[c]*P* for Egger's test. $P < 0.05$ was considered to indicate a significant small-study effects

Supplemental Table 4 Contribution of direct evidence to the network for total cholesterol

Comparisons	No. of comparisons	Contribution to the network (%)
Control vs Barley	5	13.4
Control vs Brown rice	5	15.3
Control vs Oat	27	13.4
Control vs Oat bran	22	8.9
Control vs Wheat	6	15.9
Control vs Wheat bran	5	14.4
Barley vs Oat	2	1.7
Barley vs Wheat	1	1.8
Oat vs Oat bran	9	4.9
Oat vs Wheat	2	1.6
Oat bran vs Wheat	4	5.2
Oat bran vs Wheat bran	10	3.5

Supplemental Fig. 4 Evaluation of inconsistency by using loop-specific approach for total cholesterol. IFs are calculated as the absolute difference between direct and indirect estimates and therefore confidence intervals are truncated to 0. Loops that their lower CI limit does not reach the 0 line are considered to present statistically significant inconsistency. IF, inconsistency factor

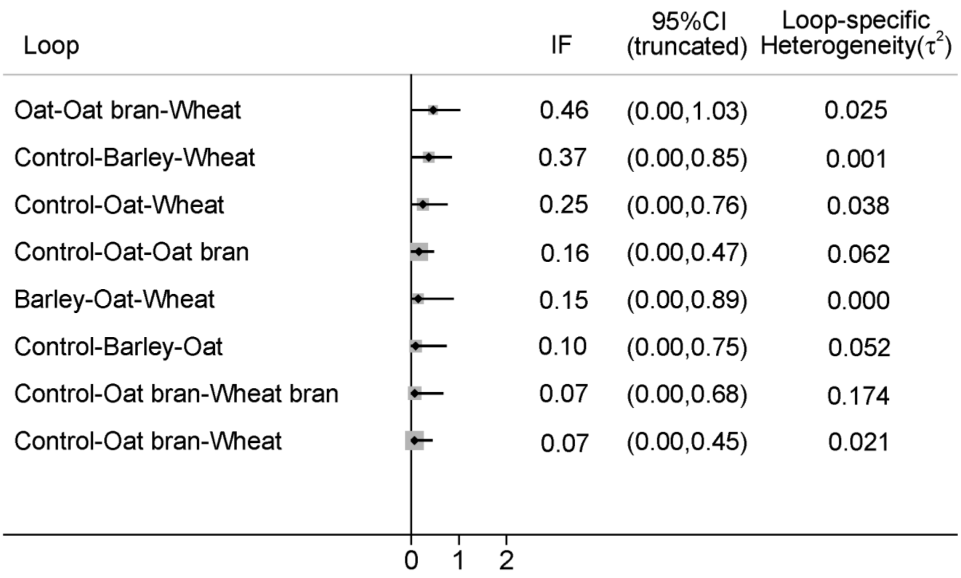

Supplemental Table 5 Node-splitting method for assessment of inconsistency for total cholesterol

Comparisons	Direct		Indirect		Difference		P^a	tau
	Coefficient	SE	Coefficient	SE	Coefficient	SE		
Control vs Barley	-0.1221095	0.1317494	-0.2086694	0.2250723	0.0865598	0.260961	0.74	0.2307815
Control vs Brown rice	-	-	-	-	-	-	-	-
Control vs Oat	-0.2604815	0.0573244	-0.2490505	0.1658578	-0.011431	0.17676	0.95	0.2313763
Control vs Oat bran	-0.3529375	0.0732203	-0.3488937	0.1200637	-0.0040438	0.142009	0.98	0.2317695
Control vs Wheat	-0.0446401	0.1160423	0.0372778	0.1393003	-0.0819179	0.1813077	0.65	0.2315616
Control vs Wheat bran	-0.0663888	0.1562917	-0.1078544	0.1198239	0.0414656	0.1966237	0.83	0.2324312
Barley vs Oat	-0.2357118	0.3027424	-0.0915733	0.1334965	-0.1441385	0.3309169	0.66	0.2311404
Barley vs Wheat	0.3899994	0.3141062	0.0734032	0.1512108	0.3165962	0.3486079	0.36	0.2298051
Oat vs Oat bran	-0.2272053	0.1182553	-0.006594	0.0947947	-0.2206114	0.1515735	0.15	0.2298353
Oat vs Wheat	0.4685686	0.2516291	0.2081646	0.1071464	0.260404	0.2734927	0.34	0.2301584
Oat bran vs Wheat	0.230957	0.1754793	0.3911623	0.1188773	-0.1602053	0.2119582	0.45	0.2307415
Oat bran vs Wheat bran	0.2485845	0.1002491	0.2900568	0.1695744	-0.0414722	0.1966236	0.83	0.2324312

[a]*P* for assessment of inconsistency. $P < 0.05$ was considered to indicate a significant inconsistency existed between direct and indirect evidence

Supplemental Table 6 Comparative effects of different whole grains and brans on the control of total cholesterol[a]

	Control	Wheat bran	Wheat	Oat bran	Oat	Brown rice	Barley
Control	-	-0.09 (-0.28,0.09)	-0.01 (-0.19,0.16)	-0.35 (-0.47,-0.23)	-0.26 (-0.36,-0.15)	-0.09 (-0.37,0.19)	-0.14 (-0.37,0.08)
Wheat bran		-	0.08 (-0.16,0.32)	-0.26 (-0.43,-0.09)	-0.17 (-0.37,0.04)	0.01 (-0.33,0.34)	-0.05 (-0.34,0.23)
Wheat			-	-0.34 (-0.53,-0.15)	-0.25 (-0.44,-0.06)	-0.08 (-0.40,0.25)	-0.13 (-0.40,0.13)
Oat bran				-	0.09 (-0.05,0.24)	0.27 (-0.04,0.57)	0.21 (-0.04,0.46)
Oat					-	0.17 (-0.13,0.47)	0.12 (-0.12,0.35)
Brown rice						-	-0.06 (-0.41,0.30)
Barley							-

[a]Mean differences with 95% confidence intervals (column vs row). Mean differences lower than 0 indicate that the treatments specified in the row is more efficacious than those in the column

Supplemental Table 7 Detailed ranking results of the comparative effects of different whole grains and brans on the control of total cholesterol. The larger the SUCRA value, the better the treatment. SUCRA, surface under the cumulative ranking curve

Treatments	SUCRA (%)	MeanRank
Control	16.6	6.0
Barley	53.3	3.8
Brown rice	40.6	4.6
Oat	79.3	2.2
Oat bran	96.6	1.2
Wheat	21.6	5.7
Wheat bran	41.9	4.5

Supplemental Table 8 Sensitivity analysis using an inconsistency model for total cholesterol[a]

	Control	Wheat bran	Wheat	Oat bran	Oat	Brown rice	Barley
Control	19.2	0.13 (-0.18,0.45)	-0.28 (-0.40,-0.15)	-0.08 (-0.75,0.58)	0.06 (-0.22,0.35)	-0.09 (-0.37,0.20)	-0.12 (-0.39,0.14)
Wheat bran		36.8	0.02 (-0.37,0.41)	-0.37 (-0.78,0.03)	-0.21 (-0.55,0.13)	-0.02 (-0.44,0.40)	-0.06 (-0.46,0.35)
Wheat			31.2	-0.39 (-0.74,-0.05)	-0.23 (-0.50,0.03)	-0.04 (-0.41,0.32)	-0.08 (-0.43,0.27)
Oat bran				95.8	0.16 (-0.12,0.45)	0.35 (-0.03,0.74)	0.32 (-0.05,0.68)
Oat					78.1	0.19 (-0.12,0.50)	0.15 (-0.14,0.45)
Brown rice						40.8	-0.04 (-0.42,0.35)
Barley							48.1

[a]Mean differences with 95% confidence intervals (row vs column). Mean differences lower than 0 indicate that the treatments specified in the row are more efficacious than those in the column. SUCRA values are given in the diagonal of parenthesis. The larger the SUCRA value, the better the treatment. SUCRA, surface under the cumulative ranking curve

Supplemental Table 9 Sensitivity analysis based on the studies only included dyslipidemic participants for total cholesterol[a]

	Control	Wheat bran	Wheat	Oat bran	Oat	Brown rice	Barley
Control	20.9	-0.17 (-0.43,0.09)	0.02 (-0.22,0.26)	-0.40 (-0.56,-0.25)	-0.27 (-0.40,-0.15)	-0.08 (-0.46,0.30)	-0.09 (-0.37,0.18)
Wheat bran		55.5	0.19 (-0.14,0.52)	-0.23 (-0.44,-0.02)	-0.10 (-0.37,0.17)	0.09 (-0.37,0.55)	0.08 (-0.29,0.45)
Wheat			19.5	-0.42 (-0.68,-0.16)	-0.29 (-0.54,-0.04)	-0.10 (-0.54,0.35)	-0.11 (-0.44,0.22)
Oat bran				97.1	0.13 (-0.04,0.30)	0.33 (-0.08,0.73)	0.31 (0.01,0.62)
Oat					76.3	0.20 (-0.20,0.60)	0.18 (-0.11,0.47)
Brown rice						39.6	-0.01 (-0.48,0.45)
Barley							41.2

[a]Mean differences with 95% confidence intervals (row vs column). Mean differences lower than 0 indicate that the treatments specified in the row are more efficacious than those in the column. SUCRA values are given in the diagonal of parenthesis. The larger the SUCRA value, the better the treatment. SUCRA, surface under the cumulative ranking curve

Supplemental Table 10 Sensitivity analysis based on the studies with parallel design for total cholesterol[a]

	Control	Wheat bran	Wheat	Oat bran	Oat	Brown rice	Barley
Control	31.6	-0.08 (-0.34,0.18)	-0.05 (-0.25,0.15)	-0.39 (-0.54,-0.24)	-0.25 (-0.36,-0.14)	0.30 (-0.27,0.87)	-0.01 (-0.63,0.60)
Wheat bran		47.9	0.03 (-0.27,0.33)	-0.31 (-0.52,-0.10)	-0.17 (-0.44,0.10)	0.38 (-0.25,1.01)	0.07 (-0.59,0.73)
Wheat			43.2	-0.34 (-0.56,-0.12)	-0.20 (-0.42,0.02)	0.35 (-0.25,0.96)	0.04 (-0.60,0.68)
Oat bran				96.8	0.14 (-0.03,0.31)	0.69 (0.10,1.28)	0.37 (-0.25,1.00)
Oat					77.9	0.55 (-0.03,1.13)	0.24 (-0.37,0.84)
Brown rice						11.1	-0.31 (-1.15,0.52)
Barley							41.3

[a]Mean differences with 95% confidence intervals (row vs column). Mean differences lower than 0 indicate that the treatments specified in the row are more efficacious than those in the column. SUCRA values are given in the diagonal of parenthesis. The larger the SUCRA value, the better the treatment. SUCRA, surface under the cumulative ranking curve

Supplemental Table 11 Sensitivity analysis when removing the studies with cluster crossover design for total cholesterol[a]

	Control	Wheat bran	Wheat	Oat bran	Oat	Brown rice	Barley
Control	16.6	-0.11 (-0.34,0.11)	-0.01 (-0.19,0.17)	-0.35 (-0.48,-0.22)	-0.26 (-0.37,-0.15)	-0.09 (-0.37,0.20)	-0.14 (-0.37,0.08)
Wheat bran		45.3	0.10 (-0.17,0.37)	-0.24 (-0.43,-0.05)	-0.15 (-0.38,0.09)	0.03 (-0.33,0.39)	-0.03 (-0.35,0.28)
Wheat			21.1	-0.34 (-0.54,-0.14)	-0.25 (-0.44,-0.05)	-0.08 (-0.41,0.26)	-0.13 (-0.40,0.14)
Oat bran				96.1	0.09 (-0.06,0.25)	0.26 (-0.05,0.58)	0.21 (-0.05,0.46)
Oat					78.4	0.17 (-0.13,0.47)	0.11 (-0.13,0.36)
Brown rice						40.0	-0.06 (-0.42,0.30)
Barley							52.4

[a]Mean differences with 95% confidence intervals (row vs column). Mean differences lower than 0 indicate that the treatments specified in the row are more efficacious than those in the column. SUCRA values are given in the diagonal of parenthesis. The larger the SUCRA value, the better the treatment. SUCRA, surface under the cumulative ranking curve

Supplemental Table 12 Sensitivity analysis when excluding the trials with the duration less than 3 weeks for total cholesterol[a]

	Control	Wheat bran	Wheat	Oat bran	Oat	Brown rice	Barley
Control	19.0	-0.05 (-0.25,0.15)	-0.01 (-0.18,0.16)	-0.36 (-0.48,-0.23)	-0.26 (-0.36,-0.16)	-0.09 (-0.36,0.19)	-0.14 (-0.36,0.08)
Wheat bran		32.9	0.04 (-0.22,0.29)	-0.31 (-0.49,-0.12)	-0.21 (-0.43,0.01)	-0.04 (-0.38,0.30)	-0.09 (-0.39,0.20)
Wheat			24.3	-0.34 (-0.54,-0.15)	-0.25 (-0.44,-0.06)	-0.07 (-0.40,0.25)	-0.13 (-0.40,0.13)
Oat bran				97.1	0.10 (-0.05,0.24)	0.27 (-0.03,0.57)	0.21 (-0.04,0.46)
Oat					79.6	0.17 (-0.12,0.47)	0.12 (-0.12,0.35)
Brown rice						42.0	-0.06 (-0.41,0.30)
Barley							55.1

[a]Mean differences with 95% confidence intervals (row vs column). Mean differences lower than 0 indicate that the treatments specified in the row are more efficacious than those in the column. SUCRA values are given in the diagonal of parenthesis. The larger the SUCRA value, the better the treatment. SUCRA, surface under the cumulative ranking curve

Supplemental Table 13 Sensitivity analysis when excluding the trials with the duration less than 4 weeks for total cholesterol[a]

	Control	Wheat bran	Wheat	Oat bran	Oat	Brown rice	Barley
Control	20.0	-0.05 (-0.24,0.15)	-0.00 (-0.18,0.18)	-0.35 (-0.47,-0.23)	-0.25 (-0.35,-0.14)	-0.09 (-0.36,0.19)	-0.14 (-0.36,0.08)
Wheat bran		33.6	0.05 (-0.21,0.30)	-0.30 (-0.49,-0.12)	-0.20 (-0.42,0.02)	-0.04 (-0.38,0.30)	-0.10 (-0.39,0.20)
Wheat			22.1	-0.35 (-0.54,-0.16)	-0.25 (-0.44,-0.05)	-0.09 (-0.41,0.24)	-0.14 (-0.41,0.13)
Oat bran				97.1	0.10 (-0.04,0.25)	0.26 (-0.04,0.57)	0.21 (-0.04,0.46)
Oat					78.7	0.16 (-0.13,0.46)	0.11 (-0.13,0.34)
Brown rice						42.3	-0.05 (-0.41,0.30)
Barley							56.2

[a]Mean differences with 95% confidence intervals (row vs column). Mean differences lower than 0 indicate that the treatments specified in the row are more efficacious than those in the column. SUCRA values are given in the diagonal of parenthesis. The larger the SUCRA value, the better the treatment. SUCRA, surface under the cumulative ranking curve

Supplemental Table 14 Sensitivity analysis when excluding the trials with intervention duration of 16 weeks and 12 months for total cholesterol[a]

	Control	Wheat bran	Wheat	Oat bran	Oat	Brown rice	Barley
Control	13.6	-0.09 (-0.28,0.10)	-0.01 (-0.18,0.16)	-0.35 (-0.47,-0.23)	-0.26 (-0.36,-0.16)	-0.22 (-0.54,0.10)	-0.14 (-0.36,0.08)
Wheat bran		37.5	0.08 (-0.16,0.32)	-0.26 (-0.43,-0.09)	-0.17 (-0.37,0.04)	-0.13 (-0.50,0.24)	-0.05 (-0.34,0.23)
Wheat			18.3	-0.34 (-0.53,-0.15)	-0.25 (-0.44,-0.06)	-0.21 (-0.57,0.16)	-0.13 (-0.40,0.13)
Oat bran				93.7	0.09 (-0.05,0.24)	0.13 (-0.21,0.48)	0.21 (-0.04,0.46)
Oat					74.6	0.04 (-0.30,0.38)	0.12 (-0.12,0.35)
Brown rice						63.3	0.08 (-0.31,0.47)
Barley							49.0

[a]Mean differences with 95% confidence intervals (row vs column). Mean differences lower than 0 indicate that the treatments specified in the row are more efficacious than those in the column. SUCRA values are given in the diagonal of parenthesis. The larger the SUCRA value, the better the treatment. SUCRA, surface under the cumulative ranking curve

Supplemental Table 15 Sensitivity analysis based on the studies with low risk of bias for total cholesterol[a]

	Control	Wheat bran	Wheat	Oat bran	Oat	Brown rice	Barley
Control	31.5	-0.14 (-0.40,0.12)	-0.01 (-0.25,0.23)	-0.37 (-0.51,-0.22)	-0.27 (-0.40,-0.13)	0.30 (-0.32,0.92)	-0.03 (-0.67,0.61)
Wheat bran		55.6	0.13 (-0.20,0.45)	-0.23 (-0.44,-0.01)	-0.13 (-0.40,0.15)	0.44 (-0.23,1.11)	0.11 (-0.57,0.80)
Wheat			34.9	-0.36 (-0.60,-0.11)	-0.25 (-0.52,0.01)	0.31 (-0.35,0.98)	-0.02 (-0.70,0.66)
Oat bran				94.7	0.10 (-0.07,0.28)	0.67 (0.03,1.30)	0.34 (-0.31,0.99)
Oat					77.2	0.57 (-0.07,1.20)	0.24 (-0.39,0.87)
Brown rice						12.4	-0.33 (-1.22,0.57)
Barley							43.7

[a]Mean differences with 95% confidence intervals (row vs column). Mean differences lower than 0 indicate that the treatments specified in the row are more efficacious than those in the column. SUCRA values are given in the diagonal of parenthesis. The larger the SUCRA value, the better the treatment. SUCRA, surface under the cumulative ranking curve

Supplemental Table 16 Overall GRADE quality of evidence for total cholesterol from network meta-analysis[a]

Comparisons	Study limitations	Imprecision	Heterogeneity and Inconsistency	Indirectness	Publication bias	Confidence in MD for overall change in TC
Barley vs Control	100% of the estimate from studies at high risk, and 0% at moderate risk	MD -0.14, 95% CI (-0.37,0.08)	Mild heterogeneity according to I^2 (0%) and *P* (0.50) in direct comparisons No inconsistency between the direct and indirect estimate (Node-split *P*= 0.74 and *tau*= 0.23)	The treatment effects were not significantly influenced by clinical modifiers in the subgroup analyses	Undetectable by the routine method The comparison-adjusted funnel plot for the network is not suggestive of any dominant publication bias	Very Low (Downgrade by three levels due to study limitations (for two levels), and imprecision)
Brown rice vs Control	60% of the estimate from studies at high risk, and 20% at moderate risk	MD -0.09, 95% CI (-0.37,0.19)	Low heterogeneity according to I^2 (45.7%) and *P* (0.10) in direct comparisons. Only direct comparisons and no node-splitting inconsistency	The treatment effects were not significantly influenced by clinical modifiers in the subgroup analyses	Undetectable by the routine method The comparison-adjusted funnel plot for the network is not suggestive of any dominant publication bias	Low (Downgrade by two levels due to study limitations and imprecision)
Oat vs Control	27.2% of the estimate from studies at high risk, and 27.2% at moderate risk	MD -0.26, 95% CI (-0.36,-0.15)	High heterogeneity according to I^2 (77.6%) and *P* (<0.01) in direct comparisons No inconsistency between the direct and indirect estimate (Node-split *P*= 0.95 and *tau*= 0.23)	The treatment effects were not significantly influenced by clinical modifiers in the subgroup analyses	Undetectable by the routine method The comparison-adjusted funnel plot for the network is not suggestive of any dominant publication bias	Low (Downgrade by two levels due to study limitations and Heterogeneity and Inconsistency)

Supplemental Table 16 Continued

Comparisons	Study limitations	Imprecision	Heterogeneity and Inconsistency	Indirectness	Publication bias	Confidence in MD for overall change in TC
Oat bran vs Control	0% of the estimate from studies at high risk, and 100% at moderate risk	MD -0.35, 95% CI (-0.47,-0.23)	Moderate heterogeneity according to I^2 (55.8%) and *P* (0.01) in direct comparisons No inconsistency between the direct and indirect estimate (Node-split *P*= 0.98 and *tau*= 0.23)	The treatment effects were not significantly influenced by clinical modifiers in the subgroup analyses	Undetectable by the routine method The comparison-adjusted funnel plot for the network is not suggestive of any dominant publication bias	High
Wheat vs Control	50% of the estimate from studies at high risk, and 25% at moderate risk	MD -0.01, 95% CI (-0.19,0.16)	Moderate heterogeneity according to I^2 (58.9%) and *P* (0.21) in direct comparisons No inconsistency between the direct and indirect estimate (Node-split *P*= 0.65 and *tau*= 0.23)	The treatment effects were not significantly influenced by clinical modifiers in the subgroup analyses	Undetectable by the routine method The comparison-adjusted funnel plot for the network is not suggestive of any dominant publication bias	Low (Downgrade by two levels due to study limitations and imprecision)
Wheat bran vs Control	50% of the estimate from studies at high risk, and 50% at moderate risk	MD -0.09, 95% CI (-0.28,0.09)	Mild heterogeneity according to I^2 (0%) and *P* (0.86) in direct comparisons No inconsistency between the direct and indirect estimate (Node-split *P*= 0.83 and *tau*= 0.23)	The treatment effects were not significantly influenced by clinical modifiers in the subgroup analyses	Undetectable by the routine method The comparison-adjusted funnel plot for the network is not suggestive of any dominant publication bias	Low (Downgrade by two levels due to study limitations and imprecision)

Supplemental Table 16 Continued

Comparisons	Study limitations	Imprecision	Heterogeneity and Inconsistency	Indirectness	Publication bias	Confidence in MD for overall change in TC
Barley vs Oat	0% of the estimate from studies at high risk, and 100% at moderate risk	MD 0.12, 95% CI (-0.12,0.35)	Mild heterogeneity according to I^2 (0%) and *P* (0.67) in direct comparisons No inconsistency between the direct and indirect estimate (Node-split P= 0.66 and tau= 0.22)	The treatment effects were not significantly influenced by clinical modifiers in the subgroup analyses	Undetectable by the routine method The comparison-adjusted funnel plot for the network is not suggestive of any dominant publication bias	Moderate (Downgrade by one level due to imprecision)
Barley vs Wheat	0% of the estimate from studies at high risk, and 100% at moderate risk	MD -0.13, 95% CI (-0.40,0.13)	Only one head-to-head study, and no heterogeneity No inconsistency between the direct and indirect estimate (Node-split *P*= 0.36 and *tau*= 0.22)	The treatment effects were not significantly influenced by clinical modifiers in the subgroup analyses	Undetectable by the routine method The comparison-adjusted funnel plot for the network is not suggestive of any dominant publication bias	Moderate (Downgrade by one level due to imprecision)
Oat vs Oat bran	0% of the estimate from studies at high risk, and 100% at moderate risk	MD 0.09, 95% CI (-0.05,0.24)	Low heterogeneity according to I^2 (48.4%) and *P* (0.03) in direct comparisons No inconsistency between the direct and indirect estimate (Node-split *P*= 0.15 and *tau*= 0.23)	The treatment effects were not significantly influenced by clinical modifiers in the subgroup analyses	Undetectable by the routine method The comparison-adjusted funnel plot for the network is not suggestive of any dominant publication bias	Moderate (Downgrade by one level due to imprecision)

Supplemental Table 16 Continued

Comparisons	Study limitations	Imprecision	Heterogeneity and Inconsistency	Indirectness	Publication bias	Confidence in MD for overall change in TC
Oat vs Wheat	0% of the estimate from studies at high risk, and 100% at moderate risk	MD -0.25, 95% CI (-0.44,-0.06)	Mild heterogeneity according to I^2 (0%) and *P* (0.88) in direct comparisons No inconsistency between the direct and indirect estimate (Node-split *P*= 0.34 and *tau*= 0.23)	The treatment effects were significantly influenced by some clinical modifiers in the subgroup analyses	Undetectable by the routine method The comparison-adjusted funnel plot for the network is not suggestive of any dominant publication bias	Moderate (Downgrade by one level due to Indirectness)
Oat bran vs Wheat	0% of the estimate from studies at high risk, and 50% at moderate risk	MD -0.34, 95% CI (-0.53,-0.15)	Mild heterogeneity according to I^2 (0%) and *P* (0.94) in direct comparisons No inconsistency between the direct and indirect estimate (Node-split *P*= 0.45 and *tau*= 0.23)	The treatment effects were not significantly influenced by clinical modifiers in the subgroup analyses	Undetectable by the routine method The comparison-adjusted funnel plot for the network is not suggestive of any dominant publication bias	High
Oat bran vs Wheat bran	0% of the estimate from studies at high risk, and 100% at moderate risk	MD -0.26, 95% CI (-0.43,-0.09)	High heterogeneity according to I^2 (91.4%) and *P* (<0.01) in direct comparisons No inconsistency between the direct and indirect estimate (Node-split *P*= 0.45 and *tau*= 0.23)	The treatment effects were significantly influenced by some clinical modifiers in the subgroup analyses	Undetectable by the routine method The comparison-adjusted funnel plot for the network is not suggestive of any dominant publication bias	Moderate (Downgrade by one level due to Heterogeneity and Inconsistency)

[a]The quality of evidence of network estimates for all outcomes by using the GRADE framework, which characterizes the quality of a body of evidence on the basis of the study limitations, imprecision, inconsistency, indirectness, and publication bias.

Supplemental Fig. 5 Pairwise meta-analysis of LDL cholesterol. The square represents the overall estimated effects, and the results were obtained from a random-effects model. MD lower than 0 indicate that the former treatment is more efficacious than latter. MD, mean differences

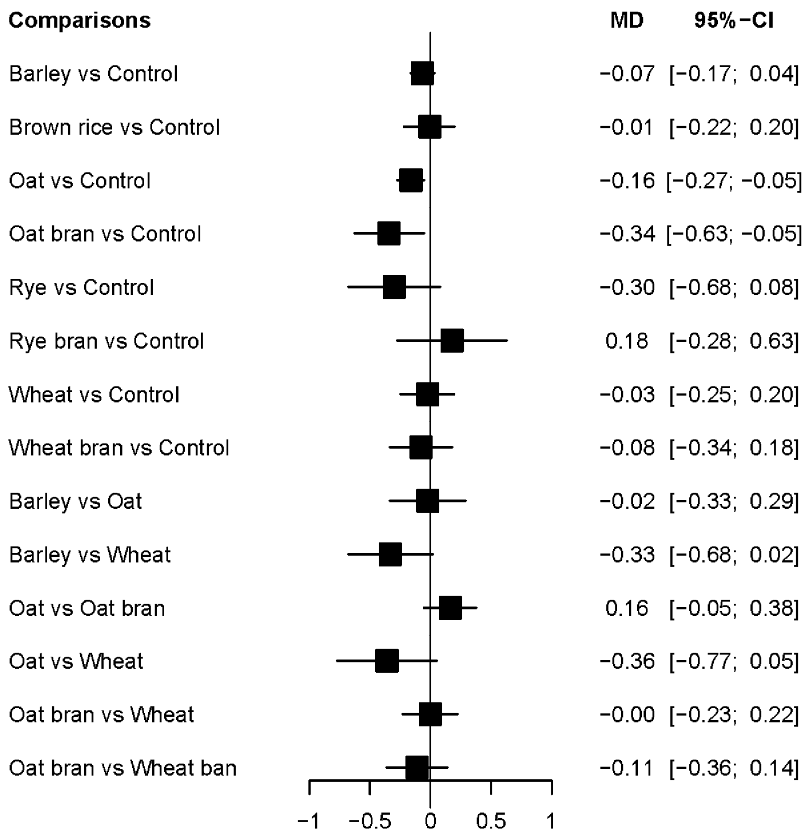

Supplemental Table 17 Pairwise meta-analysis results for LDL cholesterol

Comparisons	No. of comparisons	Mean differences (95% CI)[a]	I^2	τ^2	P^b	P^c (Egger's test)
Barley vs Control	5	-0.07 (-0.17, 0.04)	51.8%	0.00	0.08	0.10
Brown rice vs Control	8	-0.01 (-0.22, 0.20)	64.4%	0.01	0.01	0.02
Oat vs Control	27	-0.16 (-0.27, -0.05)	82.6%	0.06	<0.01	0.03
Oat bran vs Control	20	-0.34 (-0.63, -0.05)	88.2%	0.40	<0.01	0.20
Rye vs Control	2	-0.30 (-0.68, 0.08)	0%	0.00	0.92	-
Rye bran vs Control	1	0.18 (-0.28, 0.63)	-	-	-	-
Wheat vs Control	5	-0.03 (-0.25, 0.20)	17.4%	0.01	0.30	0.14
Wheat bran vs Control	5	-0.08 (-0.34, 0.18)	0%	0.00	0.93	0.27
Barley vs Oat	2	-0.02 (-0.33, 0.29)	0%	0.00	0.92	-
Barley vs Wheat	1	-0.33 (-0.68, 0.02)	-	-	-	-
Oat vs Oat bran	9	0.16 (-0.05, 0.38)	38.3%	0.05	0.11	<0.01
Oat vs Wheat	1	-0.36 (-0.77, 0.05)	-	-	-	-
Oat bran vs Wheat	4	-0.00 (-0.23, 0.22)	0%	0.00	0.54	-
Oat bran vs Wheat bran	14	-0.11 (-0.36, 0.14)	82.9%	0.20	<0.01	0.11

[a]Mean differences lower than 0 indicate that the former treatment is more efficacious than latter

[b]*P* for the heterogeneity of pairwise meta-analysis. Heterogeneity was assessed by using Cochran's test, and $P < 0.05$ was considered to indicate significant heterogeneity across studies

[c]*P* for Egger's test. $P < 0.05$ was considered to indicate a significant small-study effects

Supplemental Table 18 Contribution of direct evidence to the network for LDL cholesterol

Comparisons	No. of comparisons	Contribution to the network (%)
Control vs Barley	5	13.6
Control vs Brown rice	8	13.8
Control vs Oat	27	15.1
Control vs Oat bran	20	4.7
Control vs Wheat	4	7.8
Control vs Wheat bran	5	9.2
Barley vs Oat	2	2.5
Barley vs Wheat	1	3.8
Oat vs Oat bran	9	8.7
Oat vs Wheat	2	2.4
Oat bran vs Wheat	4	8.7
Oat bran vs Wheat bran	14	9.7

Supplemental Fig. 6 Evaluation of inconsistency by using loop-specific approach for LDL cholesterol. IFs along with their confidence intervals are displayed. IFs are calculated as the absolute difference between direct and indirect estimates and therefore confidence intervals are truncated to 0. Loops that their lower CI limit does not reach the 0 line are considered to present statistically significant inconsistency. IF, inconsistency factor

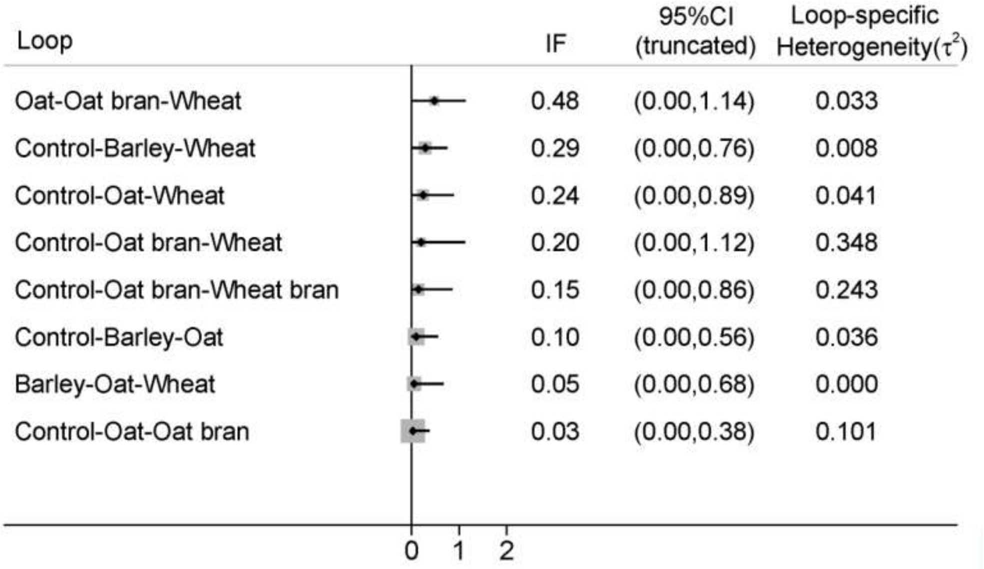

Supplemental Table 19 Node-splitting method for assessment of inconsistency for LDL cholesterol

Comparisons	Direct		Indirect		Difference			
	Coefficient	SE	Coefficient	SE	Coefficient	SE	P^a	tau
Control vs Barley	-0.0843041	0.1187659	-0.2814101	0.2006862	0.197106	0.2332019	0.40	0.2530689
Control vs Brown rice	-	-	-	-	-	-	-	-
Control vs Oat	-0.1660878	0.0587448	-0.1707995	0.1703731	0.0047118	0.180854	0.98	0.2543625
Control vs Oat bran	-0.3646078	0.0749266	-0.1627195	0.1304221	-0.2018882	0.1515467	0.18	0.2498449
Control vs Wheat	-0.0495226	0.1656789	-0.0902376	0.1479257	0.040715	0.2221369	0.86	0.2541878
Control vs Wheat bran	-0.0837884	0.1984157	-0.1903042	0.1064717	0.1065157	0.2251754	0.64	0.2540091
Barley vs Oat	0.0189416	0.2452094	-0.0440415	0.1250387	0.0629831	0.2752523	0.82	0.2542608
Barley vs Wheat	0.3299996	0.3090462	-0.0062023	0.1576500	0.3362019	0.3469338	0.33	0.2526716
Oat vs Oat bran	-0.1609932	0.1177595	-0.1359142	0.1022543	-0.025079	0.1560921	0.87	0.2545929
Oat vs Wheat	0.3599993	0.3285994	0.0552015	0.1262218	0.3047979	0.3520078	0.39	0.2531356
Oat bran vs Wheat	0.0524502	0.1746718	0.3793969	0.1489792	-0.3269468	0.2297094	0.16	0.2494842
Oat bran vs Wheat bran	0.132807	0.0819543	0.2393328	0.2098506	-0.1065258	0.2251745	0.64	0.2540091

[a]*P* for assessment of inconsistency. $P < 0.05$ was considered to indicate a significant inconsistency existed between direct and indirect evidence

Supplemental Table 20 Comparative effects of different whole grains and brans on the control of LDL cholesterol[a]

	Control	Wheat bran	Wheat	Oat bran	Oat	Brown rice	Barley
Control	-	-0.17 (-0.35,0.01)	-0.07 (-0.29,0.14)	-0.32 (-0.44,-0.19)	-0.17 (-0.28,-0.07)	-0.01 (-0.25,0.22)	-0.14 (-0.33,0.06)
Wheat bran		-	0.10 (-0.17,0.36)	-0.15 (-0.30,-0.00)	-0.00 (-0.20,0.20)	0.15 (-0.14,0.45)	0.03 (-0.23,0.30)
Wheat			-	-0.24 (-0.47,-0.02)	-0.10 (-0.33,0.13)	0.06 (-0.26,0.38)	-0.06 (-0.34,0.21)
Oat bran				-	0.15 (-0.00,0.29)	0.30 (0.04,0.57)	0.18 (-0.05,0.41)
Oat					-	0.16 (-0.10,0.41)	0.04 (-0.18,0.25)
Brown rice						-	-0.12 (-0.43,0.18)
Barley							-

[a]Mean differences with 95% confidence intervals (column vs row). Mean differences lower than 0 indicate that the treatments specified in the row are more efficacious than those in the column

Supplemental Table 21 Detailed ranking results of the comparative effects of different whole grains and brans on the control of LDL cholesterol. The larger the SUCRA value, the better the treatment. SUCRA, surface under the cumulative ranking curve

Treatments	SUCRA (%)	MeanRank
Control	13.8	6.2
Barley	53.4	3.8
Brown rice	24.0	5.6
Oat	63.6	3.2
Oat bran	97.4	1.2
Wheat	37.1	4.8
Wheat bran	60.7	3.4

Supplemental Table 22 Sensitivity analysis using an inconsistency model for LDL cholesterol[a]

	Control	Wheat bran	Wheat	Oat bran	Oat	Brown rice	Barley
Control	23.8	0.00 (-0.30,0.31)	-0.16 (-0.29,-0.02)	0.09 (-0.46,0.64)	-0.05 (-0.33,0.23)	-0.02 (-0.25,0.22)	-0.08 (-0.32,0.15)
Wheat bran		47.7	0.03 (-0.48,0.54)	-0.28 (-0.74,0.18)	-0.07 (-0.48,0.34)	0.07 (-0.39,0.53)	-0.00 (-0.46,0.46)
Wheat			39.7	-0.32 (-0.72,0.09)	-0.11 (-0.46,0.25)	0.03 (-0.37,0.44)	-0.03 (-0.44,0.37)
Oat bran				94.7	0.21 (-0.07,0.48)	0.35 (0.01,0.69)	0.28 (-0.06,0.62)
Oat					65.5	0.14 (-0.13,0.41)	0.07 (-0.20,0.34)
Brown rice						31.4	-0.07 (-0.40,0.27)
Barley							47.2

[a]Mean differences with 95% confidence intervals (row vs column). Mean differences lower than 0 indicate that the treatments specified in the row are more efficacious than those in the column. SUCRA values are given in the diagonal of parenthesis. The larger the SUCRA value, the better the treatment. SUCRA, surface under the cumulative ranking curve

Supplemental Table 23 Sensitivity analysis based on the studies only included dyslipidemic participants for LDL cholesterol[a]

	Control	Wheat bran	Wheat	Oat bran	Oat	Brown rice	Barley
Control	16.5	-0.28 (-0.50,-0.05)	-0.04 (-0.28,0.20)	-0.41 (-0.56,-0.25)	-0.19 (-0.32,-0.07)	-0.03 (-0.33,0.28)	-0.11 (-0.36,0.14)
Wheat bran		74.0	0.24 (-0.07,0.54)	-0.13 (-0.30,0.04)	0.08 (-0.16,0.33)	0.25 (-0.14,0.63)	0.17 (-0.17,0.50)
Wheat			28.5	-0.37 (-0.62,-0.11)	-0.15 (-0.41,0.10)	0.01 (-0.38,0.40)	-0.07 (-0.39,0.25)
Oat bran				98.1	0.21 (0.04,0.39)	0.38 (0.03,0.72)	0.29 (0.01,0.58)
Oat					62.1	0.16 (-0.17,0.50)	0.08 (-0.18,0.35)
Brown rice						28.0	-0.08 (-0.48,0.31)
Barley							42.8

[a]Mean differences with 95% confidence intervals (row vs column). Mean differences lower than 0 indicate that the treatments specified in the row are more efficacious than those in the column. SUCRA values are given in the diagonal of parenthesis. The larger the SUCRA value, the better the treatment. SUCRA, surface under the cumulative ranking curve

Supplemental Table 24 Sensitivity analysis based on the studies with parallel design for LDL cholesterol[a]

	Control	Wheat bran	Wheat	Oat bran	Oat	Brown rice	Barley
Control	16.8	-0.23 (-0.46,0.01)	-0.14 (-0.39,0.11)	-0.39 (-0.55,-0.23)	-0.18 (-0.30,-0.06)	0.05 (-0.26,0.35)	-0.20 (-0.71,0.31)
Wheat bran		63.5	0.09 (-0.22,0.40)	-0.16 (-0.34,0.01)	0.05 (-0.20,0.30)	0.28 (-0.11,0.66)	0.03 (-0.52,0.59)
Wheat			47.6	-0.25 (-0.50,0.00)	-0.04 (-0.30,0.23)	0.19 (-0.21,0.59)	-0.06 (-0.62,0.51)
Oat bran				94.9	0.21 (0.04,0.39)	0.44 (0.10,0.78)	0.19 (-0.33,0.72)
Oat					55.8	0.23 (-0.10,0.56)	-0.02 (-0.52,0.48)
Brown rice						15.6	-0.25 (-0.84,0.35)
Barley							55.9

[a]Mean differences with 95% confidence intervals (row vs column). Mean differences lower than 0 indicate that the treatments specified in the row are more efficacious than those in the column. SUCRA values are given in the diagonal of parenthesis. The larger the SUCRA value, the better the treatment. SUCRA, surface under the cumulative ranking curve

Supplemental Table 25 Sensitivity analysis when removing the studies with cluster crossover design for LDL cholesterol[a]

	Control	Wheat bran	Wheat	Oat bran	Oat	Brown rice	Barley
Control	13.9	-0.18 (-0.38,0.02)	-0.07 (-0.29,0.14)	-0.32 (-0.45,-0.19)	-0.17 (-0.28,-0.06)	-0.02 (-0.25,0.22)	-0.14 (-0.34,0.07)
Wheat bran		62.9	0.11 (-0.17,0.38)	-0.14 (-0.30,0.02)	0.01 (-0.20,0.23)	0.16 (-0.15,0.47)	0.04 (-0.24,0.32)
Wheat			36.2	-0.24 (-0.47,-0.02)	-0.09 (-0.33,0.14)	0.06 (-0.26,0.38)	-0.06 (-0.34,0.22)
Oat bran				97.2	0.15 (-0.00,0.30)	0.30 (0.03,0.57)	0.18 (-0.05,0.42)
Oat					62.0	0.15 (-0.11,0.41)	0.03 (-0.19,0.25)
Brown rice						24.3	-0.12 (-0.43,0.19)
Barley							53.5

[a]Mean differences with 95% confidence intervals (row vs column). Mean differences lower than 0 indicate that the treatments specified in the row are more efficacious than those in the column. SUCRA values are given in the diagonal of parenthesis. The larger the SUCRA value, the better the treatment. SUCRA, surface under the cumulative ranking curve

Supplemental Table 26 Sensitivity analysis when excluding the trials with the duration less than 3 weeks for LDL cholesterol[a]

	Control	Wheat bran	Wheat	Oat bran	Oat	Brown rice	Barley
Control	14.1	-0.16 (-0.35,0.03)	-0.08 (-0.29,0.14)	-0.33 (-0.46,-0.20)	-0.17 (-0.28,-0.06)	-0.01 (-0.25,0.22)	-0.14 (-0.34,0.06)
Wheat bran		59.8	0.08 (-0.19,0.35)	-0.17 (-0.33,-0.01)	-0.01 (-0.22,0.20)	0.14 (-0.16,0.45)	0.02 (-0.25,0.30)
Wheat			37.5	-0.25 (-0.47,-0.03)	-0.09 (-0.32,0.14)	0.06 (-0.26,0.38)	-0.06 (-0.33,0.22)
Oat bran				98.0	0.16 (0.01,0.31)	0.31 (0.04,0.58)	0.19 (-0.04,0.42)
Oat					63.5	0.15 (-0.10,0.41)	0.03 (-0.18,0.25)
Brown rice						23.4	-0.12 (-0.43,0.19)
Barley							53.7

[a]Mean differences with 95% confidence intervals (row vs column). Mean differences lower than 0 indicate that the treatments specified in the row are more efficacious than those in the column. SUCRA values are given in the diagonal of parenthesis. The larger the SUCRA value, the better the treatment. SUCRA, surface under the cumulative ranking curve

Supplemental Table 27 Sensitivity analysis when excluding the trials with the duration less than 4 weeks for LDL cholesterol[a]

	Control	Wheat bran	Wheat	Oat bran	Oat	Brown rice	Barley
Control	15.5	-0.15 (-0.35,0.04)	-0.07 (-0.29,0.16)	-0.32 (-0.45,-0.19)	-0.16 (-0.27,-0.05)	-0.02 (-0.25,0.22)	-0.12 (-0.35,0.10)
Wheat bran		60.0	0.09 (-0.19,0.37)	-0.17 (-0.33,-0.00)	-0.00 (-0.22,0.21)	0.14 (-0.17,0.45)	0.03 (-0.26,0.32)
Wheat			36.6	-0.25 (-0.49,-0.02)	-0.09 (-0.33,0.15)	0.05 (-0.28,0.38)	-0.06 (-0.35,0.24)
Oat bran				97.8	0.16 (0.01,0.32)	0.31 (0.03,0.58)	0.20 (-0.06,0.45)
Oat					62.9	0.14 (-0.12,0.41)	0.03 (-0.21,0.28)
Brown rice						25.1	-0.11 (-0.43,0.21)
Barley							52.0

[a]Mean differences with 95% confidence intervals (row vs column). Mean differences lower than 0 indicate that the treatments specified in the row are more efficacious than those in the column. SUCRA values are given in the diagonal of parenthesis. The larger the SUCRA value, the better the treatment. SUCRA, surface under the cumulative ranking curve

Supplemental Table 28 Sensitivity analysis when excluding the trials with intervention duration of 16 weeks and 12 months for LDL cholesterol[a]

	Control	Wheat bran	Wheat	Oat bran	Oat	Brown rice	Barley
Control	10.3	-0.17 (-0.35,0.02)	-0.07 (-0.29,0.14)	-0.31 (-0.44,-0.19)	-0.17 (-0.27,-0.06)	-0.10 (-0.36,0.16)	-0.14 (-0.33,0.06)
Wheat bran		58.3	0.09 (-0.17,0.36)	-0.15 (-0.30,0.00)	-0.00 (-0.20,0.20)	0.07 (-0.25,0.39)	0.03 (-0.24,0.30)
Wheat			32.6	-0.24 (-0.46,-0.02)	-0.09 (-0.32,0.14)	-0.03 (-0.37,0.31)	-0.06 (-0.34,0.21)
Oat bran				96.6	0.15 (-0.00,0.30)	0.21 (-0.08,0.51)	0.18 (-0.05,0.41)
Oat					60.2	0.07 (-0.22,0.35)	0.03 (-0.18,0.25)
Brown rice						42.1	-0.04 (-0.37,0.29)
Barley							49.8

[a]Mean differences with 95% confidence intervals (row vs column). Mean differences lower than 0 indicate that the treatments specified in the row are more efficacious than those in the column. SUCRA values are given in the diagonal of parenthesis. The larger the SUCRA value, the better the treatment. SUCRA, surface under the cumulative ranking curve

Supplemental Table 29 Sensitivity analysis based on the studies that with low risk of bias for LDL cholesterol[a]

	Control	Wheat bran	Wheat	Oat bran	Oat	Brown rice	Barley
Control	23.1	-0.21 (-0.44,0.03)	-0.10 (-0.41,0.22)	-0.33 (-0.49,-0.18)	-0.20 (-0.34,-0.06)	0.30 (-0.29,0.89)	-0.21 (-0.75,0.32)
Wheat bran		62.4	0.11 (-0.24,0.46)	-0.13 (-0.30,0.05)	0.01 (-0.24,0.26)	0.51 (-0.12,1.14)	-0.01 (-0.59,0.57)
Wheat			42.7	-0.24 (-0.54,0.06)	-0.10 (-0.42,0.22)	0.40 (-0.27,1.06)	-0.12 (-0.73,0.49)
Oat bran				90.5	0.14 (-0.04,0.32)	0.63 (0.03,1.24)	0.12 (-0.43,0.67)
Oat					61.4	0.50 (-0.11,1.10)	-0.02 (-0.54,0.50)
Brown rice						9.0	-0.51 (-1.31,0.28)
Barley							61.0

[a]Mean differences with 95% confidence intervals (row vs column). Mean differences lower than 0 indicate that the treatments specified in the row are more efficacious than those in the column. SUCRA values are given in the diagonal of parenthesis. The larger the SUCRA value, the better the treatment. SUCRA, surface under the cumulative ranking curve

Supplemental Table 30 Overall GRADE quality of evidence for LDL cholesterol from network meta-analysis[a]

Comparisons	Study limitations	Imprecision	Heterogeneity and Inconsistency	Indirectness	Publication bias	Confidence in MD for overall change in LDL
Barley vs Control	100% of the estimate from studies at high risk, and 0% at moderate risk	MD -0.14, 95% CI (-0.33,0.06)	Moderate heterogeneity according to I^2 (51.8%) and *P* (0.04) in direct comparisons No inconsistency between the direct and indirect estimate (Node-split *P*= 0.40 and *tau*= 0.25)	The treatment effects were not significantly influenced by clinical modifiers in the subgroup analyses	Undetectable by the routine method The comparison-adjusted funnel plot for the network is not suggestive of any dominant publication bias	Very Low (Downgrade by three levels due to study limitations (for two levels) and imprecision)
Brown rice vs Control	60% of the estimate from studies at high risk, and 20% at moderate risk	MD -0.01, 95% CI (-0.25,0.22)	Moderate heterogeneity according to I^2 (64.4%) and *P* (0.01) in direct comparisons Only direct comparisons and no node-splitting inconsistency	The treatment effects were not significantly influenced by clinical modifiers in the subgroup analyses	Undetectable by the routine method The comparison-adjusted funnel plot for the network is not suggestive of any dominant publication bias	Low (Downgrade by two levels due to study limitations and imprecision)
Oat vs Control	27.2% of the estimate from studies at high risk, and 27.2% at moderate risk	MD -0.17, 95% CI (-0.28,-0.07)	High heterogeneity according to I^2 (82.6%) and *P* (<0.01) in direct comparisons No inconsistency between the direct and indirect estimate (Node-split *P*= 0.98 and *tau*= 0.25)	The treatment effects were not significantly influenced by clinical modifiers in the subgroup analyses	Undetectable by the routine method The comparison-adjusted funnel plot for the network is not suggestive of any dominant publication bias	Very Low (Downgrade by three levels due to study limitations and Heterogeneity and Inconsistency)

Supplemental Table 30 Continued

Comparisons	Study limitations	Imprecision	Heterogeneity and Inconsistency	Indirectness	Publication bias	Confidence in MD for overall change in LDL
Oat bran vs Control	0% of the estimate from studies at high risk, and 100% at moderate risk	MD -0.32, 95% CI (-0.44,-0.19)	High heterogeneity according to I^2 (88.2%) and *P* (<0.01) in direct comparisons No inconsistency between the direct and indirect estimate (Node-split *P*= 0.18 and *tau*=0.24)	The treatment effects were not significantly influenced by clinical modifiers in the subgroup analyses	Undetectable by the routine method The comparison-adjusted funnel plot for the network is not suggestive of any dominant publication bias	Moderate (Downgrade by one level due to Heterogeneity and Inconsistency)
Wheat vs Control	50% of the estimate from studies at high risk, and 25% at moderate risk	MD -0.07, 95% CI (-0.29,0.14)	Mild heterogeneity according to I^2 (17.4%) and *P* (0.29) in direct comparisons No inconsistency between the direct and indirect estimate (Node-split *P*= 0.86 and *tau*= 0.25)	The treatment effects were not significantly influenced by clinical modifiers in the subgroup analyses	Undetectable by the routine method The comparison-adjusted funnel plot for the network is not suggestive of any dominant publication bias	Low (Downgrade by two levels due to study limitations and imprecision)
Wheat bran vs Control	50% of the estimate from studies at high risk, and 50% at moderate risk	MD -0.17, 95% CI (-0.35,0.01)	Mild heterogeneity according to I^2 (0%) and *P* (0.91) in direct comparisons No inconsistency between the direct and indirect estimate (Node-split *P*= 0.64 and *tau*=0.25)	The treatment effects were not significantly influenced by clinical modifiers in the subgroup analyses	Undetectable by the routine method The comparison-adjusted funnel plot for the network is not suggestive of any dominant publication bias	Low (Downgrade by two levels due to study limitations and imprecision)

Supplemental Table 30 Continued

Comparisons	Study limitations	Imprecision	Heterogeneity and Inconsistency	Indirectness	Publication bias	Confidence in MD for overall change in LDL
Barley vs Oat	0% of the estimate from studies at high risk, and 100% at moderate risk	MD -0.02, 95% CI (-0.33, 0.29)	Mild heterogeneity according to I^2 (0%) and *P* (0.92) in direct comparisons No inconsistency between the direct and indirect estimate (Node-split *P*= 0.82 and *tau*= 0.25)	The treatment effects were not significantly influenced by clinical modifiers in the subgroup analyses	Undetectable by the routine method The comparison-adjusted funnel plot for the network is not suggestive of any dominant publication bias	Moderate (Downgrade by one level due to imprecision)
Barley vs Wheat	0% of the estimate from studies at high risk, and 100% at moderate risk	MD -0.06 95% CI (-0.34,0.21)	Only one head-to-head study, and no heterogeneity No inconsistency between the direct and indirect estimate (Node-split *P*= 0.33 and *tau*= 0.25)	The treatment effects were not significantly influenced by clinical modifiers in the subgroup analyses	Undetectable by the routine method The comparison-adjusted funnel lot for the network is not suggestive of any dominant publication bias	Moderate (Downgrade by one level due to imprecision)
Oat vs Oat bran	0% of the estimate from studies at high risk, and 100% at moderate risk	MD 0.15, 95% CI (-0.00,0.29)	Low heterogeneity according to I^2 (38.3%) and *P* (0.07) in direct comparisons No inconsistency between the direct and indirect estimate (Node-split *P*= 06477 and *tau*= 03130)	The treatment effects were not significantly influenced by clinical modifiers in the subgroup analyses	Undetectable by the routine method The comparison-adjusted funnel plot for the network is not suggestive of any dominant publication bias	Moderate (Downgrade by one level due to imprecision)

Supplemental Table 30 Continued

Comparisons	Study limitations	Imprecision	Heterogeneity and Inconsistency	Indirectness	Publication bias	Confidence in MD for overall change in LDL
Oat vs Wheat	0% of the estimate from studies at high risk, and 100% at moderate risk	MD -0.10, 95% CI (-0.33,0.13)	Only one head-to-head study, and no heterogeneity No inconsistency between the direct and indirect estimate (Node-split *P*= 0.87 and *tau*= 0.25)	The treatment effects were not significantly influenced by clinical modifiers in the subgroup analyses	Undetectable by the routine method The comparison-adjusted funnel plot for the network is not suggestive of any dominant publication bias	Moderate (Downgrade by one level due to imprecision)
Oat bran vs Wheat	0% of the estimate from studies at high risk, and 50% at moderate risk	MD -0.24, 95% CI (-0.47,-0.02)	Mild heterogeneity according to I^2 (0%) and *P* (0.48) in direct comparisons No inconsistency between the direct and indirect estimate (Node-split *P*= 0.39 and *tau*= 0.25)	The treatment effects were not significantly influenced by some clinical modifiers in the subgroup analyses	Undetectable by the routine method The comparison-adjusted funnel plot for the network is not suggestive of any dominant publication bias	High
Oat bran vs Wheat bran	0% of the estimate from studies at high risk, and 100% at moderate risk	MD -0.15, 95% CI (-0.30,-0.00)	High heterogeneity according to I^2 (82.9%) and *P* (< 0.01) in direct comparisons No inconsistency between the direct and indirect estimate (Node-split *P*= 0.64 and *tau*= 0.25)	The treatment effects were not significantly influenced by clinical modifiers in the subgroup analyses	Undetectable by the routine method The comparison-adjusted funnel plot for the network is not suggestive of any dominant publication bias	Low (Downgrade by two levels due to imprecision and Heterogeneity and Inconsistency)

[a]The quality of evidence of network estimates for all outcomes by using the GRADE framework, which characterizes the quality of a body of evidence on the basis of the study limitations, imprecision, inconsistency, indirectness, and publication bias.

Supplemental Fig. 7 Pairwise meta-analysis of HDL cholesterol. The square represents the overall estimated effects, and the results were obtained from a random-effects model. MD lower than 0 indicate that the former treatment is more efficacious than latter. MD, mean differences

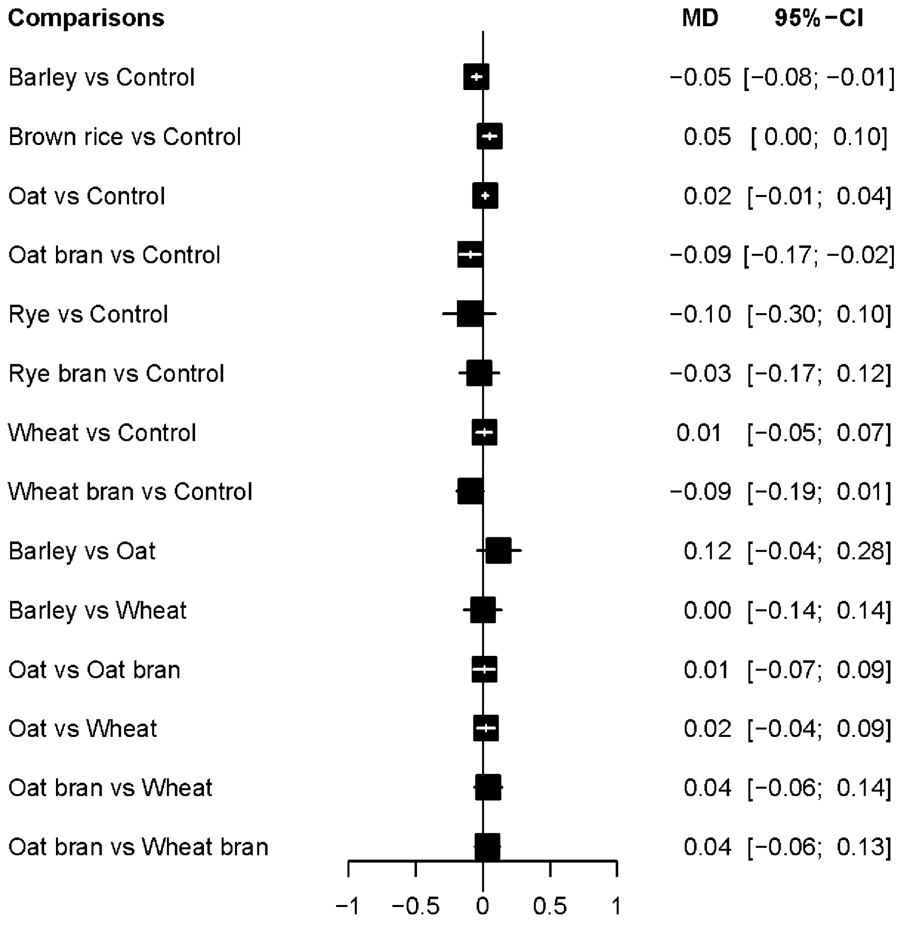

Supplemental Table 31 Pairwise meta-analysis results for HDL cholesterol

Comparisons	No. of comparisons	Mean differences (95% CI)[a]	I^2	τ^2	P^b	P^c (Egger's test)
Barley vs Control	5	-0.05 (-0.08, -0.01)	0%	0.00	0.95	0.02
Brown rice vs Control	8	0.05 (-0.00, 0.10)	0%	0.00	0.73	0.01
Oat vs Control	27	0.02 (-0.01, 0.04)	56.4%	0.00	<0.01	0.85
Oat bran vs Control	19	-0.09 (-0.17, -0.02)	65.5%	0.02	<0.01	0.03
Rye vs Control	2	-0.10 (-0.30, 0.10)	0%	0.00	0.97	-
Rye bran vs Control	1	-0.03 (-0.17, 0.12)	-	-	-	-
Wheat vs Control	6	0.01 (-0.05, 0.07)	0%	0.00	0.92	0.63
Wheat bran vs Control	5	-0.09 (-0.19, 0.01)	0%	0.00	0.72	0.54
Barley vs Oat	2	0.12 (-0.04, 0.28)	41.4%	0.00	0.19	-
Barley vs Wheat	1	0.00 (-0.14, 0.14)	-	-	-	-
Oat vs Oat bran	9	0.01 (-0.07, 0.09)	0%	0.00	0.99	<0.01
Oat vs Wheat	2	0.02 (-0.04, 0.09)	0%	0.00	0.93	-
Oat bran vs Wheat	4	0.04 (-0.06, 0.14)	0%	0.00	0.97	-
Oat bran vs Wheat bran	10	0.04 (-0.06, 0.13)	88.0%	0.01	<0.01	<0.01

[a]Mean differences lower than 0 indicate that the former treatment is more efficacious than latter

[b]*P* for the heterogeneity of pairwise meta-analysis. Heterogeneity was assessed by using Cochran's test, and $P < 0.05$ was considered to indicate significant heterogeneity across studies

[c]*P* for Egger's test. $P < 0.05$ was considered to indicate a significant small-study effects

Supplemental Table 32 Contribution of direct evidence to the network for HDL cholesterol

Comparisons	No. of comparisons	Contribution to the network (%)
Control vs Barley	5	14.2
Control vs Brown rice	8	14.7
Control vs Oat	27	16.3
Control vs Oat bran	19	15.7
Control vs Wheat	6	7.5
Control vs Wheat bran	5	7.6
Barley vs Oat	2	0.9
Barley vs Wheat	1	2.0
Oat vs Oat bran	9	3.4
Oat vs Wheat	2	5.8
Oat bran vs Wheat	4	3.6
Oat bran vs Wheat bran	10	8.5

Supplemental Fig. 8 Evaluation of inconsistency by using loop-specific approach for HDL cholesterol. IFs along with their confidence intervals are displayed. IFs are calculated as the absolute difference between direct and indirect estimates and therefore confidence intervals are truncated to 0. Loops that their lower CI limit does not reach the 0 line are considered to present statistically significant inconsistency. IF, inconsistency factor

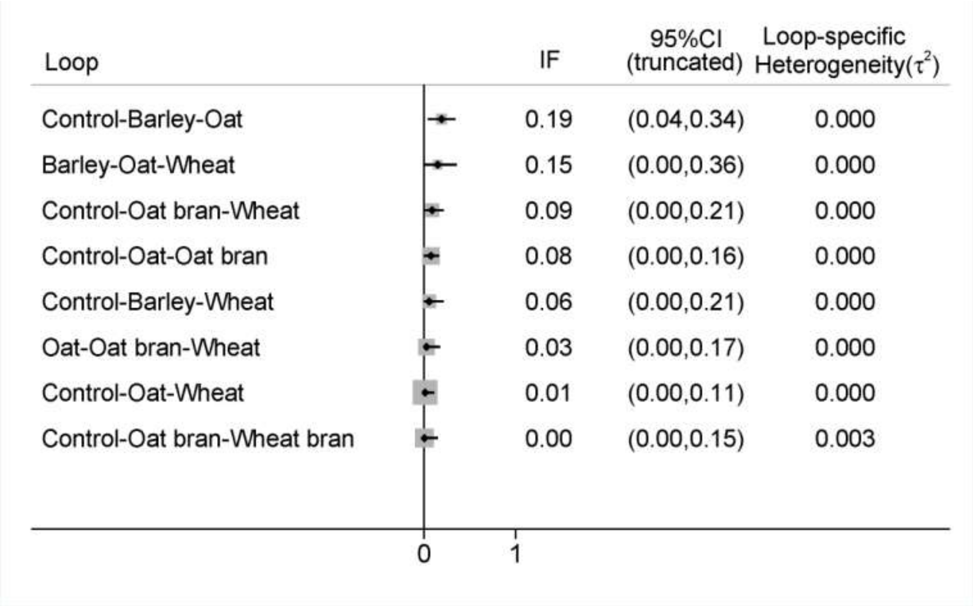

Supplemental Table 33 Node-splitting method for assessment of inconsistency for HDL cholesterol

Comparisons	Direct		Indirect		Difference			
	Coefficient	SE	Coefficient	SE	Coefficient	SE	P^a	tau
Control vs Barley	-0.0456562	0.0272874	0.0692908	0.0573057	-0.1149471	0.0634762	0.07	0.0378725
Control vs Brown rice	-	-	-	-	-	-	-	-
Control vs Oat	0.0116275	0.0137193	-0.0244189	0.0407305	0.0360464	0.0428895	0.40	0.0389282
Control vs Oat bran	-0.0405477	0.0212707	0.0339962	0.0384744	-0.0745439	0.0434447	0.09	0.0370801
Control vs Wheat	0.0118313	0.0340556	-0.0351742	0.0343347	0.0470055	0.048366	0.33	0.0395221
Control vs Wheat bran	-0.0932892	0.0576188	-0.1025073	0.0346987	0.0092181	0.06724	0.89	0.0392321
Barley vs Oat	-1.23E-01	0.0763319	0.0550953	0.0286655	-0.1776244	0.0815889	0.03	0.0372694
Barley vs Wheat	-4.48E-09	0.0811743	0.0148543	0.0363726	-0.0148543	0.0889507	0.87	0.0393848
Oat vs Oat bran	-0.003186	0.0433185	-0.0410133	0.0251536	0.0378273	0.0498378	0.45	0.0389449
Oat vs Wheat	-0.0233938	0.0457584	-0.0176365	0.0307890	-0.0057574	0.0551614	0.92	0.0396162
Oat bran vs Wheat	-0.0393309	0.0558842	0.0297233	0.0328201	-0.0690542	0.0648094	0.29	0.0391678
Oat bran vs Wheat bran	-0.0781303	0.0283321	-0.0689122	0.0609849	-0.0092182	0.0672695	0.89	0.0392321

^aP for assessment of inconsistency. P <0.05 was considered to indicate a significant inconsistency existed between direct and indirect evidence

Supplemental Table 34 Comparative effects of different whole grains and brans on the control of HDL cholesterol[a]

	Control	Wheat bran	Wheat	Oat bran	Oat	Brown rice	Barley
Control	-	-0.12 (-0.19,-0.05)	-0.03 (-0.09,0.03)	-0.08 (-0.12,-0.03)	-0.01 (-0.04,0.03)	0.03 (-0.05,0.11)	-0.02 (-0.09,0.05)
Wheat bran		-	0.09 (0.00,0.18)	0.04 (-0.02,0.10)	0.11 (0.03,0.19)	0.15 (0.04,0.26)	0.10 (-0.00,0.20)
Wheat			-	-0.05 (-0.12,0.02)	0.02 (-0.04,0.09)	0.06 (-0.05,0.16)	0.01 (-0.08,0.10)
Oat bran				-	0.07 (0.02,0.12)	0.11 (0.01,0.20)	0.06 (-0.02,0.14)
Oat					-	0.03 (-0.06,0.12)	-0.01 (-0.09,0.06)
Brown rice						-	-0.05 (-0.16,0.06)
Barley							-

[a]Mean differences with 95% confidence intervals (column vs row). Mean differences lower than 0 indicate that the treatments specified in the row are more efficacious than those in the column

Supplemental Table 35 Detailed ranking results of the comparative effects of different whole grains and brans on the control of HDL cholesterol. The larger the SUCRA value, the better the treatment. SUCRA, surface under the cumulative ranking curve

Treatments	SUCRA (%)	MeanRank
Control	63.1	3.2
Barley	37.0	4.8
Brown rice	91.1	1.5
Oat	74.7	2.5
Oat bran	34.6	4.9
Wheat	49.0	4.1
Wheat bran	0.5	7.0

Supplemental Table 36 Sensitivity analysis using an inconsistency model for HDL cholesterol[a]

	Control	Wheat bran	Wheat	Oat bran	Oat	Brown rice	Barley
Control	56.4	0.04 (-0.06,0.14)	0.02 (-0.01,0.05)	-0.19 (-0.35,-0.03)	-0.08 (-0.16,-0.00)	0.04 (-0.02,0.10)	-0.05 (-0.10,0.01)
Wheat bran		12.9	0.11 (-0.02,0.23)	0.02 (-0.12,0.16)	0.11 (-0.00,0.23)	0.13 (0.00,0.26)	0.05 (-0.08,0.17)
Wheat			68.6	-0.08 (-0.19,0.02)	0.01 (-0.06,0.08)	0.03 (-0.06,0.12)	-0.06 (-0.14,0.03)
Oat bran				18.2	0.09 (0.00,0.18)	0.11 (0.00,0.21)	0.02 (-0.07,0.12)
Oat					79.9	0.02 (-0.05,0.08)	-0.07 (-0.13,-0.01)
Brown rice						87.0	-0.08 (-0.17,-0.00)
Barley							27.0

[a]Mean differences with 95% confidence intervals (row vs column). Mean differences lower than 0 indicate that the treatments specified in the row are more efficacious than those in the column. SUCRA values are given in the diagonal of parenthesis. The larger the SUCRA value, the better the treatment. SUCRA, surface under the cumulative ranking curve

Supplemental Table 37 Sensitivity analysis based on the studies only included dyslipidemic participants for HDL cholesterol[a]

	Control	Wheat bran	Wheat	Oat bran	Oat	Brown rice	Barley
Control	65.3	-0.13 (-0.19,-0.06)	-0.01 (-0.06,0.04)	-0.02 (-0.06,0.02)	0.00 (-0.02,0.03)	0.05 (-0.02,0.13)	-0.03 (-0.07,0.02)
Wheat bran		0.2	0.11 (0.03,0.19)	0.10 (0.04,0.16)	0.13 (0.06,0.20)	0.18 (0.08,0.28)	0.10 (0.02,0.18)
Wheat			49.2	-0.01 (-0.07,0.05)	0.02 (-0.04,0.07)	0.07 (-0.03,0.16)	-0.01 (-0.08,0.05)
Oat bran				36.2	0.03 (-0.02,0.07)	0.08 (-0.01,0.16)	-0.00 (-0.06,0.06)
Oat					69.9	0.05 (-0.03,0.13)	-0.03 (-0.08,0.03)
Brown rice						93.2	-0.08 (-0.17,0.01)
Barley							36.0

[a]Mean differences with 95% confidence intervals (row vs column). Mean differences lower than 0 indicate that the treatments specified in the row are more efficacious than those in the column. SUCRA values are given in the diagonal of parenthesis. The larger the SUCRA value, the better the treatment. SUCRA, surface under the cumulative ranking curve

Supplemental Table 38 Sensitivity analysis based on the studies with parallel design for HDL cholesterol[a]

	Control	Wheat bran	Wheat	Oat bran	Oat	Brown rice	Barley
Control	62.7	-0.14 (-0.24,-0.04)	-0.04 (-0.11,0.03)	-0.10 (-0.15,-0.04)	-0.00 (-0.05,0.04)	0.05 (-0.05,0.15)	0.11 (-0.08,0.30)
Wheat bran		3.7	0.10 (-0.02,0.22)	0.04 (-0.04,0.13)	0.14 (0.03,0.24)	0.19 (0.05,0.34)	0.25 (0.03,0.46)
Wheat			39.2	-0.06 (-0.14,0.02)	0.04 (-0.05,0.12)	0.09 (-0.04,0.22)	0.15 (-0.06,0.35)
Oat bran				16.3	0.09 (0.03,0.16)	0.15 (0.03,0.26)	0.20 (0.01,0.40)
Oat					58.9	0.05 (-0.06,0.17)	0.11 (-0.08,0.30)
Brown rice						80.4	0.06 (-0.16,0.27)
Barley							88.8

[a]Mean differences with 95% confidence intervals (row vs column). Mean differences lower than 0 indicate that the treatments specified in the row are more efficacious than those in the column. SUCRA values are given in the diagonal of parenthesis. The larger the SUCRA value, the better the treatment. SUCRA, surface under the cumulative ranking curve

Supplemental Table 39 Sensitivity analysis when removing the studies with cluster crossover design for HDL cholesterol[a]

	Control	Wheat bran	Wheat	Oat bran	Oat	Brown rice	Barley
Control	73.7	-0.13 (-0.22,-0.05)	-0.03 (-0.09,0.03)	-0.08 (-0.13,-0.03)	-0.01 (-0.04,0.03)	0.03 (-0.06,0.11)	-0.02 (-0.09,0.06)
Wheat bran		2.3	0.11 (0.00,0.21)	0.06 (-0.02,0.13)	0.13 (0.03,0.22)	0.16 (0.04,0.28)	0.12 (0.00,0.23)
Wheat			47.8	-0.05 (-0.12,0.02)	0.02 (-0.05,0.09)	0.06 (-0.05,0.16)	0.01 (-0.08,0.10)
Oat bran				18.7	0.07 (0.02,0.13)	0.11 (0.01,0.20)	0.06 (-0.03,0.15)
Oat					65.2	0.03 (-0.06,0.13)	-0.01 (-0.09,0.07)
Brown rice						85.5	-0.05 (-0.16,0.07)
Barley							56.7

[a]Mean differences with 95% confidence intervals (row vs column). Mean differences lower than 0 indicate that the treatments specified in the row are more efficacious than those in the column. SUCRA values are given in the diagonal of parenthesis. The larger the SUCRA value, the better the treatment. SUCRA, surface under the cumulative ranking curve

Supplemental Table 40 Sensitivity analysis when excluding the trials with the duration less than 3 weeks for HDL cholesterol[a]

	Control	Wheat bran	Wheat	Oat bran	Oat	Brown rice	Barley
Control	72.8	-0.13 (-0.21,-0.05)	-0.03 (-0.09,0.03)	-0.08 (-0.12,-0.03)	-0.01 (-0.04,0.03)	0.03 (-0.06,0.11)	-0.02 (-0.09,0.05)
Wheat bran		2.0	0.10 (0.00,0.20)	0.05 (-0.02,0.12)	0.12 (0.04,0.21)	0.16 (0.04,0.27)	0.11 (0.00,0.22)
Wheat			49.6	-0.05 (-0.12,0.02)	0.02 (-0.05,0.09)	0.06 (-0.05,0.16)	0.01 (-0.08,0.10)
Oat bran				18.6	0.07 (0.01,0.12)	0.10 (0.01,0.20)	0.06 (-0.03,0.14)
Oat					65.3	0.03 (-0.06,0.12)	-0.01 (-0.09,0.07)
Brown rice						85.6	-0.05 (-0.16,0.06)
Barley							56.1

[a]Mean differences with 95% confidence intervals (row vs column). Mean differences lower than 0 indicate that the treatments specified in the row are more efficacious than those in the column. SUCRA values are given in the diagonal of parenthesis. The larger the SUCRA value, the better the treatment. SUCRA, surface under the cumulative ranking curve

Supplemental Table 41 Sensitivity analysis when excluding the trials with the duration less than 4 weeks for HDL cholesterol[a]

	Control	Wheat bran	Wheat	Oat bran	Oat	Brown rice	Barley
Control	74.7	-0.13 (-0.21,-0.05)	-0.03 (-0.10,0.03)	-0.08 (-0.13,-0.03)	-0.00 (-0.04,0.04)	0.03 (-0.05,0.11)	-0.04 (-0.12,0.04)
Wheat bran		2.9	0.10 (-0.00,0.20)	0.05 (-0.02,0.12)	0.13 (0.04,0.22)	0.16 (0.04,0.27)	0.09 (-0.02,0.20)
Wheat			48.6	-0.05 (-0.12,0.02)	0.03 (-0.04,0.10)	0.06 (-0.04,0.16)	-0.01 (-0.11,0.08)
Oat bran				21.2	0.08 (0.02,0.13)	0.11 (0.01,0.20)	0.04 (-0.06,0.13)
Oat						0.03 (-0.06,0.12)	-0.04 (-0.13,0.05)
Brown rice						87.0	-0.07 (-0.19,0.04)
Barley							43.0

[a]Mean differences with 95% confidence intervals (row vs column). Mean differences lower than 0 indicate that the treatments specified in the row are more efficacious than those in the column. SUCRA values are given in the diagonal of parenthesis. The larger the SUCRA value, the better the treatment. SUCRA, surface under the cumulative ranking curve

Supplemental Table 42 Sensitivity analysis when excluding the trials with intervention duration of 16 weeks and 12 months for HDL cholesterol[a]

	Control	Wheat bran	Wheat	Oat bran	Oat	Brown rice	Barley
Control	76.3	-0.13 (-0.21,-0.05)	-0.03 (-0.09,0.03)	-0.08 (-0.12,-0.03)	-0.01 (-0.04,0.03)	0.01 (-0.09,0.10)	-0.02 (-0.09,0.06)
Wheat bran		2.6	0.10 (0.00,0.20)	0.05 (-0.02,0.12)	0.12 (0.04,0.21)	0.13 (0.01,0.26)	0.11 (0.00,0.22)
Wheat			50.8	-0.05 (-0.12,0.02)	0.02 (-0.05,0.09)	0.03 (-0.08,0.15)	0.01 (-0.08,0.10)
Oat bran				19.3	0.07 (0.01,0.12)	0.08 (-0.02,0.19)	0.06 (-0.03,0.14)
Oat					68.7	0.01 (-0.09,0.12)	-0.01 (-0.09,0.07)
Brown rice						74.2	-0.02 (-0.15,0.10)
Barley							58.1

[a]Mean differences with 95% confidence intervals (row vs column). Mean differences lower than 0 indicate that the treatments specified in the row are more efficacious than those in the column. SUCRA values are given in the diagonal of parenthesis. The larger the SUCRA value, the better the treatment. SUCRA, surface under the cumulative ranking curve

Supplemental Table 43 Sensitivity analysis based on the studies with low risk of bias for HDL cholesterol[a]

	Control	Wheat bran	Wheat	Oat bran	Oat	Brown rice	Barley
Control	48.9	-0.10 (-0.17,-0.03)	-0.02 (-0.09,0.05)	-0.03 (-0.07,0.01)	0.01 (-0.02,0.05)	0.09 (-0.02,0.20)	0.14 (-0.02,0.29)
Wheat bran		1.0	0.08 (-0.01,0.17)	0.07 (0.01,0.13)	0.11 (0.04,0.19)	0.19 (0.06,0.32)	0.23 (0.06,0.41)
Wheat			34.6	-0.01 (-0.08,0.07)	0.04 (-0.04,0.11)	0.11 (-0.02,0.24)	0.16 (-0.01,0.33)
Oat bran				26.6	0.04 (-0.01,0.09)	0.12 (-0.00,0.24)	0.16 (0.00,0.33)
Oat					62.2	0.08 (-0.04,0.19)	0.12 (-0.03,0.28)
Brown rice						84.7	0.05 (-0.15,0.24)
Barley							92.0

[a]Mean differences with 95% confidence intervals (row vs column). Mean differences lower than 0 indicate that the treatments specified in the row are more efficacious than those in the column. SUCRA values are given in the diagonal of parenthesis. The larger the SUCRA value, the better the treatment. SUCRA, surface under the cumulative ranking curve

Supplemental Table 44 Overall GRADE quality of evidence for HDL cholesterol from network meta-analysis[a]

Comparisons	Study limitations	Imprecision	Heterogeneity and Inconsistency	Indirectness	Publication bias	Confidence in MD for overall change in HDL
Barley vs Control	100% of the estimate from studies at high risk, and 0% at moderate risk	MD -0.02, 95% CI (-0.09,0.05)	Mild heterogeneity according to I^2 (0%) and *P* (1.00) in direct comparisons No inconsistency between the direct and indirect estimate (Node-split *P*= 0.07 and *tau*= 0.04)	The treatment effects were not significantly influenced by clinical modifiers in the subgroup analyses	Undetectable by the routine method The comparison-adjusted funnel plot for the network is not suggestive of any dominant publication bias	Very Low (Downgrade by three levels due to study limitations (for two levels) and imprecision)
Brown rice vs Control	80% of the estimate from studies at high risk, and 20% at moderate risk	MD 0.03, 95% CI (-0.05,0.11)	Mild heterogeneity according to I^2 (0%) and *P* (0.82) in direct comparisons Only direct comparisons and no node-splitting inconsistency	The treatment effects were not significantly influenced by clinical modifiers in the subgroup analyses	Undetectable by the routine method The comparison-adjusted funnel plot for the network is not suggestive of any dominant publication bias	Very Low (Downgrade by three levels due to study limitations (for two levels), imprecision)
Oat vs Control	40% of the estimate from studies at high risk, and 60% at moderate risk	MD -0.01, 95% CI (-0.04,0.03)	Moderate heterogeneity according to I^2 (56.4%) and *P* (0.08) in direct comparisons No inconsistency between the direct and indirect estimate (Node-split *P*= 0.40 and *tau*= 0.04)	The treatment effects were not significantly influenced by by some clinical modifiers in the subgroup analyses	Undetectable by the routine method The comparison-adjusted funnel plot for the network is not suggestive of any dominant publication bias	Low (Downgrade by two levels due to study limitations and imprecision)

Supplemental Table 44 Continued

Comparisons	Study limitations	Imprecision	Heterogeneity and Inconsistency	Indirectness	Publication bias	Confidence in MD for overall change in HDL
Oat bran vs Control	0% of the estimate from studies at high risk, and 100% at moderate risk	MD -0.08, 95% CI (-0.12,-0.03)	Moderate heterogeneity according to I^2 (65.5%) and *P* (< 0.01) in direct comparisons No inconsistency between the direct and indirect estimate (Node-split *P*= 0.09 and *tau*= 0.04)	The treatment effects were significantly influenced by clinical modifiers in the subgroup analyses	Undetectable by the routine method The comparison-adjusted funnel plot for the network is not suggestive of any dominant publication bias	Moderate (Downgrade by one level due to Indirectness)
Wheat vs Control	50% of the estimate from studies at high risk, and 25% at moderate risk	MD -0.03, 95% CI (-0.09,0.03)	Mild heterogeneity according to I^2 (0%) and *P* (0.91) in direct comparisons No inconsistency between the direct and indirect estimate (Node-split *P*= 0.33 and *tau*= 0.04)	The treatment effects were not significantly influenced by clinical modifiers in the subgroup analyses	Undetectable by the routine method The comparison-adjusted funnel plot for the network is not suggestive of any dominant publication bias	Low (Downgrade by two levels due to study limitations and imprecision)
Wheat bran vs Control	50% of the estimate from studies at high risk, and 50% at moderate risk	MD -0.12, 95% CI (-0.19,-0.05)	Mild heterogeneity according to I^2 (0%) and *P* (0.82) in direct comparisons No inconsistency between the direct and indirect estimate (Node-split *P*= 0.89 and *tau*= 0.04)	The treatment effects were not significantly influenced by clinical modifiers in the subgroup analyses	Undetectable by the routine method The comparison-adjusted funnel plot for the network is not suggestive of any dominant publication bias	Moderate (Downgrade by one level due to study limitations)

Supplemental Table 44 Continued

Comparisons	Study limitations	Imprecision	Heterogeneity and Inconsistency	Indirectness	Publication bias	Confidence in MD for overall change in HDL
Barley vs Oat	0% of the estimate from studies at high risk, and 100% at moderate risk	MD -0.01, 95% CI (-0.09,0.06)	Low heterogeneity according to I^2 (41.4%) and *P* (0.27) in direct comparisons No inconsistency between the direct and indirect estimate (Node-split *P*= 0.03 and *tau*= 0.04)	The treatment effects were not significantly influenced by clinical modifiers in the subgroup analyses	Undetectable by the routine method The comparison-adjusted funnel plot for the network is not suggestive of any dominant publication bias	Moderate (Downgrade by one level due to imprecision)
Barley vs Wheat	0% of the estimate from studies at high risk, and 100% at moderate risk	MD 0.01, 95% CI (-0.08,0.10)	Only one head-to-head study, and no heterogeneity No inconsistency between the direct and indirect estimate (Node-split *P*= 0.87 and *tau*= 0.04)	The treatment effects were not significantly influenced by clinical modifiers in the subgroup analyses	Undetectable by the routine method The comparison-adjusted funnel plot for the network is not suggestive of any dominant publication bias	Moderate (Downgrade by one level due to imprecision)
Oat vs Oat bran	0% of the estimate from studies at high risk, and 100% at moderate risk	MD 0.07, 95% CI (0.02,0.12)	Mild heterogeneity according to I^2 (0%) and *P* (0.98) in direct comparisons No inconsistency between the direct and indirect estimate (Node-split *P*= 0.45 and *tau*= 0.04)	The treatment effects were significantly influenced by clinical modifiers in the subgroup analyses	Undetectable by the routine method The comparison-adjusted funnel plot for the network is not suggestive of any dominant publication bias	Moderate (Downgrade by one level due to Indirectness)

Supplemental Table 44 Continued

Comparisons	Study limitations	Imprecision	Heterogeneity and Inconsistency	Indirectness	Publication bias	Confidence in MD for overall change in HDL
Oat vs Wheat	0% of the estimate from studies at high risk, and 100% at moderate risk	MD 0.02, 95% CI (-0.04,0.09)	Mild heterogeneity according to I^2 (0%) and *P* (0.90) in direct comparisons No inconsistency between the direct and indirect estimate (Node-split *P*= 0.92 and *tau*= 0.04)	The treatment effects were not significantly influenced by clinical modifiers in the subgroup analyses	Undetectable by the routine method The comparison-adjusted funnel plot for the network is not suggestive of any dominant publication bias	Moderate (Downgrade by one level due to imprecision)
Oat bran vs Wheat	0% of the estimate from studies at high risk, and 50% at moderate risk	MD -0.05, 95% CI (-0.12,0.02)	Mild heterogeneity according to I^2 (0%) and *P* (0.97) in direct comparisons No inconsistency between the direct and indirect estimate (Node-split *P*= 0.29 and *tau*= 0.04)	The treatment effects were not significantly influenced by clinical modifiers in the subgroup analyses	Undetectable by the routine method The comparison-adjusted funnel plot for the network is not suggestive of any dominant publication bias	Moderate (Downgrade by one level due to imprecision)
Oat bran vs Wheat bran	0% of the estimate from studies at high risk, and 100% at moderate risk	MD 0.04, 95% CI (-0.02,0.10)	High heterogeneity according to I^2 (88.0%) and *P* (< 0.01) in direct comparisons No inconsistency between the direct and indirect estimate (Node-split *P*= 0.89 and *tau*= 0.04)	The treatment effects were not significantly influenced by clinical modifiers in the subgroup analyses	Undetectable by the routine method The comparison-adjusted funnel plot for the network is not suggestive of any dominant publication bias	Low (Downgrade by two levels due to imprecision and Heterogeneity and Inconsistency)

[a]The quality of evidence of network estimates for all outcomes by using the GRADE framework, which characterizes the quality of a body of evidence on the basis of the study limitations, imprecision, inconsistency, indirectness, and publication bias.

Supplemental Fig. 9 Pairwise meta-analysis of triglycerides. The square represents the overall estimated effects, and the results were obtained from a random-effects model. MD lower than 0 indicate that the former treatment is more efficacious than latter. MD, mean differences

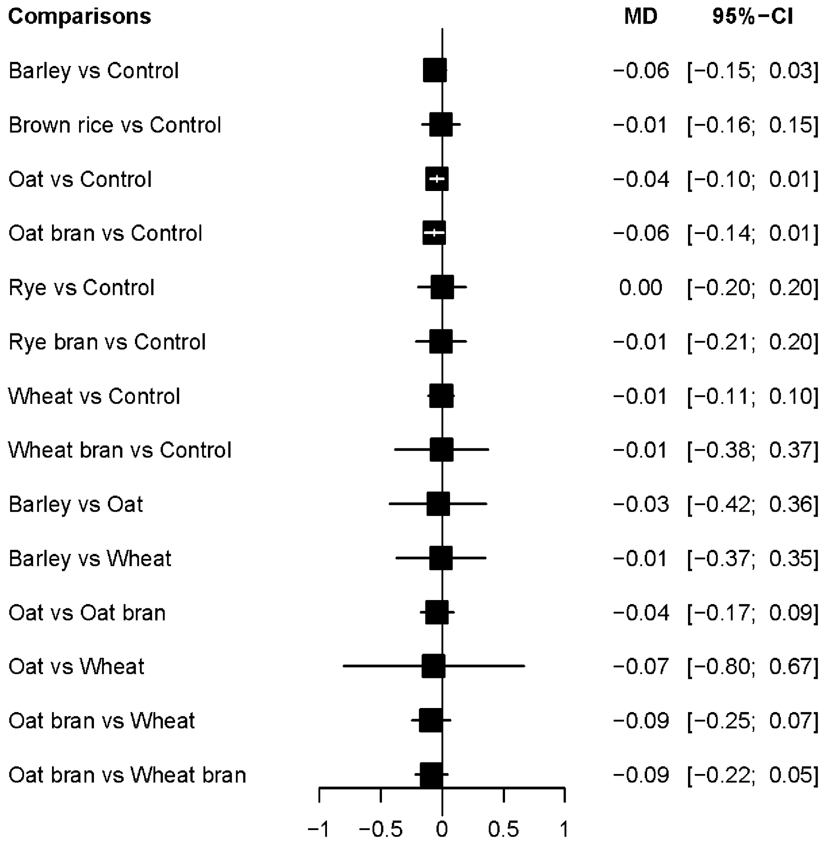

Supplemental Table 45 Pairwise meta-analysis results for triglycerides

Comparisons	No. of comparisons	Mean differences (95% CI)[a]	I^2	τ^2	P^b	P^c (Egger's test)
Barley vs Control	5	-0.06 (-0.15, 0.03)	27.2%	0.00	0.24	0.02
Brown rice vs Control	8	-0.01 (-0.16, 0.15)	0%	0.00	0.62	0.50
Oat vs Control	27	-0.04 (-0.10, 0.01)	42.1%	0.00	0.01	0.04
Oat bran vs Control	20	-0.06 (-0.14, 0.01)	0%	0.00	0.95	0.32
Rye vs Control	2	0.00 (-0.20, 0.20)	0%	0.00	0.33	-
Rye bran vs Control	1	-0.01 (-0.21, 0.20)	-	-	-	-
Wheat vs Control	6	-0.01 (-0.11, 0.10)	0%	0.00	0.99	0.51
Wheat bran vs Control	5	-0.01 (-0.38, 0.37)	0%	0.00	0.35	0.62
Barley vs Oat	2	-0.03 (-0.42, 0.36)	0%	0.00	0.52	-
Barley vs Wheat	1	-0.01 (-0.37, 0.35)	-	-	-	-
Oat vs Oat bran	9	-0.04 (-0.17, 0.09)	0%	0.00	0.09	0.37
Oat vs Wheat	2	-0.07 (-0.80, 0.67)	77.6%	0.23	0.03	-
Oat bran vs Wheat	4	-0.09 (-0.25, 0.07)	0%	0.00	0.46	-
Oat bran vs Wheat bran	10	-0.09 (-0.22, 0.05)	57.7%	0.02	0.01	0.21

[a]Mean differences lower than 0 indicate that the former treatment is more efficacious than latter

[b]*P* for the heterogeneity of pairwise meta-analysis. Heterogeneity was assessed by using Cochran's test, and $P < 0.05$ was considered to indicate significant heterogeneity across studies

[c]*P* for Egger's test. $P < 0.05$ was considered to indicate a significant small-study effects

Supplemental Table 46 Contribution of direct evidence to the network for triglycerides

Comparisons	No. of comparisons	Contribution to the network (%)
Control vs Barley	5	13.3
Control vs Brown rice	8	14.3
Control vs Oat	27	14.8
Control vs Oat bran	20	15.8
Control vs Wheat	6	11.1
Control vs Wheat bran	5	2.2
Barley vs Oat	2	1.0
Barley vs Wheat	1	1.6
Oat vs Oat bran	9	6.3
Oat vs Wheat	2	0.3
Oat bran vs Wheat	4	6.5
Oat bran vs Wheat bran	10	12.8

Supplemental Fig. 10 Evaluation of inconsistency by using loop-specific approach for triglycerides. IFs along with their confidence intervals are displayed. IFs are calculated as the absolute difference between direct and indirect estimates and therefore confidence intervals are truncated to 0. Loops that their lower CI limit does not reach the 0 line are considered to present statistically significant inconsistency. IF, inconsistency factor

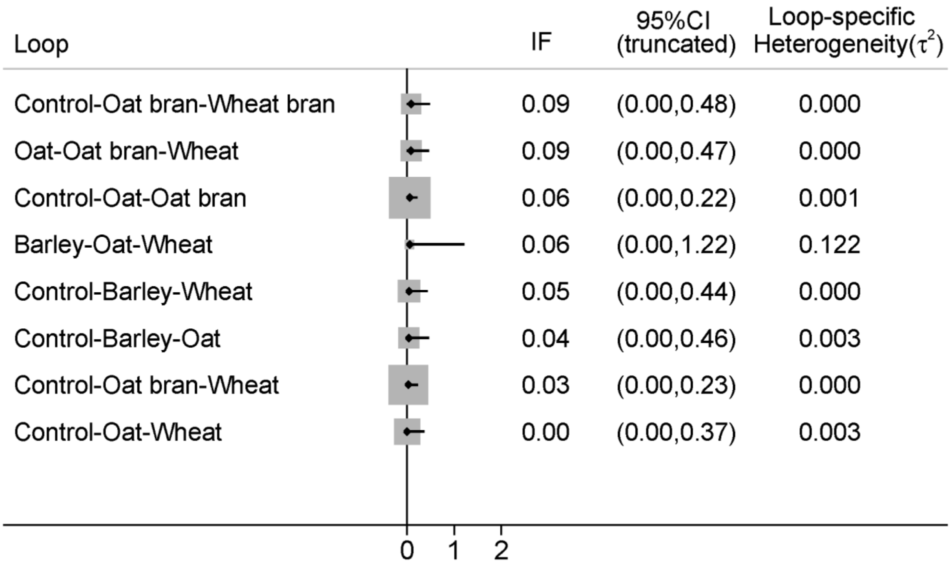

Supplemental Table 47 Node-splitting method for assessment of inconsistency for triglycerides

Comparisons	Direct		Indirect		Difference			
	Coefficient	SE	Coefficient	SE	Coefficient	SE	P^a	tau
Control vs Barley	-0.0416445	0.05738	-0.0503133	0.1463491	0.0086688	0.1573788	0.96	0.0697359
Control vs Brown rice	-	-	-	-	-	-	-	-
Control vs Oat	-0.0586073	0.0295816	-0.0626302	0.1070164	0.0040229	0.1121942	0.97	0.0696149
Control vs Oat bran	-0.0693588	0.0456356	-0.0426665	0.0800254	-0.0266923	0.0936898	0.78	0.0704432
Control vs Wheat	-0.0074508	0.0604894	0.004879	0.0816147	-0.0123298	0.1015761	0.90	0.0700041
Control vs Wheat bran	-0.0046729	0.1989407	0.0542954	0.0670452	-0.0589683	0.209962	0.78	0.0687131
Barley vs Oat	0.0309012	0.206401	-0.0201972	0.0625755	0.0510984	0.2155826	0.81	0.0695311
Barley vs Wheat	1.00E-02	0.1987217	0.0439371	0.0745038	-0.0339372	0.212229	0.87	0.0694869
Oat vs Oat bran	0.0399333	0.0717384	-0.029224	0.0549041	0.0691573	0.0899732	0.44	0.0687797
Oat vs Wheat	0.0448044	0.1720902	0.0570593	0.0568779	-0.0122549	0.1812266	0.95	0.0693659
Oat bran vs Wheat	0.079784	0.093514	0.048556	0.0686998	0.031228	0.1161036	0.79	0.069841
Oat bran vs Wheat bran	0.1147561	0.0539588	0.0557898	0.2028179	0.0589663	0.2099612	0.78	0.0687131

[a]*P* for assessment of inconsistency. $P < 0.05$ was considered to indicate a significant inconsistency existed between direct and indirect evidence

Supplemental Table 48 Comparative effects of different whole grains and brans on the control of triglycerides[a]

	Control	Wheat bran	Wheat	Oat bran	Oat	Brown rice	Barley
Control	-	0.05 (-0.08,0.17)	-0.00 (-0.10,0.09)	-0.06 (-0.14,0.01)	-0.06 (-0.11,-0.00)	0.01 (-0.17,0.18)	-0.04 (-0.15,0.06)
Wheat bran		-	-0.05 (-0.20,0.10)	-0.11 (-0.21,-0.01)	-0.11 (-0.24,0.02)	-0.04 (-0.26,0.17)	-0.09 (-0.26,0.07)
Wheat			-	-0.06 (-0.17,0.05)	-0.06 (-0.16,0.05)	0.01 (-0.19,0.20)	-0.04 (-0.18,0.10)
Oat bran				-	0.00 (-0.08,0.09)	0.07 (-0.12,0.26)	0.02 (-0.11,0.15)
Oat					-	0.06 (-0.12,0.24)	0.02 (-0.10,0.13)
Brown rice						-	-0.05 (-0.25,0.15)
Barley							-

[a]Mean differences with 95% confidence intervals (column vs row). Mean differences lower than 0 indicate that the treatments specified in the row are more efficacious than those in the column

Supplemental Table 49 Detailed ranking results of the comparative effects of different whole grains and brans on the control of triglycerides. The larger the SUCRA value, the better the treatment. SUCRA, surface under the cumulative ranking curve

Treatments	SUCRA (%)	MeanRank
Control	34.0	5.0
Barley	64.4	3.1
Brown rice	40.7	4.6
Oat	76.2	2.4
Oat bran	78.0	2.3
Wheat	39.7	4.6
Wheat bran	17.0	6.0

Supplemental Table 50 Sensitivity analysis using an inconsistency model for triglycerides[a]

	Control	Wheat bran	Wheat	Oat bran	Oat	Brown rice	Barley
Control	32.2	0.03 (-0.17,0.23)	-0.05 (-0.11,0.02)	0.04 (-0.39,0.46)	-0.09 (-0.26,0.09)	0.01 (-0.17,0.18)	-0.04 (-0.15,0.07)
Wheat bran		45.3	-0.00 (-0.41,0.41)	-0.09 (-0.52,0.34)	-0.04 (-0.44,0.35)	0.01 (-0.42,0.44)	-0.04 (-0.44,0.37)
Wheat			40.5	-0.09 (-0.30,0.12)	-0.04 (-0.18,0.10)	0.01 (-0.20,0.22)	-0.03 (-0.20,0.13)
Oat bran				75.4	0.05 (-0.13,0.23)	0.10 (-0.14,0.34)	0.06 (-0.15,0.26)
Oat					63.3	0.05 (-0.13,0.24)	0.01 (-0.12,0.14)
Brown rice						36.9	-0.05 (-0.25,0.16)
Barley							56.4

[a]Mean differences with 95% confidence intervals (row vs column). Mean differences lower than 0 indicate that the treatments specified in the row are more efficacious than those in the column. SUCRA values are given in the diagonal of parenthesis. The larger the SUCRA value, the better the treatment. SUCRA, surface under the cumulative ranking curve

Supplemental Table 51 Sensitivity analysis based on the studies only included dyslipidemic participants for triglycerides[a]

	Control	Wheat bran	Wheat	Oat bran	Oat	Brown rice	Barley
Control	36.4	0.06 (-0.09,0.20)	-0.02 (-0.15,0.10)	-0.06 (-0.15,0.04)	-0.06 (-0.13,0.00)	-0.09 (-0.30,0.13)	0.04 (-0.13,0.20)
Wheat bran		17.7	-0.08 (-0.26,0.10)	-0.11 (-0.22,-0.00)	-0.12 (-0.26,0.03)	-0.14 (-0.40,0.12)	-0.02 (-0.23,0.19)
Wheat			50.7	-0.03 (-0.18,0.11)	-0.04 (-0.17,0.10)	-0.06 (-0.32,0.19)	0.06 (-0.13,0.25)
Oat bran				70.9	-0.00 (-0.11,0.10)	-0.03 (-0.27,0.21)	0.09 (-0.09,0.27)
Oat					74.8	-0.03 (-0.25,0.20)	0.10 (-0.07,0.27)
Brown rice						72.1	0.12 (-0.15,0.39)
Barley							27.5

[a]Mean differences with 95% confidence intervals (row vs column). Mean differences lower than 0 indicate that the treatments specified in the row are more efficacious than those in the column. SUCRA values are given in the diagonal of parenthesis. The larger the SUCRA value, the better the treatment. SUCRA, surface under the cumulative ranking curve

Supplemental Table 52 Sensitivity analysis based on the studies with parallel design for triglycerides[a]

	Control	Wheat bran	Wheat	Oat bran	Oat	Brown rice	Barley
Control	44.4	0.10 (-0.04,0.23)	0.03 (-0.08,0.14)	-0.04 (-0.12,0.05)	-0.05 (-0.11,0.00)	-0.01 (-0.20,0.17)	-0.08 (-0.49,0.33)
Wheat bran		11.7	-0.07 (-0.23,0.09)	-0.13 (-0.24,-0.03)	-0.15 (-0.29,-0.01)	-0.11 (-0.34,0.12)	-0.18 (-0.61,0.25)
Wheat			32.3	-0.07 (-0.18,0.05)	-0.08 (-0.20,0.04)	-0.04 (-0.25,0.17)	-0.11 (-0.53,0.31)
Oat bran				67.7	-0.01 (-0.11,0.08)	0.03 (-0.18,0.23)	-0.05 (-0.46,0.37)
Oat					76.4	0.04 (-0.15,0.23)	-0.03 (-0.43,0.37)
Brown rice						52.1	-0.07 (-0.52,0.38)
Barley							65.3

[a]Mean differences with 95% confidence intervals (row vs column). Mean differences lower than 0 indicate that the treatments specified in the row are more efficacious than those in the column. SUCRA values are given in the diagonal of parenthesis. The larger the SUCRA value, the better the treatment. SUCRA, surface under the cumulative ranking curve

Supplemental Table 53 Sensitivity analysis when removing the studies with cluster crossover design for triglycerides[a]

	Control	Wheat bran	Wheat	Oat bran	Oat	Brown rice	Barley
Control	34.2	0.05 (-0.08,0.17)	-0.00 (-0.10,0.09)	-0.07 (-0.14,0.01)	-0.06 (-0.11,-0.00)	0.01 (-0.17,0.18)	-0.04 (-0.15,0.06)
Wheat bran		17.9	-0.05 (-0.20,0.10)	-0.11 (-0.21,-0.01)	-0.11 (-0.24,0.03)	-0.04 (-0.25,0.17)	-0.09 (-0.25,0.08)
Wheat			39.1	-0.06 (-0.17,0.05)	-0.06 (-0.16,0.05)	0.01 (-0.19,0.21)	-0.04 (-0.18,0.10)
Oat bran				79.5	0.01 (-0.08,0.09)	0.07 (-0.12,0.26)	0.02 (-0.11,0.15)
Oat					76.5	0.06 (-0.12,0.25)	0.02 (-0.10,0.13)
Brown rice						39.5	-0.05 (-0.25,0.15)
Barley							63.1

[a]Mean differences with 95% confidence intervals (row vs column). Mean differences lower than 0 indicate that the treatments specified in the row are more efficacious than those in the column. SUCRA values are given in the diagonal of parenthesis. The larger the SUCRA value, the better the treatment. SUCRA, surface under the cumulative ranking curve

Supplemental Table 54 Sensitivity analysis when excluding the trials with the duration less than 3 weeks for triglycerides[a]

	Control	Wheat bran	Wheat	Oat bran	Oat	Brown rice	Barley
Control	36.6	0.10 (-0.04,0.24)	-0.00 (-0.10,0.09)	-0.06 (-0.14,0.02)	-0.06 (-0.11,-0.00)	0.00 (-0.17,0.17)	-0.05 (-0.15,0.06)
Wheat bran		8.1	-0.10 (-0.26,0.06)	-0.16 (-0.28,-0.04)	-0.16 (-0.30,-0.01)	-0.10 (-0.32,0.13)	-0.14 (-0.32,0.03)
Wheat			41.2	-0.06 (-0.17,0.05)	-0.06 (-0.16,0.05)	0.01 (-0.19,0.20)	-0.04 (-0.18,0.09)
Oat bran				77.2	0.00 (-0.09,0.09)	0.06 (-0.12,0.25)	0.01 (-0.11,0.14)
Oat					77.0	0.06 (-0.12,0.24)	0.01 (-0.10,0.13)
Brown rice						43.6	-0.05 (-0.24,0.15)
Barley							66.3

[a]Mean differences with 95% confidence intervals (row vs column). Mean differences lower than 0 indicate that the treatments specified in the row are more efficacious than those in the column. SUCRA values are given in the diagonal of parenthesis. The larger the SUCRA value, the better the treatment. SUCRA, surface under the cumulative ranking curve

Supplemental Table 55 Sensitivity analysis when excluding the trials with the duration less than 4 weeks for triglycerides[a]

	Control	Wheat bran	Wheat	Oat bran	Oat	Brown rice	Barley
Control	37.8	0.10 (-0.05,0.24)	0.00 (-0.10,0.10)	-0.06 (-0.14,0.02)	-0.06 (-0.11,-0.00)	0.00 (-0.17,0.18)	-0.04 (-0.15,0.07)
Wheat bran		8.5	-0.09 (-0.26,0.07)	-0.16 (-0.28,-0.03)	-0.15 (-0.30,-0.01)	-0.09 (-0.32,0.13)	-0.14 (-0.32,0.05)
Wheat			40.2	-0.06 (-0.17,0.05)	-0.06 (-0.17,0.05)	0.00 (-0.20,0.20)	-0.04 (-0.19,0.10)
Oat bran				77.0	0.00 (-0.09,0.09)	0.06 (-0.13,0.25)	0.02 (-0.12,0.15)
Oat					77.9	0.06 (-0.12,0.24)	0.02 (-0.11,0.14)
Brown rice						44.2	-0.04 (-0.24,0.16)
Barley							64.5

[a]Mean differences with 95% confidence intervals (row vs column). Mean differences lower than 0 indicate that the treatments specified in the row are more efficacious than those in the column. SUCRA values are given in the diagonal of parenthesis. The larger the SUCRA value, the better the treatment. SUCRA, surface under the cumulative ranking curve

Supplemental Table 56 Sensitivity analysis when excluding the trials with intervention duration of 16 weeks and 12 months for triglycerides[a]

	Control	Wheat bran	Wheat	Oat bran	Oat	Brown rice	Barley
Control	38.9	0.04 (-0.09,0.17)	-0.00 (-0.10,0.09)	-0.07 (-0.14,0.01)	-0.06 (-0.11,-0.00)	0.11 (-0.12,0.34)	-0.04 (-0.15,0.06)
Wheat bran		24.0	-0.05 (-0.19,0.10)	-0.11 (-0.21,-0.00)	-0.10 (-0.23,0.03)	0.07 (-0.20,0.33)	-0.09 (-0.25,0.08)
Wheat			44.4	-0.06 (-0.17,0.05)	-0.06 (-0.16,0.05)	0.11 (-0.14,0.36)	-0.04 (-0.18,0.10)
Oat bran				81.6	0.01 (-0.08,0.09)	0.17 (-0.07,0.42)	0.02 (-0.11,0.15)
Oat					78.4	0.17 (-0.07,0.41)	0.02 (-0.10,0.13)
Brown rice						15.9	-0.15 (-0.41,0.10)
Barley							66.8

[a]Mean differences with 95% confidence intervals (row vs column). Mean differences lower than 0 indicate that the treatments specified in the row are more efficacious than those in the column. SUCRA values are given in the diagonal of parenthesis. The larger the SUCRA value, the better the treatment. SUCRA, surface under the cumulative ranking curve

Supplemental Table 57 Sensitivity analysis based on the studies with low risk of bias for triglycerides[a]

	Control	Wheat bran	Wheat	Oat bran	Oat	Brown rice	Barley
Control	34.5	0.06 (-0.07,0.19)	0.04 (-0.08,0.16)	-0.06 (-0.14,0.02)	-0.09 (-0.16,-0.03)	-0.12 (-0.37,0.13)	-0.12 (-0.53,0.28)
Wheat bran		14.4	-0.02 (-0.18,0.14)	-0.12 (-0.23,-0.02)	-0.15 (-0.29,-0.02)	-0.18 (-0.46,0.10)	-0.18 (-0.61,0.24)
Wheat			22.4	-0.10 (-0.22,0.02)	-0.13 (-0.26,0.00)	-0.16 (-0.43,0.12)	-0.16 (-0.58,0.26)
Oat bran				63.9	-0.03 (-0.12,0.06)	-0.06 (-0.32,0.20)	-0.06 (-0.48,0.35)
Oat					75.7	-0.03 (-0.28,0.23)	-0.03 (-0.43,0.37)
Brown rice						72.9	-0.00 (-0.48,0.47)
Barley							66.1

[a]Mean differences with 95% confidence intervals (row vs column). Mean differences lower than 0 indicate that the treatments specified in the row are more efficacious than those in the column. SUCRA values are given in the diagonal of parenthesis. The larger the SUCRA value, the better the treatment. SUCRA, surface under the cumulative ranking curve

Supplemental Table 58 Overall GRADE quality of evidence for triglycerides from network meta-analysis[a]

Comparisons	Study limitations	Imprecision	Heterogeneity and Inconsistency	Indirectness	Publication bias	Confidence in MD for overall change in TG
Barley vs Control	100% of the estimate from studies at high risk, and 0% at moderate risk	MD -0.02, 95% CI (-0.09,0.05)	Low heterogeneity according to I^2 (27.2%) and *P* (0.36) in direct comparisons No inconsistency between the direct and indirect estimate (Node-split *P*= 0.96 and *tau*= 0.07)	The treatment effects were not significantly influenced by clinical modifiers in the subgroup analyses	Undetectable by the routine method The comparison-adjusted funnel plot for the network is not suggestive of any dominant publication bias	Very Low (Downgrade by three levels due to study limitations (for two levels) and imprecision)
Brown rice vs Control	100% of the estimate from studies at high risk, and 0% at moderate risk	MD 0.03, 95% CI (-0.05,0.11)	Mild heterogeneity according to I^2 (0%) and *P* (0.60) in direct comparisons Only direct comparisons and no node-splitting inconsistency	The treatment effects were not significantly influenced by clinical modifiers in the subgroup analyses	Undetectable by the routine method The comparison-adjusted funnel plot for the network is not suggestive of any dominant publication bias	Very Low (Downgrade by three levels due to study limitations (for two levels), imprecision)
Oat vs Control	27.2% of the estimate from studies at high risk, and 27.2% at moderate risk	MD -0.01, 95% CI (-0.04,0.03)	Low heterogeneity according to I^2 (42.1%) and *P* (0.12) in direct comparisons No inconsistency between the direct and indirect estimate (Node-split *P*= 0.97 and *tau*= 0.07)	The treatment effects were not significantly influenced by clinical modifiers in the subgroup analyses	Undetectable by the routine method The comparison-adjusted funnel plot for the network is not suggestive of any dominant publication bias	Low (Downgrade by two levels due to study limitations and imprecision)

Supplemental Table 58 Continued

Comparisons	Study limitations	Imprecision	Heterogeneity and Inconsistency	Indirectness	Publication bias	Confidence in MD for overall change in TG
Oat bran vs Control	0% of the estimate from studies at high risk, and 100% at moderate risk	MD -0.08, 95% CI (-0.12,-0.03)	Moderate heterogeneity according to I^2 (65.5%) and *P* (0.94) in direct comparisons No inconsistency between the direct and indirect estimate (Node-split *P*= 0.78 and *tau*= 0.07)	The treatment effects were significantly influenced by some clinical modifiers in the subgroup analyses	Undetectable by the routine method The comparison-adjusted funnel plot for the network is not suggestive of any dominant publication bias	Moderate (Downgrade by three levels due to indirectness)
Wheat vs Control	50% of the estimate from studies at high risk, and 25% at moderate risk	MD -0.03, 95% CI (-0.09,0.03)	Mild heterogeneity according to I^2 (0%) and *P* (1.00) in direct comparisons No inconsistency between the direct and indirect estimate (Node-split *P*= 0.90 and *tau*= 0.07)	The treatment effects were not significantly influenced by clinical modifiers in the subgroup analyses	Undetectable by the routine method The comparison-adjusted funnel plot for the network is not suggestive of any dominant publication bias	Low (Downgrade by two levels due to study limitations and imprecision)
Wheat bran vs Control	50% of the estimate from studies at high risk, and 50% at moderate risk	MD , 95% CI -0.12 (-0.19,-0.05)	Mild heterogeneity according to I^2 (0%) and *P* (0.34) in direct comparisons No inconsistency between the direct and indirect estimate (Node-split *P*= 0.90 and *tau*= 0.07)	The treatment effects were not significantly influenced by clinical modifiers in the subgroup analyses	Undetectable by the routine method The comparison-adjusted funnel plot for the network is not suggestive of any dominant publication bias	Low (Downgrade by two levels due to study limitations and imprecision)

Supplemental Table 58 Continued

Comparisons	Study limitations	Imprecision	Heterogeneity and Inconsistency	Indirectness	Publication bias	Confidence in MD for overall change in TG
Barley vs Oat	0% of the estimate from studies at high risk, and 100% at moderate risk	MD -0.01, 95% CI (-0.09,0.06)	Low heterogeneity according to I^2 (41.4%) and *P* (0.52) in direct comparisons No inconsistency between the direct and indirect estimate (Node-split *P*= 0.81 and *tau*= 0.07)	The treatment effects were not significantly influenced by clinical modifiers in the subgroup analyses	Undetectable by the routine method The comparison-adjusted funnel plot for the network is not suggestive of any dominant publication bias	Moderate (Downgrade by one level due to imprecision)
Barley vs Wheat	0% of the estimate from studies at high risk, and 100% at moderate risk	MD 0.01, 95% CI (-0.08,0.10)	Only one head-to-head study, and no heterogeneity No inconsistency between the direct and indirect estimate (Node-split *P*= 0.87 and *tau*=0.07)	The treatment effects were not significantly influenced by clinical modifiers in the subgroup analyses	Undetectable by the routine method The comparison-adjusted funnel plot for the network is not suggestive of any dominant publication bias	Moderate (Downgrade by one level due to imprecision)
Oat vs Oat bran	0% of the estimate from studies at high risk, and 100% at moderate risk	MD 0.07, 95% CI (0.02,0.12)	Mild heterogeneity according to I^2 (0%) and *P* (0.88) in direct comparisons No inconsistency between the direct and indirect estimate (Node-split *P*= 0.44 and *tau*= 0.07)	The treatment effects were not significantly influenced by clinical modifiers in the subgroup analyses	Undetectable by the routine method The comparison-adjusted funnel plot for the network is not suggestive of any dominant publication bias	High

Supplemental Table 58 Continued

Comparisons	Study limitations	Imprecision	Heterogeneity and Inconsistency	Indirectness	Publication bias	Confidence in MD for overall change in TG
Oat vs Wheat	0% of the estimate from studies at high risk, and 100% at moderate risk	MD 0.02, 95% CI (-0.04,0.09)	High heterogeneity according to I^2 (77.6%) and *P* (0.02) in direct comparisons No inconsistency between the direct and indirect estimate (Node-split *P*= 0.95 and *tau*= 0.07)	The treatment effects were not significantly influenced by clinical modifiers in the subgroup analyses	Undetectable by the routine method The comparison-adjusted funnel plot for the network is not suggestive of any dominant publication bias	Low (Downgrade by two levels due to imprecision and Heterogeneity and Inconsistency)
Oat bran vs Wheat	0% of the estimate from studies at high risk, and 50% at moderate risk	MD -0.05, 95% CI (-0.12,0.02)	Mild heterogeneity according to I^2 (0%) and *P* (0.54) in direct comparisons No inconsistency between the direct and indirect estimate (Node-split *P*= 0.79 and *tau*= 0.07)	The treatment effects were not significantly influenced by clinical modifiers in the subgroup analyses	Undetectable by the routine method The comparison-adjusted funnel plot for the network is not suggestive of any dominant publication bias	Moderate (Downgrade by one level due to imprecision)
Oat bran vs Wheat bran	0% of the estimate from studies at high risk, and 100% at moderate risk	MD 0.04, 95% CI (-0.02,0.10)	Moderate heterogeneity according to I^2 (57.7%) and *P* (0.05) in direct comparisons No inconsistency between the direct and indirect estimate (Node-split *P*= 0.78 and *tau*= 0.07)	The treatment effects were significantly influenced by clinical modifiers in the subgroup analyses	Undetectable by the routine method The comparison-adjusted funnel plot for the network is not suggestive of any dominant publication bias	Low (Downgrade by two levels due to imprecision and indirectness)

[a]The quality of evidence of network estimates for all outcomes by using the GRADE framework, which characterizes the quality of a body of evidence on the basis of the study limitations, imprecision, inconsistency, indirectness, and publication bias.
